# Supplementary material for: Influence of the chirality of carbon nanodots on their interaction with proteins and cells
Source: Nat Commun. 2021 Dec 10;12:7208. doi: 10.1038/s41467-021-27406-1 (PMC8664908; doi:10.1038/s41467-021-27406-1)
Supplement: Supplementary file 1 — Supplementary Information [file 41467_2021_27406_MOESM1_ESM.docx]

**Supplementary Information**

**Influence of the chirality of carbon nanodots on their interaction with proteins and cells**

Huijie Yan, Michele Cacioppo, Saad Megahed, Francesca Arcudi, Luka Đorđević , Dingcheng Zhu, Florian Schulz, Maurizio Prato^*^, Wolfgang J. Parak^*^, Neus Feliu^*^

**Supplementary Figures**


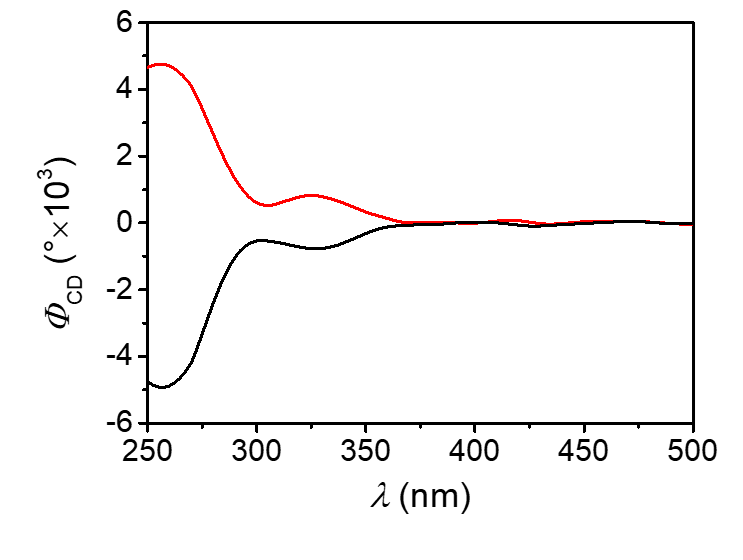


**Supplementary Figure 1.** **(Chiro)ptical characterization of *R*- and *S*-CNDs in Milli-Q water.** Electronic circular dichroism (ECD) spectra of *R-*CNDs (black line) and *S-*CNDs (red line) in water at 298 K. The results are in agreement with our previous work.^1^


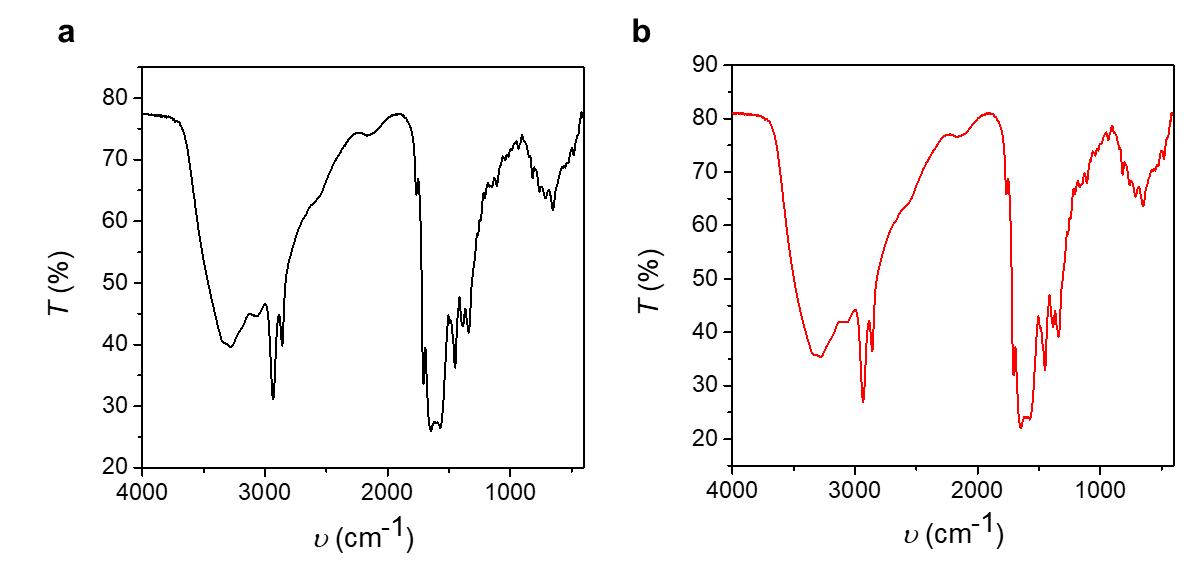


**Supplementary Figure 2.** **FT-IR spectra of *R*- and *S*-CNDs**. **a**) *R-*CNDs. (**b**) *S-*CNDs. The results are in agreement with our previous work.^1^


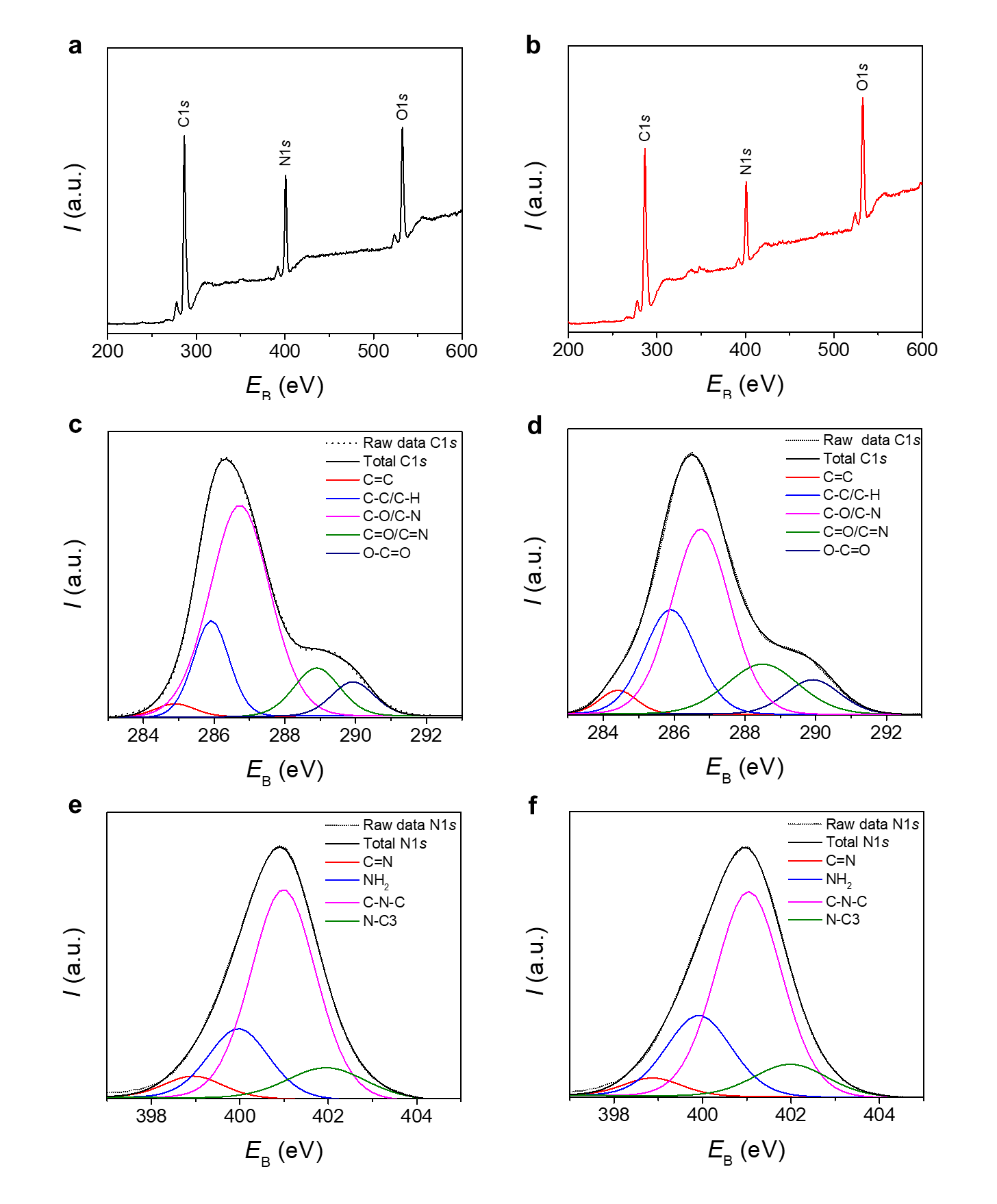


**Supplementary Figure 3.** **XPS of *R*- and *S*-CNDs.** XPS survey of (**a**) *R-*CNDs and (**b**) *S-*CNDs showing the C1*s*, N1*s* and O1*s*; deconvoluted C1*s* spectra of (**c**) *R-*CNDs and (**d**) *S-*CNDs; deconvoluted N1*s* spectra of (**e**) *R-*CNDs and (**f**) *S-*CNDs. I = intensity. E_B_ = binding energy. The results are in agreement with our previous work.^1^

**
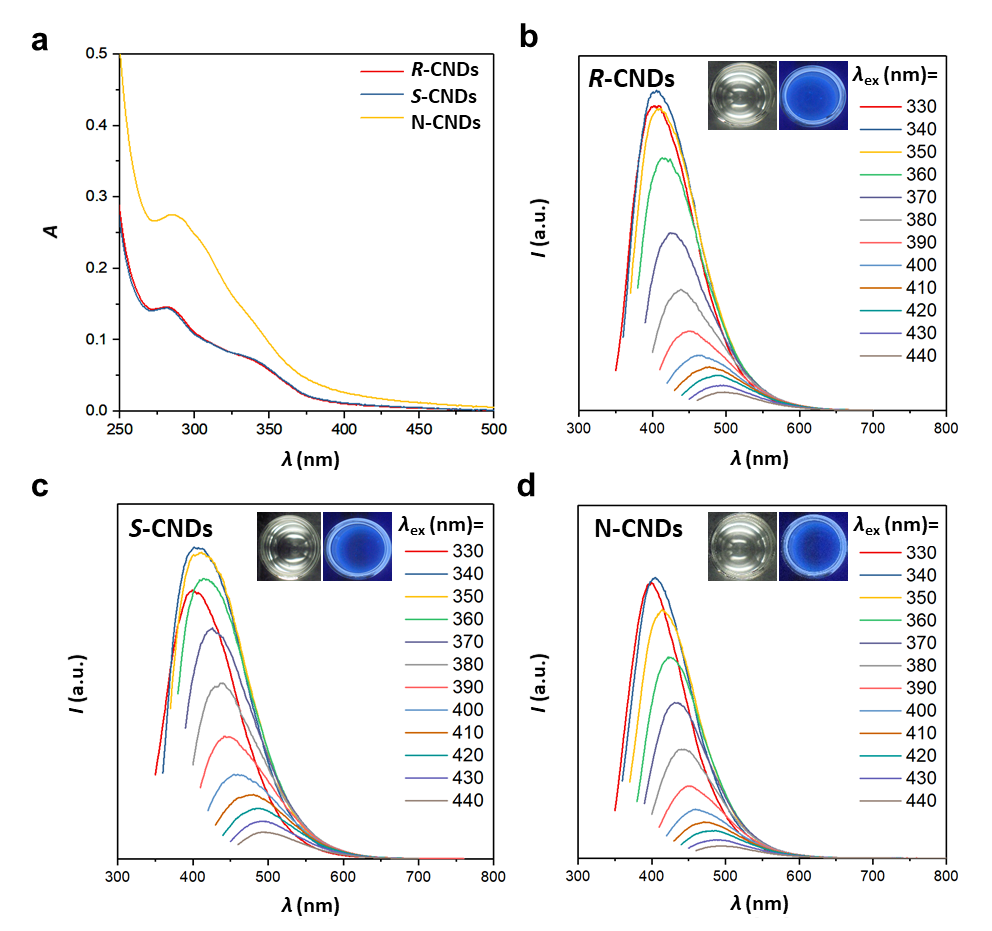
**

**Supplementary Figure 4. Optical characterization of *R-*, *S-* and N*-*CNDs in Milli-Q water.** (**a**) UV-Vis absorption spectra at a concentration of *C*_CNDs_ = 100 µg mL^–1^; (**b**,**c**,**d**) Fluorescence emission spectra of *R-*CNDs, *S-*CNDs, and N*-*CNDs in dependence of the excitation wavelength *λ*_ex_. Experiments performed in Milli-Q water at 298 K. The insets show images of the CNDs solutions taken under white light (left) and UV light (right) exposure. The data of the CNDs shown here were recorded on batch #1.


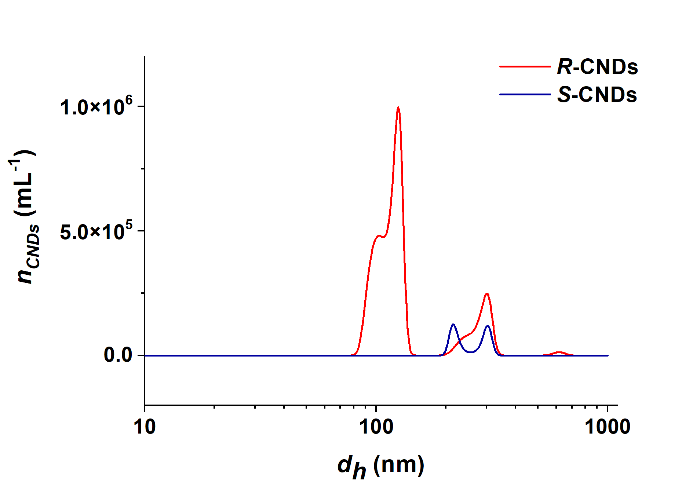


**Supplementary Figure 5.** **Nanoparticle tracking analysis of *R*- and *S*-CNDs.** Apparent hydrodynamic diameters *d*_h_ of tracked CNDs (aggregates) for *R-*CNDs (green line) and *S-*CNDs (red line).


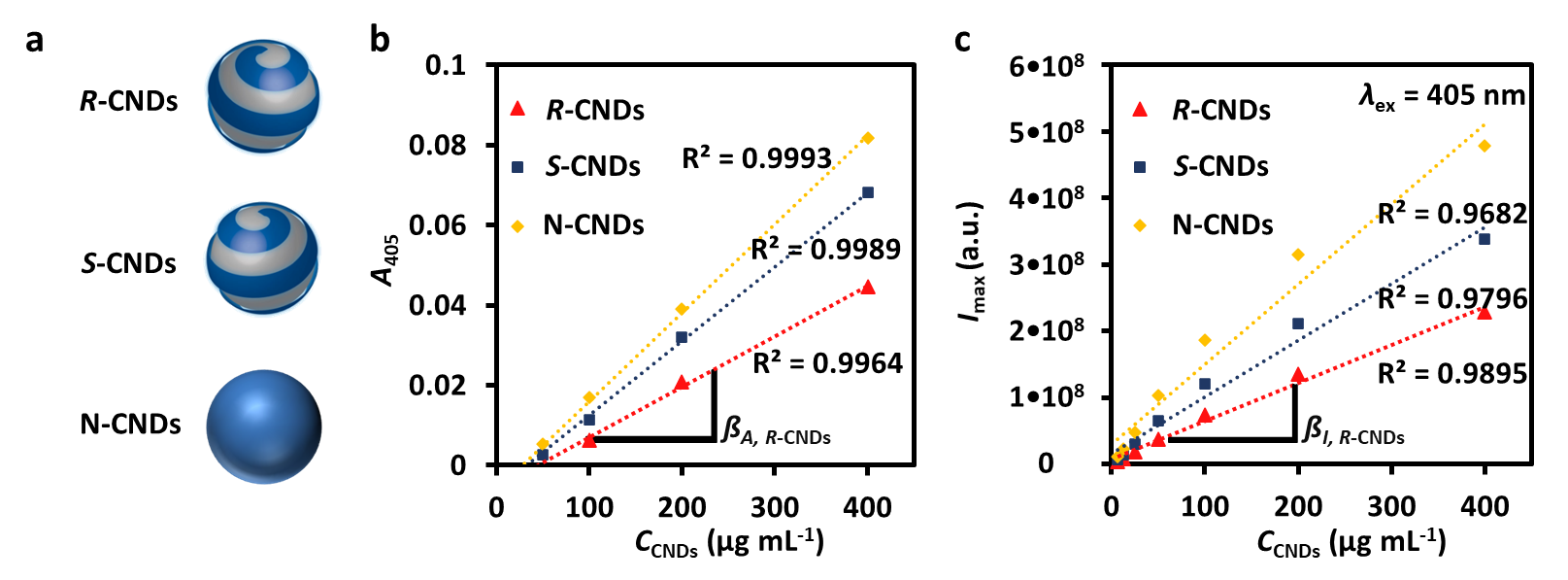


**Supplementary Figure 6.** **CNDs concentration determination trough UV-Vis and Fluorescence emission spectrophotometry.** (**a**) Sketch of the N*-*, *S-*, and *R-*CNDs. (**b**) Absorption at 405 nm (*A*_405_) of N*-*, *S-*, and *R-*CNDs (in Milli-Q water) at the mass concentration *C*_CNDs_ (determined by weighting) as taken from the data shown in Supplementary Figure 26a. The curves of *A*_405_ as a function of *C*_CNDs_ were fitted with linear regression to yield the slope *ß_A_*_,j_ (mL μg^–1^) = Δ*A*_405_(j)/Δ*C*_CNDs_ (j = *R-*CND, *S-*CND, N*-*CND). R^2^ indicates the fitting reliability with linear regression (perfect fit: R^2^ = 1). (**c**) Integrated fluorescence emission intensity (*I*_max_) ranging from 425-475 nm (excitation wavelength *λ*_ex_ = 405 nm) of N*-*, *S-*, and *R-*CNDs (in Milli-Q water) at the mass concentration *C*_CNDs_ as taken from the data shown in Supplementary Figure 26b. The curve of *I*_max_ as a function of *C*_CNDs_ were fitted with linear regression to yield the slope *ß_I_*_,j_ (mL μg^–1^) = Δ*I*_max_(j) Δ*C*_CNDs_^–1^ (j = *R-*CND, *S-*CND, N*-*CND). From these slopes, first the percentual differences Δ*ß*_i,j_ in the slopes between the *R-*CND and *S-*CND sample to the N*-*CND sample were derived for the absorption and intensity measurements as Δ*ß*_i,j_ = (*ß*_i,N_*_-_*_CND_ - *ß*_i,j_) *ß*_i,N_*_-_*_CND_^–1^ (i = *A*, *I*; j = *R-*CND, *S-*CND), and then the deviation Δ*ß*_j_ in these differences between the absorption and intensity measurements were obtained as Δ*ß*_j_ =|Δ*ß_A_*_,j_ - Δ*ß_I_*_,j_| (j = *R-*CND, *S-*CND). The percentual error in concentration determination was defined as the maximum of these values as Δ*C*_CNDs_ *C*_CNDs_^–1^ = max(Δ*ß_R-_*_CND_, Δ*ß_S-_*_CND_). The values are enlisted in Supplementary Table 10. The data were recorded with batch #1. The data shown in Supplementary Figure 6c are the same than those in Figure 1c of the main manuscript.


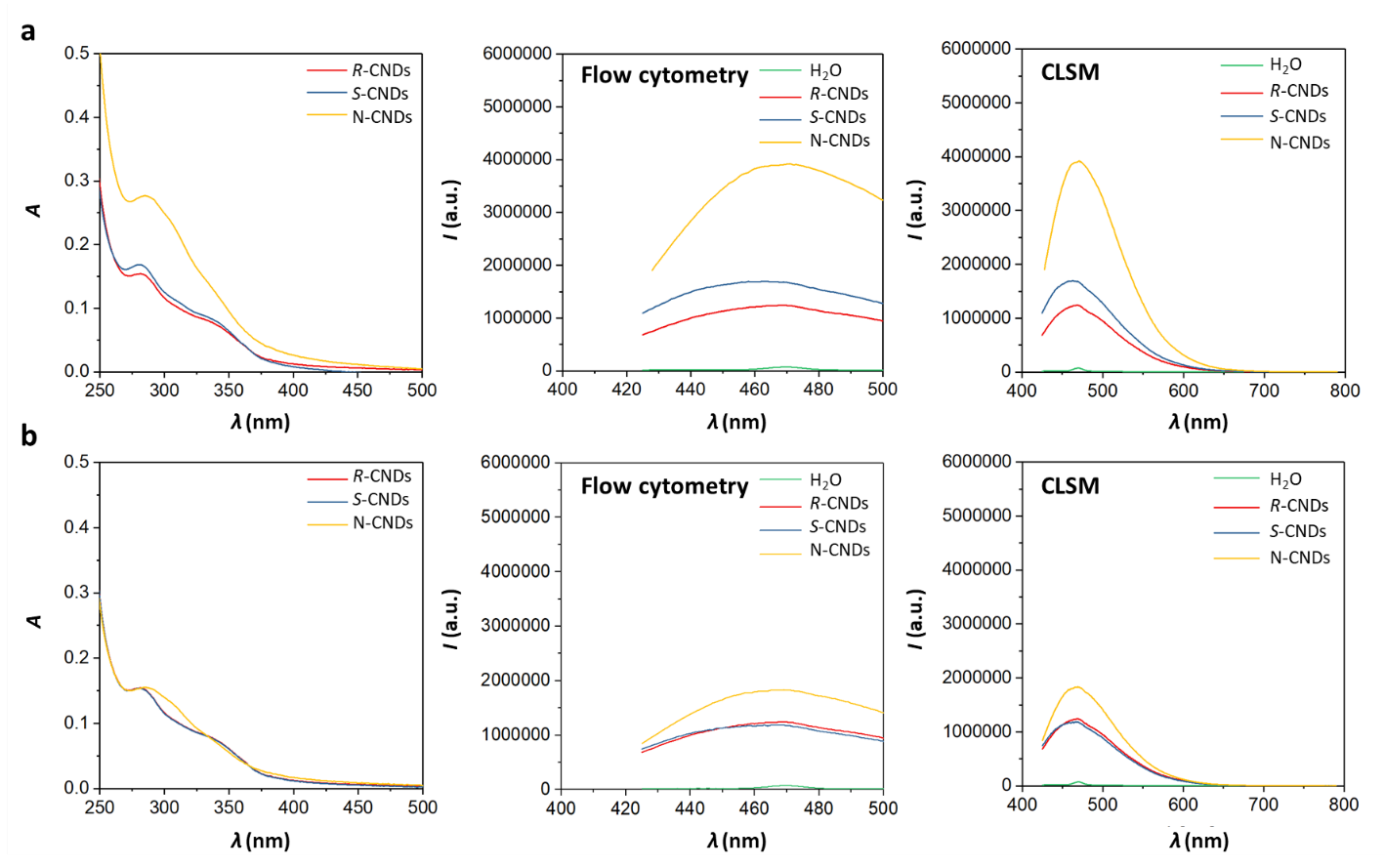


**Supplementary Figure 7. CNDs UV-Vis and fluorescence spectra.** (**a**) UV-Vis absorption and fluorescence spectra (*λ*_ex_ = 405 nm) of N*-*, *S-*, and *R-*CNDs in Milli-Q water at 298 K at concentration *C*_CNDs_ = 100 µg mL^–1^ (as originally determined by weighting). The fluorescence spectra are displayed in two different ranges: from 425 nm to 500 nm, which emulates the 450 nm band pass filter with 50 nm width used later in the flow cytometer, and from 425 nm to 800 nm, which emulated the 420 nm long pass filter as later used with confocal microscopy. Fluorescence measurements of water that was used as blank are reported. (**b**) Same data set of (a) where the *S-*CND and N*-*CND samples were further diluted with Milli-Q water until they had the same adsorption at 280 nm as the *R-*CND sample. In this way, the adjusted concentration of all three samples is defined as *C*'_CNDs_ = 100 µg mL^–1^. UV-Vis absorption and fluorescence intensity spectra (*λ*_ex_ = 405 nm) were recorded as showed in (a).


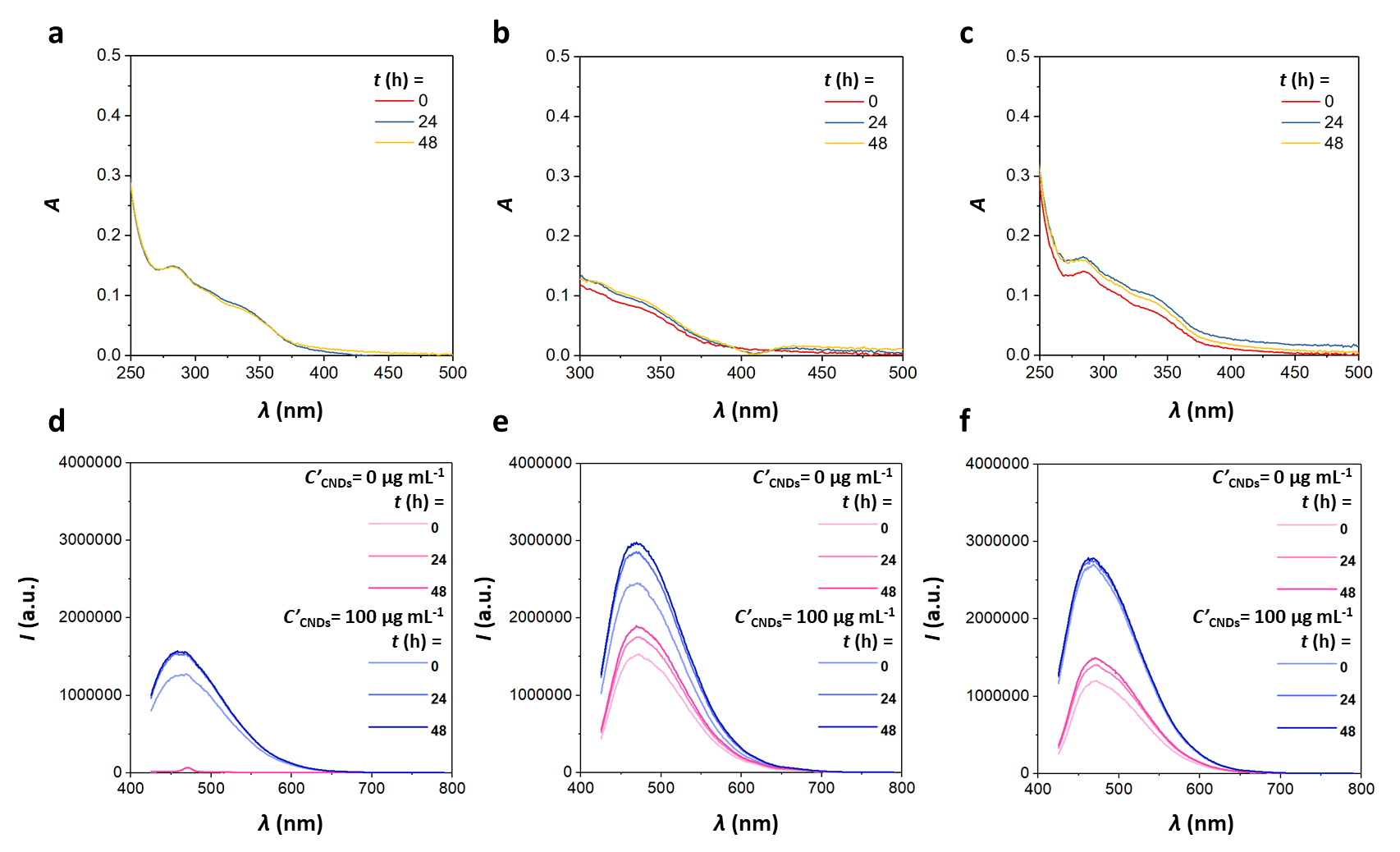


**Supplementary Figure 8. CNDs UV-Vis and fluorescence spectra.** UV-Vis absorption and fluorescence spectra (*λ*_ex_ = 405 nm) of *R-*CNDs after incubation for 0, 24 or 48 h with (**a**,**d**) H_2_O, (**b**,**e**) RPMI 1640 medium without phenol red containing 10% FBS, and (**c**,**f**) RPMI 1640 medium without phenol red without serum supplement measured. Data were recorded with batch #1. In the fluorescence spectra (**d**,**e**,**f**) the pink curves are the blanks (no CNDs) that show the autofluorescence of the medium and the blue curves originate from *R-*CNDs dissolved in water or medium.


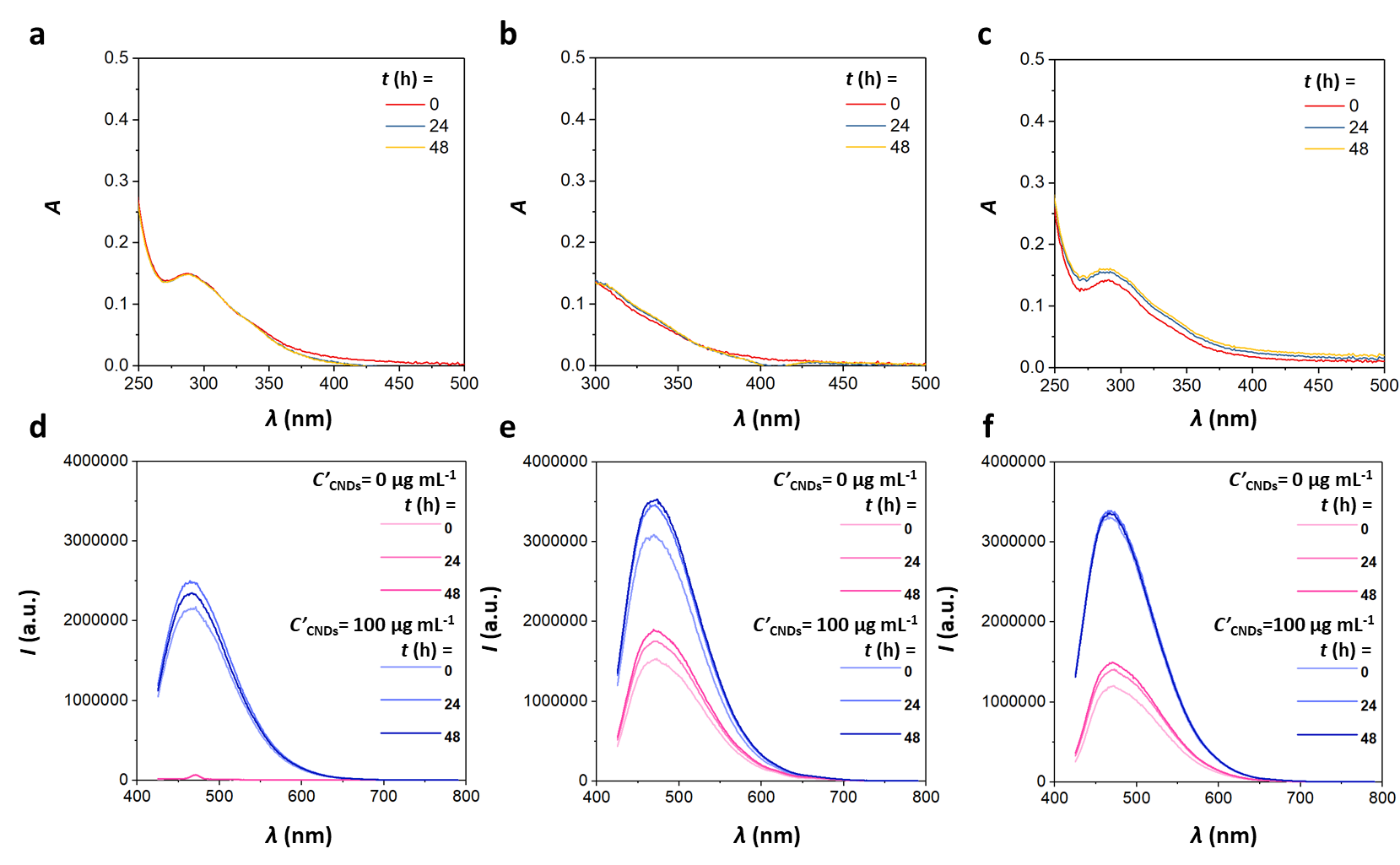


**Supplementary Figure 9. CNDs time- and concentration-dependent UV-Vis and fluorescence spectra.** UV-Vis absorption and fluorescence spectra (*λ*_ex_ = 405 nm) of *N-*CNDs after incubation for 0, 24 or 48 h with (**a**,**d**) H_2_O, b,e) RPMI 1640 medium without phenol red containing 10% FBS, and (**c**,**f**) RPMI 1640 medium without phenol red without serum supplement measured. Data were recorded with batch #1. In the fluorescence spectra (**d**,**e**,**f**) the pink curves are the blanks that show the autofluorescence of the medium and the blue curves originate from *R-*CNDs dissolved in water or medium.


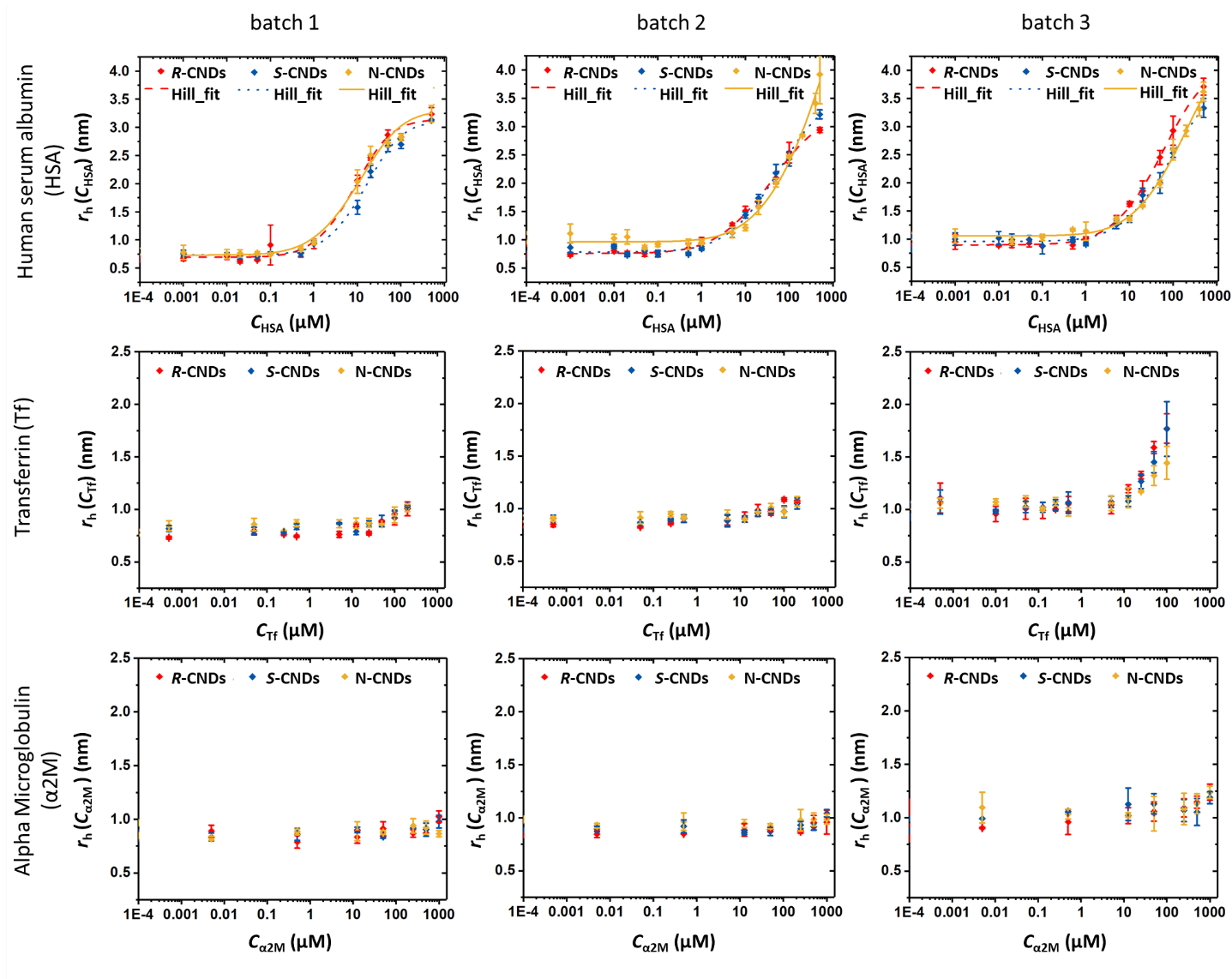


**Supplementary Figure 10. Change of hydrodynamic radius *r*_h_ of *R-*, *S-* and N*-*CNDs in presence of proteins**. Three different batches of CNDs are measured in phosphate buffered saline (PBS) with the presence of (**a**) human serum albumin (HSA), (**b**) transferrin (Tf), and (**c**) alpha microglobulin (α2M) in dependence of the protein concentrations *c*_HSA_ , *c*_Tf_, and *c*_α2M_, respectively. From the plots the fit parameters *K_D_*, *N*_max_, *n*, *r*_h,0_, and Δ*r*_h,max_ were obtained (listed in Supplementary Table 5). *K_D_* is the apparent dissociation constant of the CND-protein complex, *N*_max_ is the maximum number of bound proteins per CND under saturation conditions, *n* is the Hill coefficient, *r*_h,0_ is the hydrodynamic radius of the CNDs without attached proteins, and Δ*r*_h,max_ is the difference in effective hydrodynamic radius between CNDs saturated with proteins and CNDs without attached proteins. The data for batch #1 are shown also in Figure 2 of the main manuscript. Results are shown as mean values with error bars (i.e., the corresponding standard deviations) from three independent samples (n = 3) over three independent experiments.


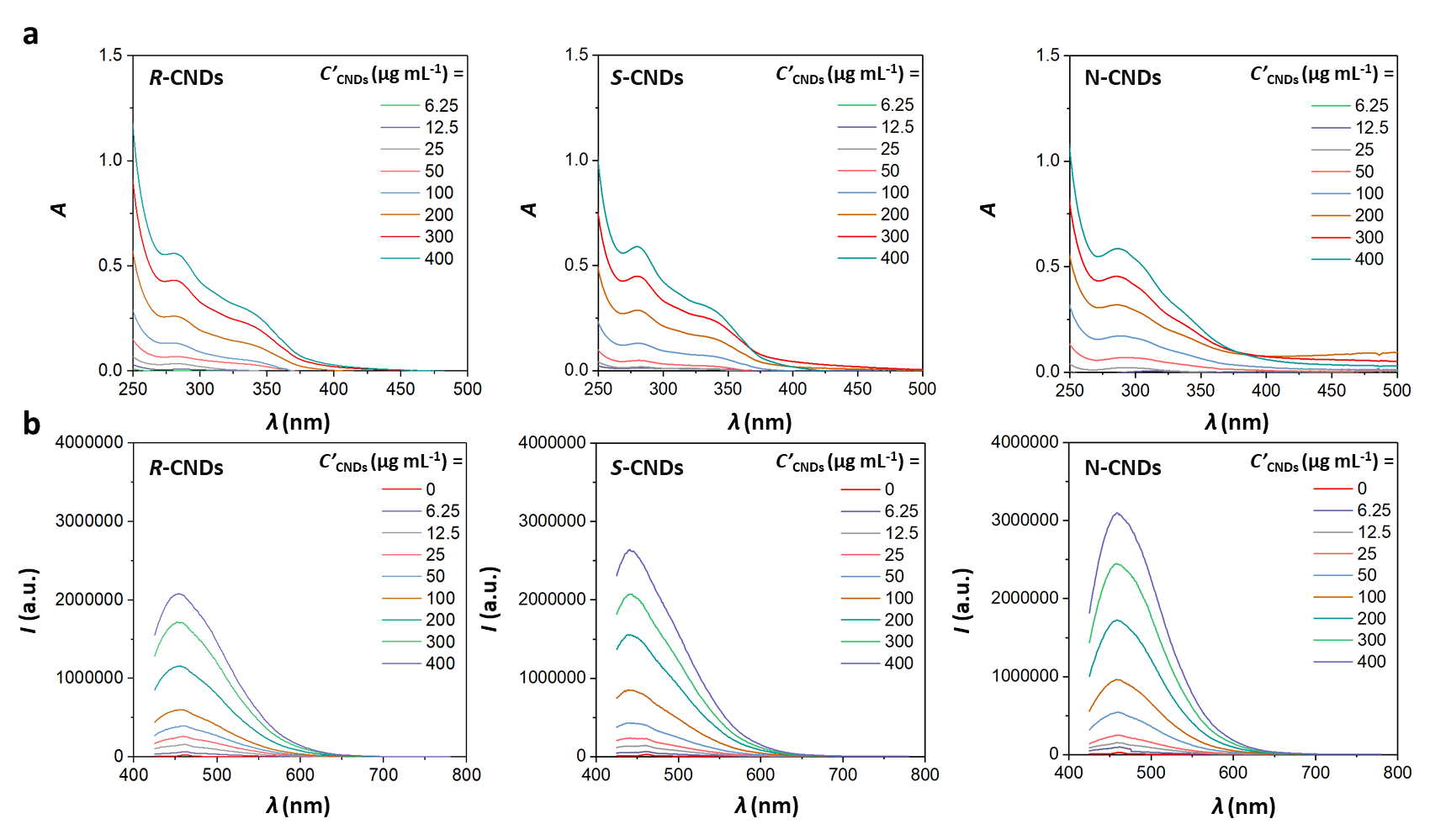


**Supplementary Figure 11. Dose dependent absorbance and fluorescence of *R-*, *S-* and N*-*CNDs at a series of adjusted concentrations *C*'_CNDs_**. (**a**) UV-Vis absorption and (**b**) fluorescence spectra (*λ*_ex_ = 405 nm) of *R-*, *S-* and N*-*CNDs in Milli-Q water at 298 K. Data were recorded with batch #4.

**
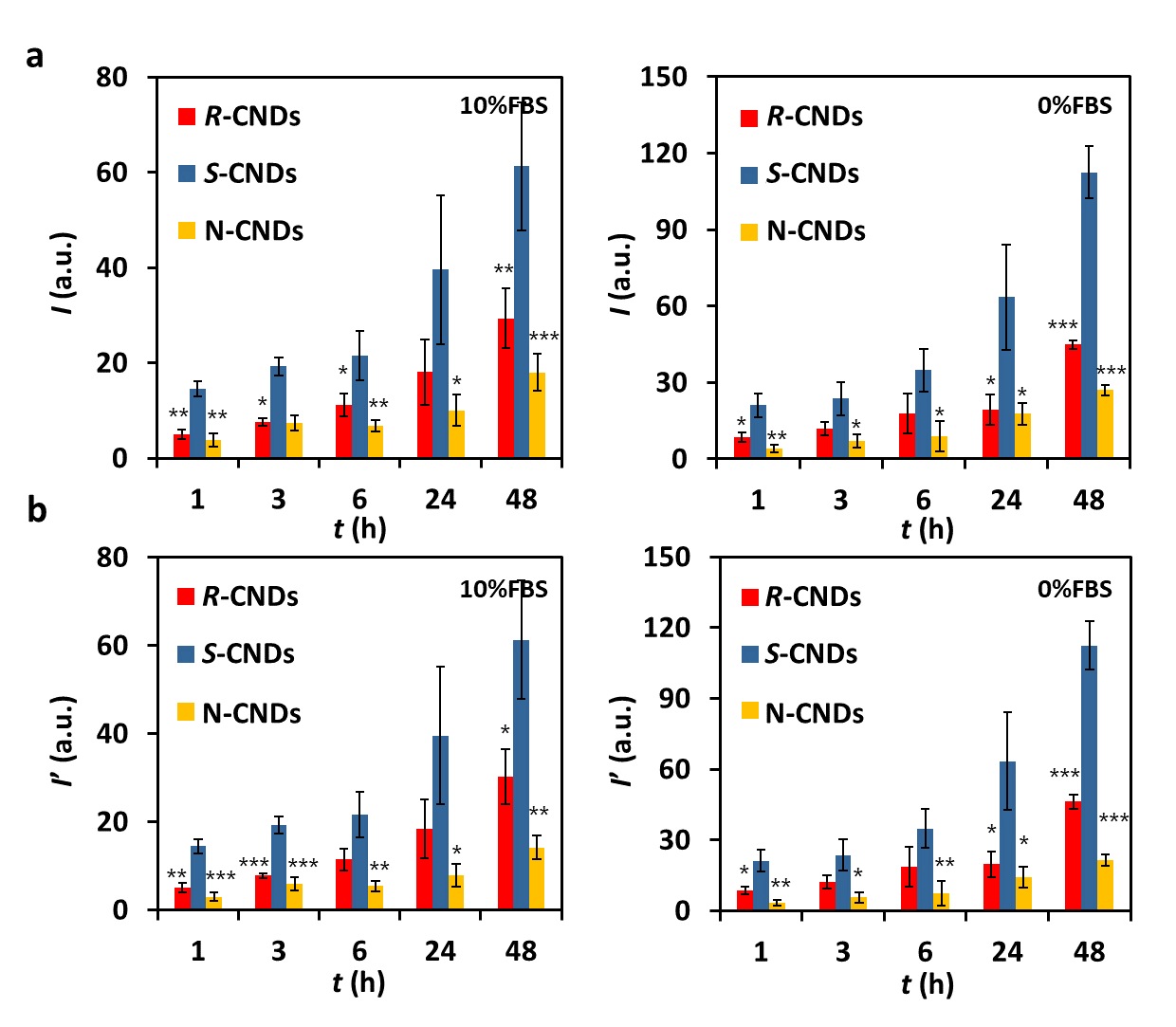
**

**Supplementary Figure 12.** **CNDs time dependent cellular uptake**. (**a**) Quantitative evaluation of cellular uptake of CNDs by THP-1-derived macrophages in terms of mean fluorescence intensity *I* per cell after exposure for the time *t* at the concentration *C*'_CNDs_ = 400 μg mL^–1^ in RPMI 1640 medium with 10% and 0% FBS. (**b**) Mean CND fluorescence per cell *I*' as corrected for the different fluorescence intensities of the different CND samples according to Supplementary Table 4. Results are shown as mean value ± standard deviation (s.d.) from three independent samples (n = 3) over three independent experiments. *P* values were analyzed by Student’s *t*-test with two-tailed distribution and two-sample equal variance. **P*<0.05, ***P*<0.01, ****P*<0.001. Data are enlisted in Supplementary Table 8.

**
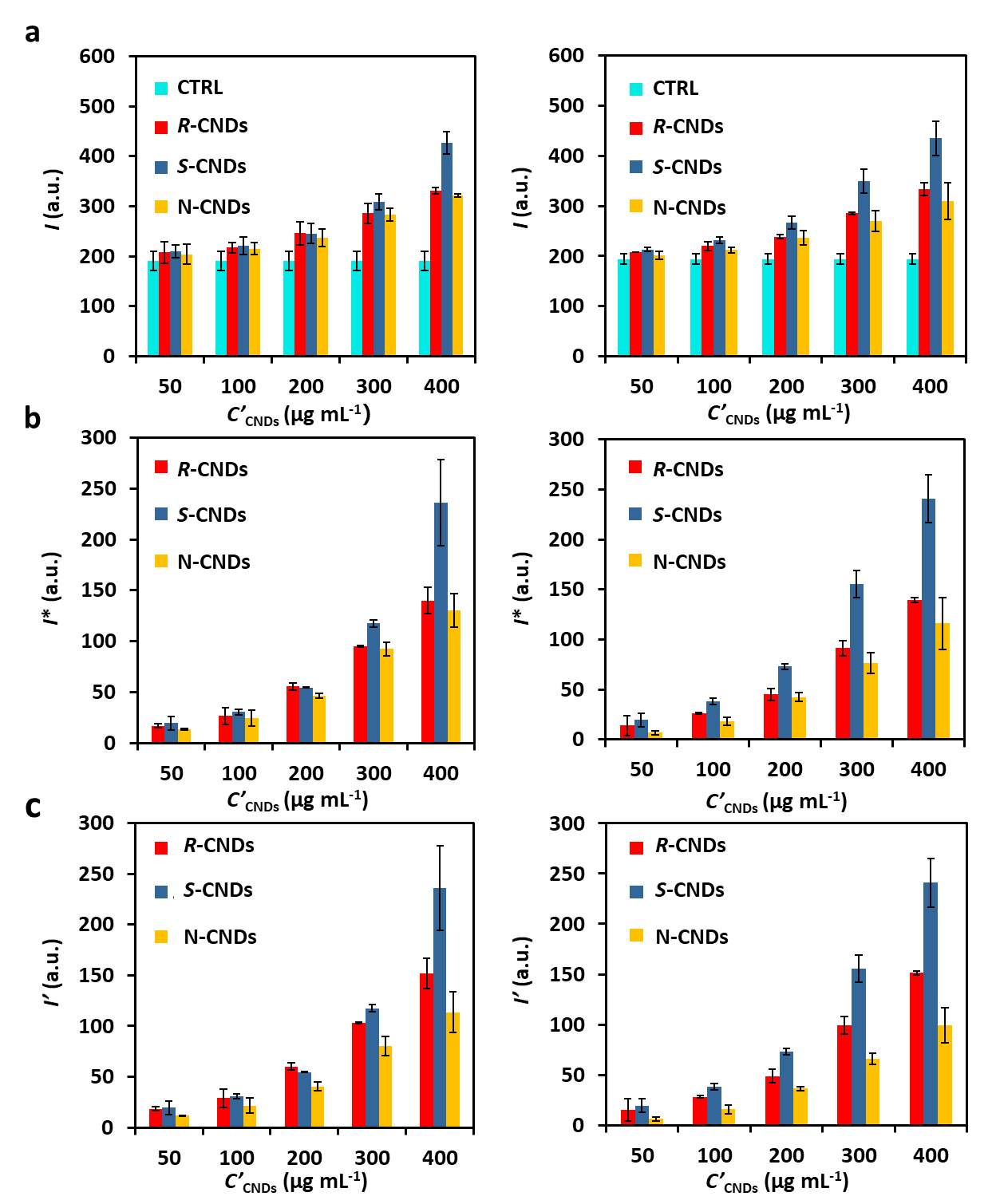
**

**Supplementary Figure 13.** **Mean fluorescence intensity per cell due to internalized *R-*, *S-* and N*-*CNDs as extracted from Supplementary Figure 14**. The left column refers to 10% serum supplement, the right column to serum free conditions. (**a**) Mean fluorescence intensity per cell *I* as detected directly from the flow cytometer data shown in Supplementary Figure 14. (**b**) Mean fluorescence intensity per cell *I*^*^ after background correction. (**c**) Mean fluorescence intensity per cell *I*' after background correction and adjustment for the different fluorescence intensities of each CNDs. Results are shown as mean value ± standard deviation (s.d.) from three independent samples (n = 3) over three independent experiments. The resulting data are enlisted in Supplementary Table 9.

**
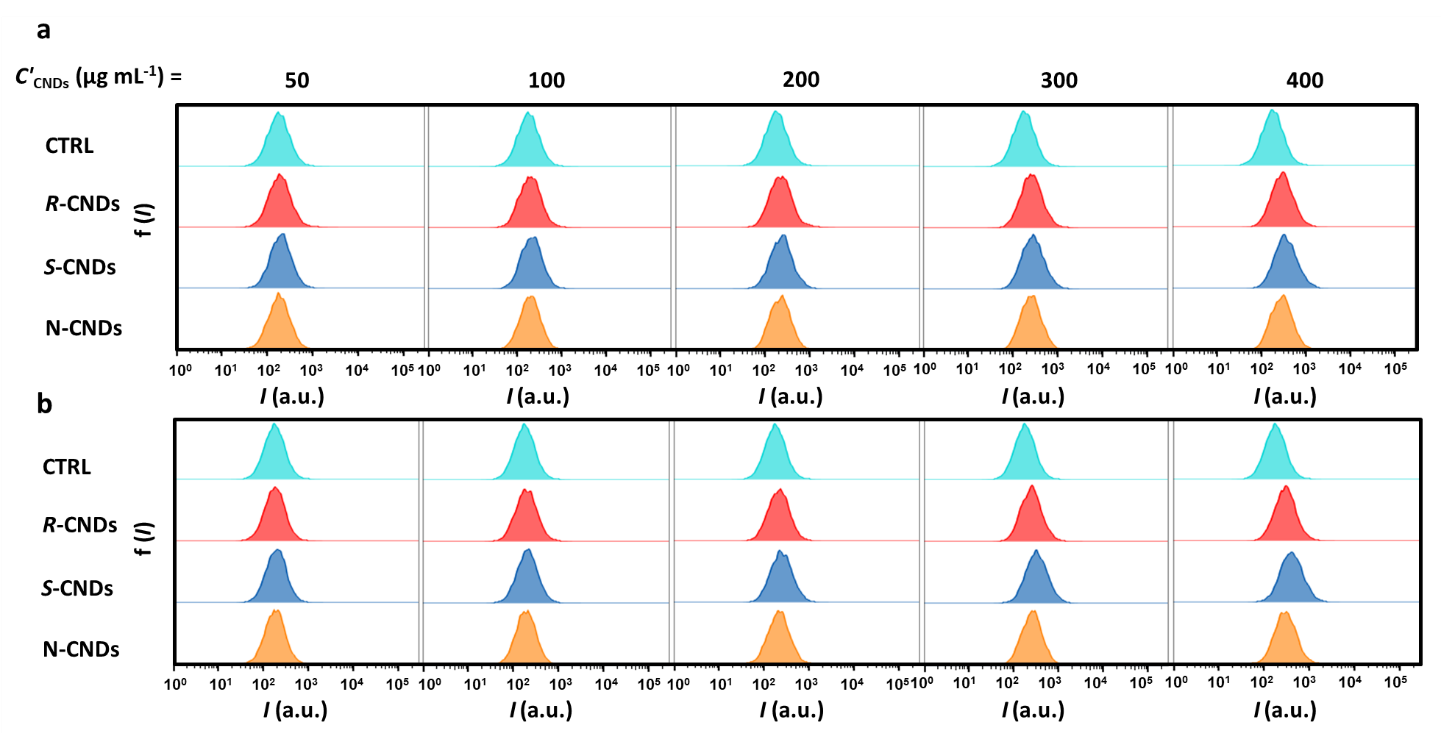
**

**Supplementary Figure 14.** **CNDs concentration-dependent uptake detected by flow cytometry.** Fluorescence distribution f(*I*) per cell of THP-1 derived macrophages after incubation with *R*-, *S*-, and N*-*CNDs for the time *t* = 6 h at different exposure concentrations *C*'_CNDs_ in (**a**) 10% or (**b**) 0% FBS contained RPMI 1640 medium measured by flow cytometry. For the control sample (CTRL) no CNDs were added: *C*'_CNDs_ = 0.

**
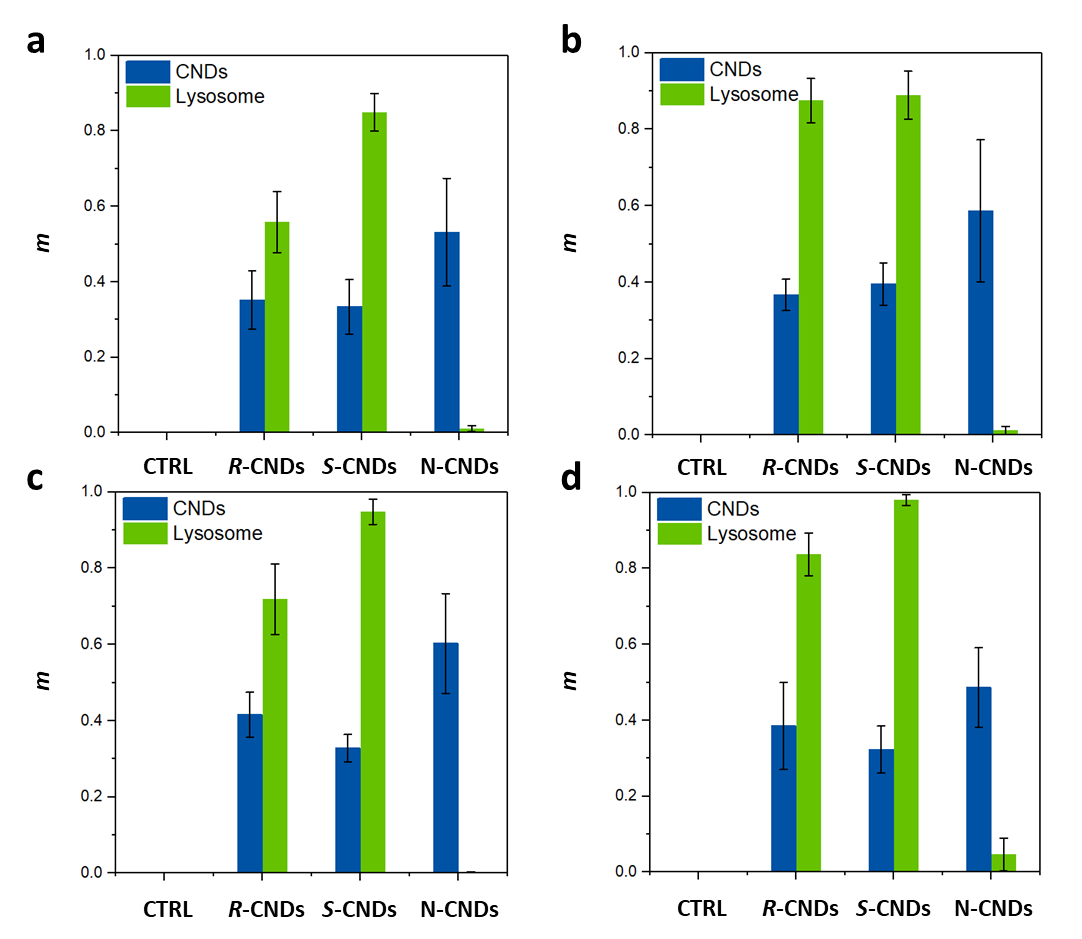
**

**Supplementary Figure 15.** **Overlap degree of CNDs and stained lysosomes (Manders' coefficients) in Hela cells.** (**a**,**b**) 24 or (**c**,**d**) 48 h exposure to CNDs in 10% (**a**,**c**) or 0% (**b**,**d**) FBS containing DMEM medium. Examples for the raw data from which Manders' coefficients have been calculated are provided in Supplementary Figs. 16 and 17. The blue bars show Manders' coefficient *m*_1_, *i.e.* the percentage of blue fluorescent pixels (*i.e.* parts of the cells that contained CNDs) that overlapped with green fluorescent pixels (*i.e.* parts of the cells belonging to lysosomes). The green bars show Manders' coefficient *m*_2_, *i.e.* the percentage of green fluorescent pixels (*i.e.* parts of cells belonging to lysosomes) which overlapped with blue fluorescent pixels (*i.e.* parts of the cells which contained CNDs). The control (CTRL) refers to cells that have not been exposed to CNDs but have been stained with LysoTracker™ Green DND-26. No fluorescence was detected in the control samples. n ≥ 210 cells in 21 images were analyzed for each group. Results are shown as mean values with error bars (i.e., the corresponding standard deviations) from three independent samples (n = 3) over three independent experiments. Data indicate that the CNDs are largely localized in lysosomes (high *m*_1_ values). Note that there is a certain degree of overexposure in the CND channel and thus the fluorescence area of CNDs seems larger than the fluorescence area of lysosomes, which is a clear artifact due to overexposure. *m*_2_ cannot be therefore quantitatively compared to *m*_1_. The data are presented here as a demonstration for potential errors which rule out quantitative analysis. The only conclusion which can be drawn in a non*-*quantitative way is that the CNDs largely co-localize with lysosomes.

**
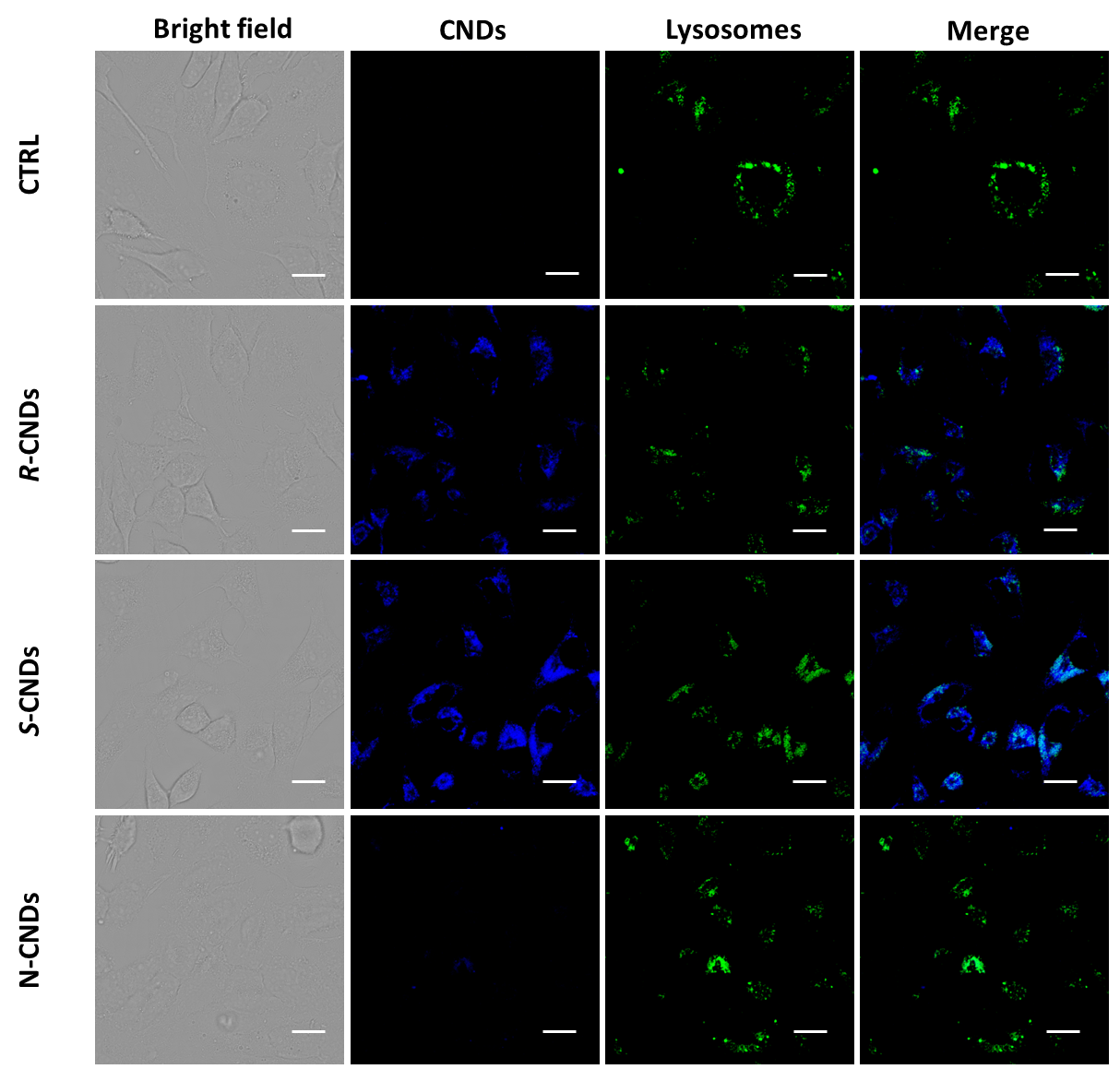
**

**Supplementary Figure 16.** **Lysosomes/CNDs co-localization studies of Hela cells**. Cells were analyzed in 10% FBS containing DMEM medium after 24 h exposure to *R-*, *S-*, and N*-*CNDs (blue fluorescence channel). Lysosomes were stained by LysoTracker™ Green DND-26 (green fluorescence channel). The scale bar represents 20 μm. The experiments were repeated independently for three times (n = 3) and representative images are shown.

**
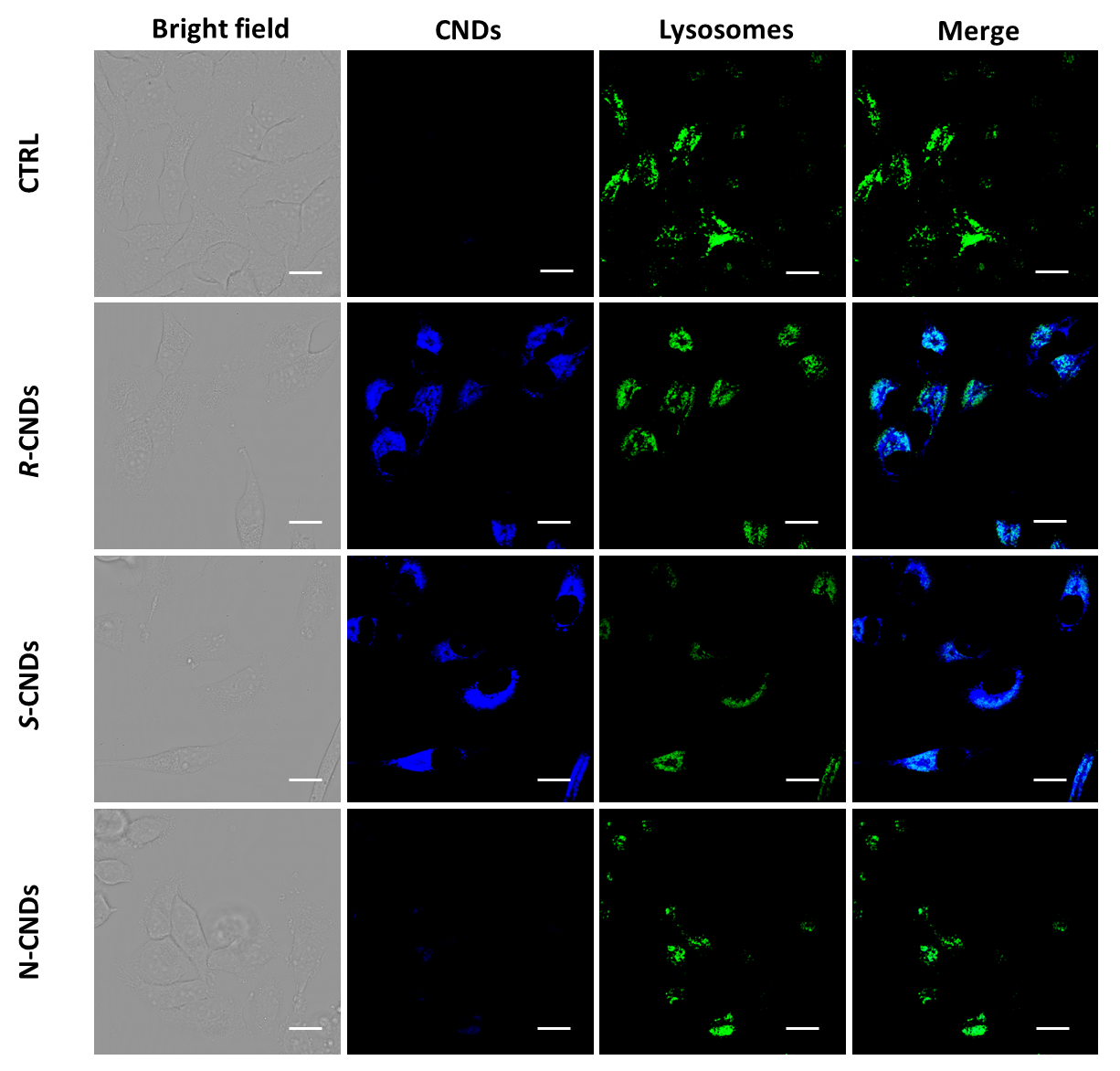
**

**Supplementary Figure 17.** **Lysosomes/CNDs co-localization studies of Hela cells.** Cells were analyzed in serum-free DMEM medium after 48 h exposure to *R-*, *S-*, and N*-*CNDs (blue fluorescence channel). Lysosomes were stained by LysoTracker™ Green DND-26 (green fluorescence channel). The scale bar represents 20 μm. The experiments were repeated independently for three times (n = 3) and representative images are shown.

**
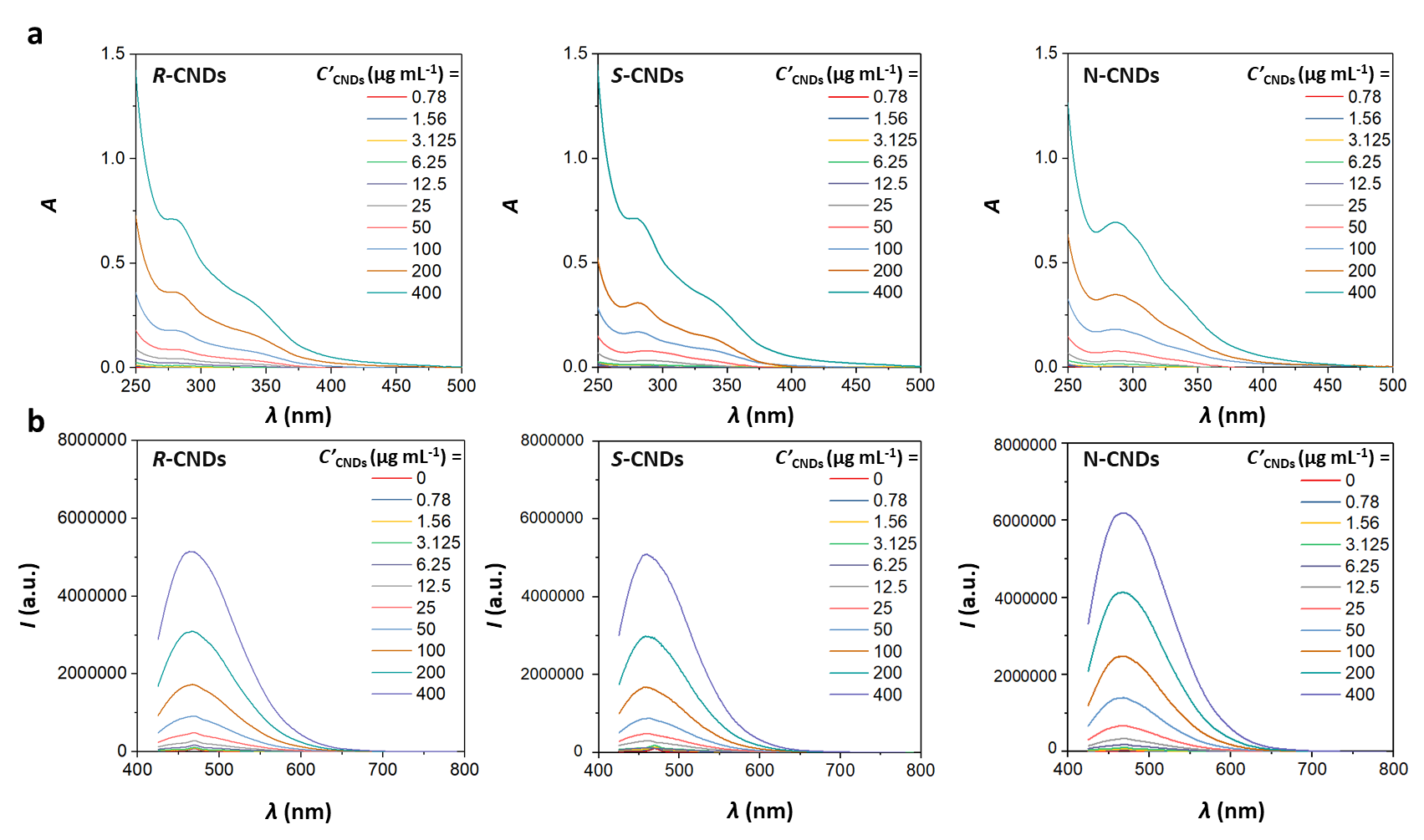
**

**Supplementary Figure 18. Dose dependent absorbance and fluorescence of *R-*, *S-* and N*-*CNDs at a series of adjusted concentrations *C*'_CNDs_**. (**a**) UV-Vis absorption and (**b**) fluorescence spectra (*λ*_ex_ = 405 nm) of *R-*, *S-* and N*-*CNDs in Milli-Q water at 298 K. Data were recorded with batch #1.

**
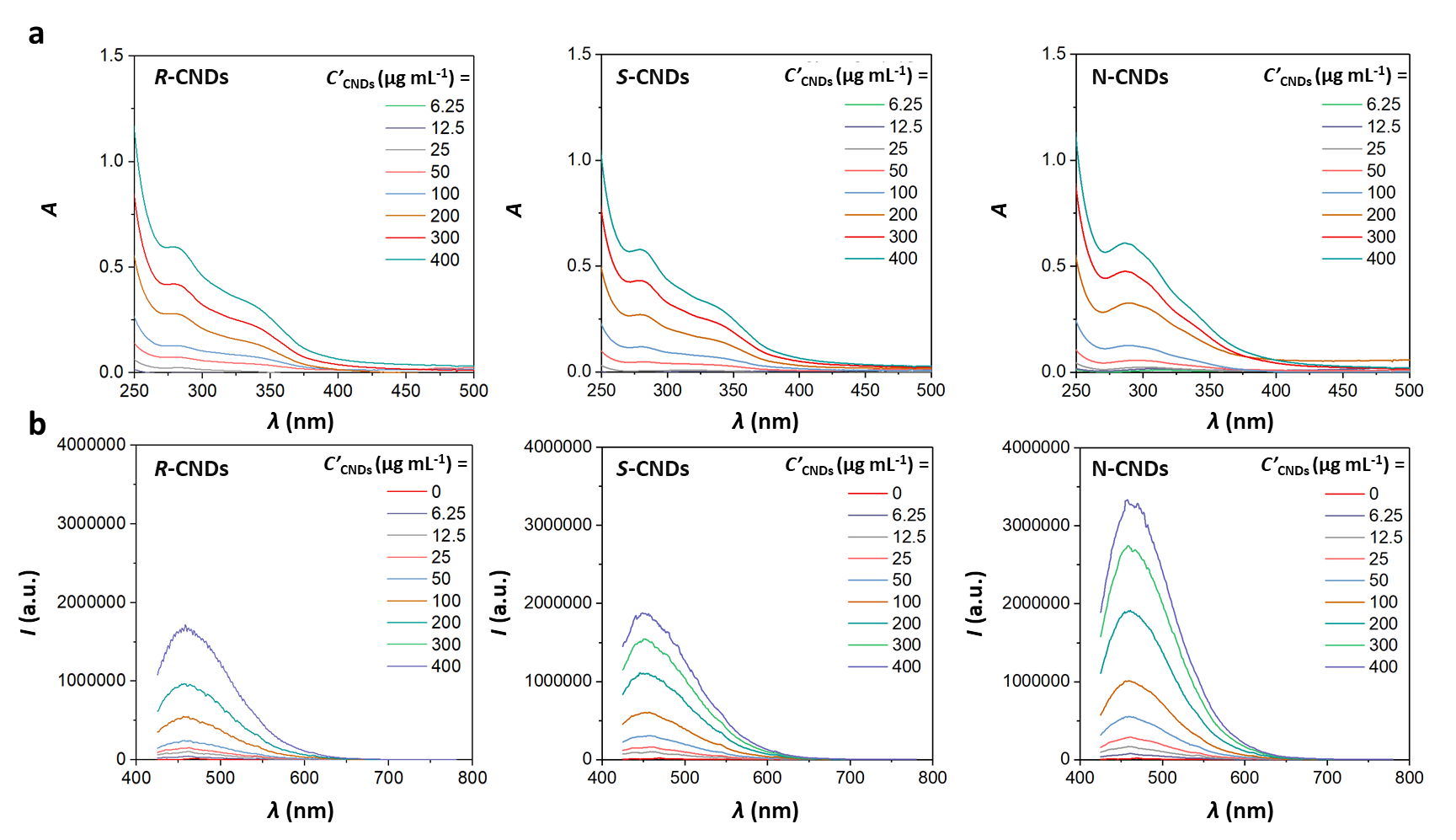
**

**Supplementary Figure 19. Dose dependent absorbance and fluorescence of *R-*, *S-* and N*-*CNDs at a series of adjusted concentrations *C*'_CNDs_.** (**a**) UV-Vis absorption and (**b**) fluorescence spectra (*λ*_ex_ = 405 nm) of *R-*, *S-* and N*-*CNDs in Milli-Q water at 298 K. Data were recorded with batch #5.

**
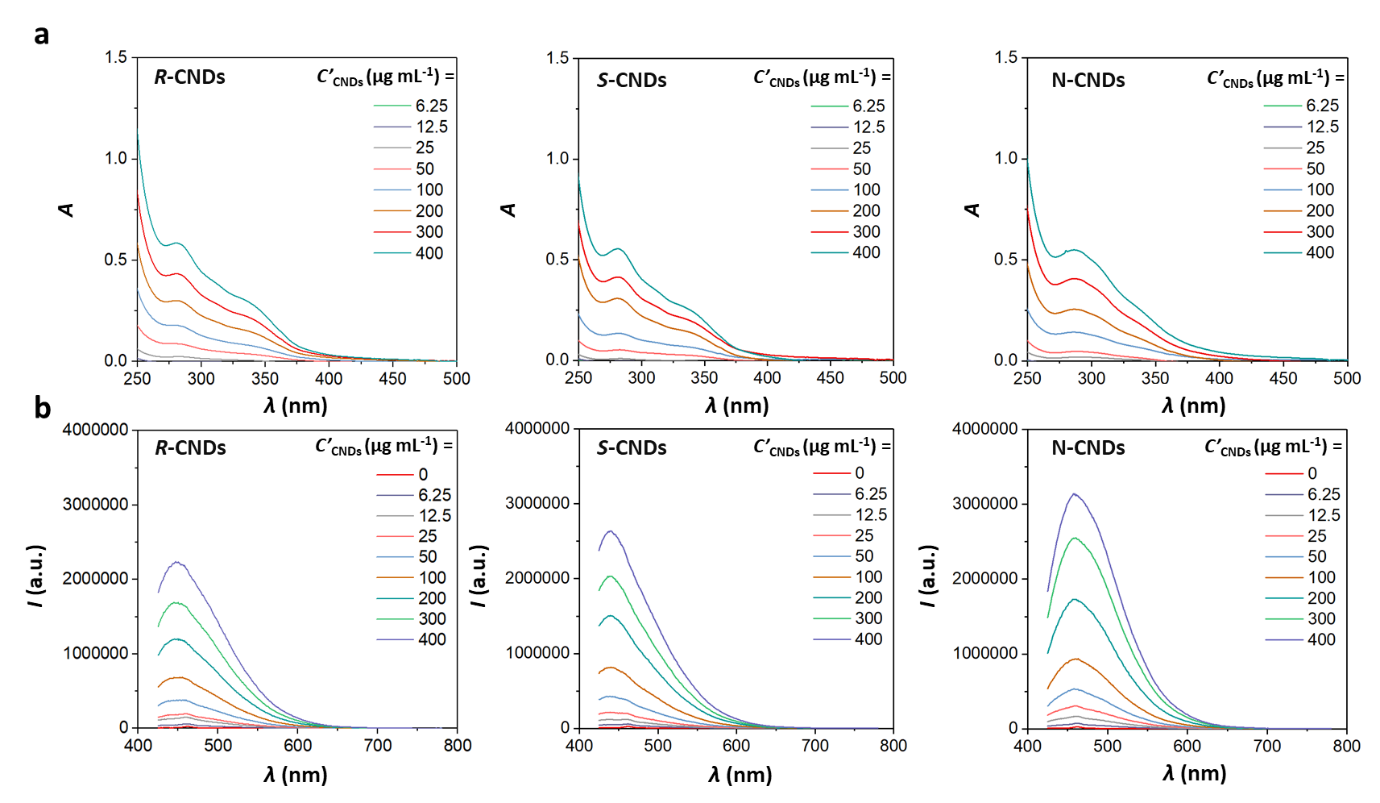
**

**Supplementary Figure 20. Dose dependent absorbance and fluorescence of *R-*, *S-* and N*-*CNDs at a series of adjusted concentrations *C*'_CNDs_.** (**a**) UV-Vis absorption and (**b**) fluorescence spectra (*λ*_ex_ = 405 nm) of *R-*, *S-* and N*-*CNDs in Milli-Q water at 298 K. Data were recorded with batch #2.

**
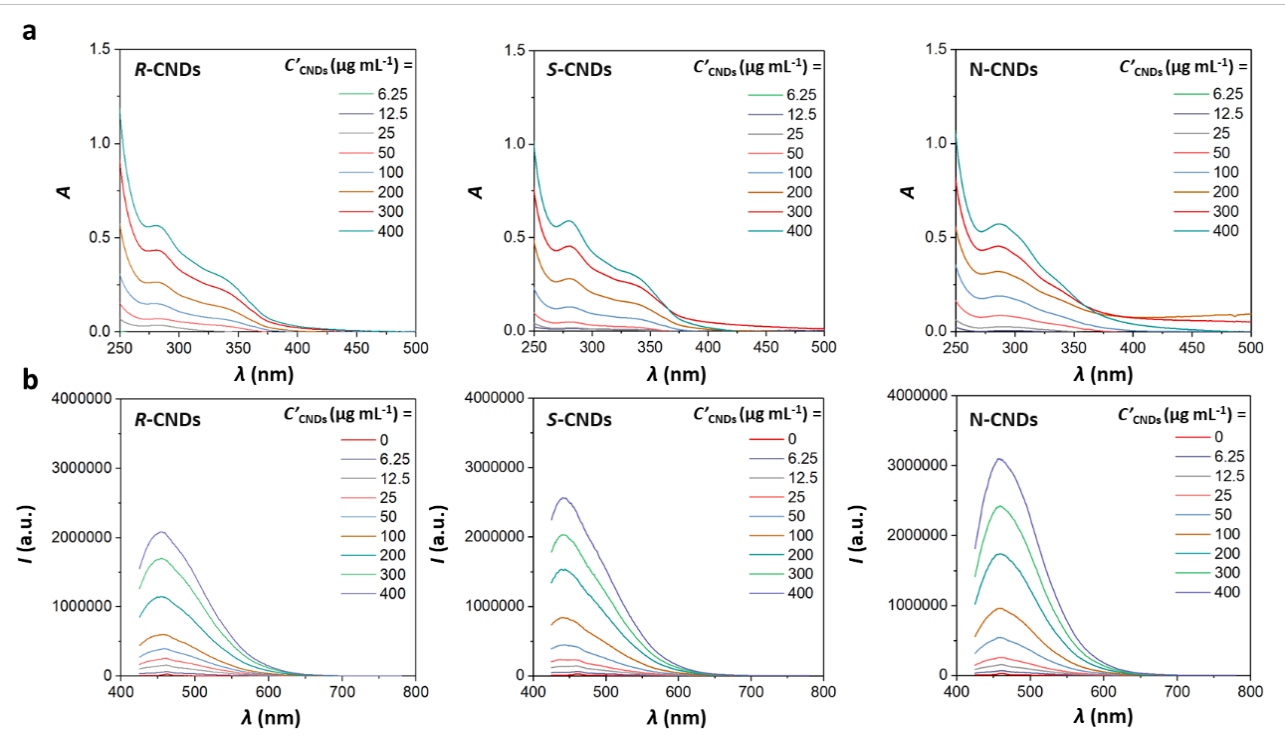
**

**Supplementary Figure 21. Dose dependent absorbance and fluorescence of *R-*, *S-* and N*-*CNDs at a series of adjusted concentrations *C*'_CNDs_.** (**a**) UV-Vis absorption and (**b**) fluorescence spectra (*λ*_ex_ = 405 nm) of *R-*, *S-* and N*-*CNDs in Milli-Q water at 298 K. Data were recorded with batch #3.

**
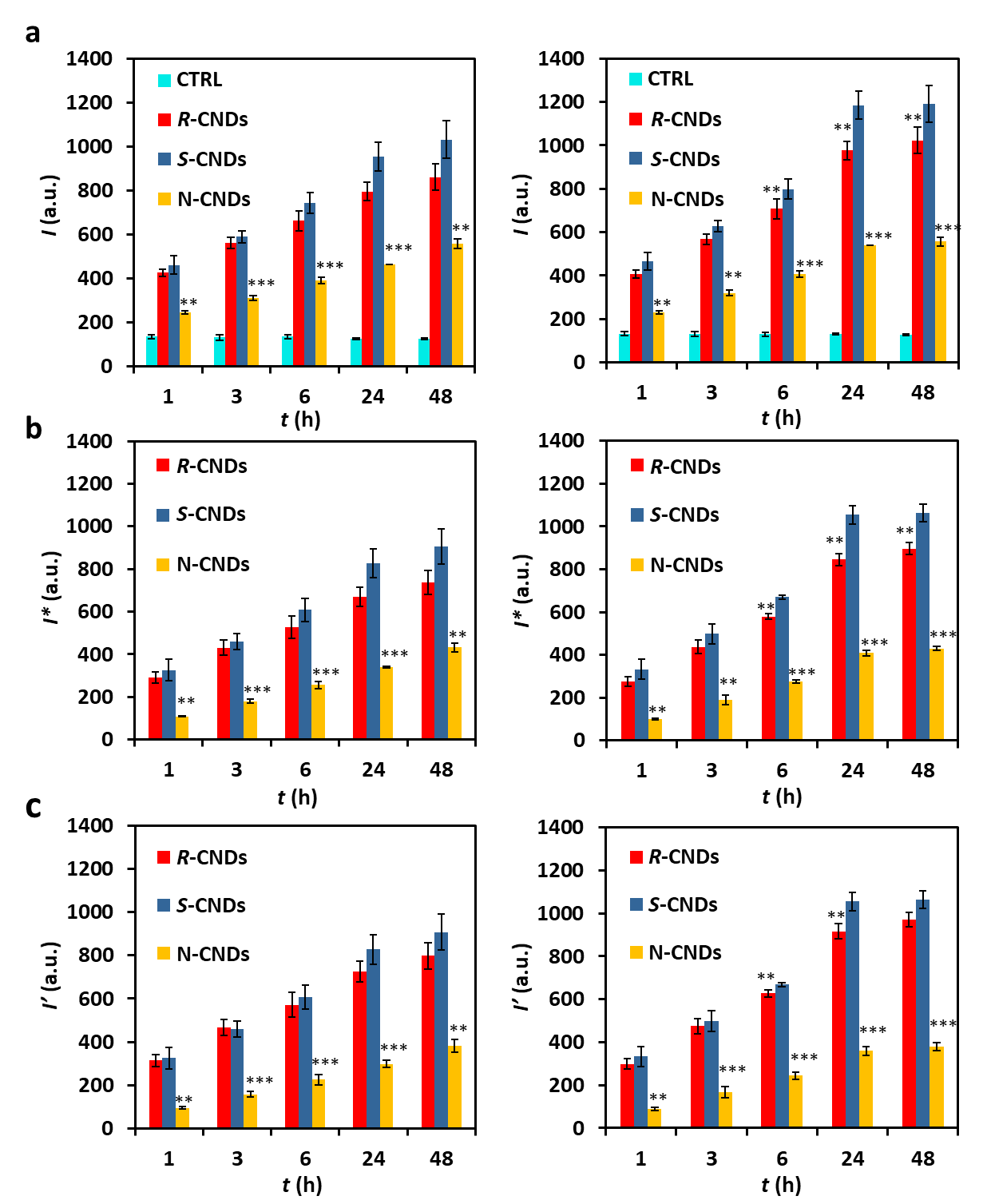
**

**Supplementary Figure 22.** **Mean fluorescence intensity per cell due to internalized *R-*, *S-* and N*-*CNDs as extracted from Supplementary Figure 23**. The left column refers to 10% serum supplement, the right column to serum free conditions. (**a**) Mean fluorescence intensity per cell *I* as detected directly from the flow cytometer data shown in Supplementary Figure 23. (**b**) Mean fluorescence intensity per cell *I*^*^ after background correction. (**c**) Mean fluorescence intensity per cell *I*' after background correction and adjustment for the different fluorescence intensities of each CNDs. Results are shown as mean value ± standard deviation (s.d.) from three independent samples (n = 3) over three independent experiments. *P* values were analyzed by Student’s *t*-test with two-tailed distribution and two-sample equal variance. ***P*<0.01, ****P*<0.001. The resulting data are enlisted in Supplementary Table 6.


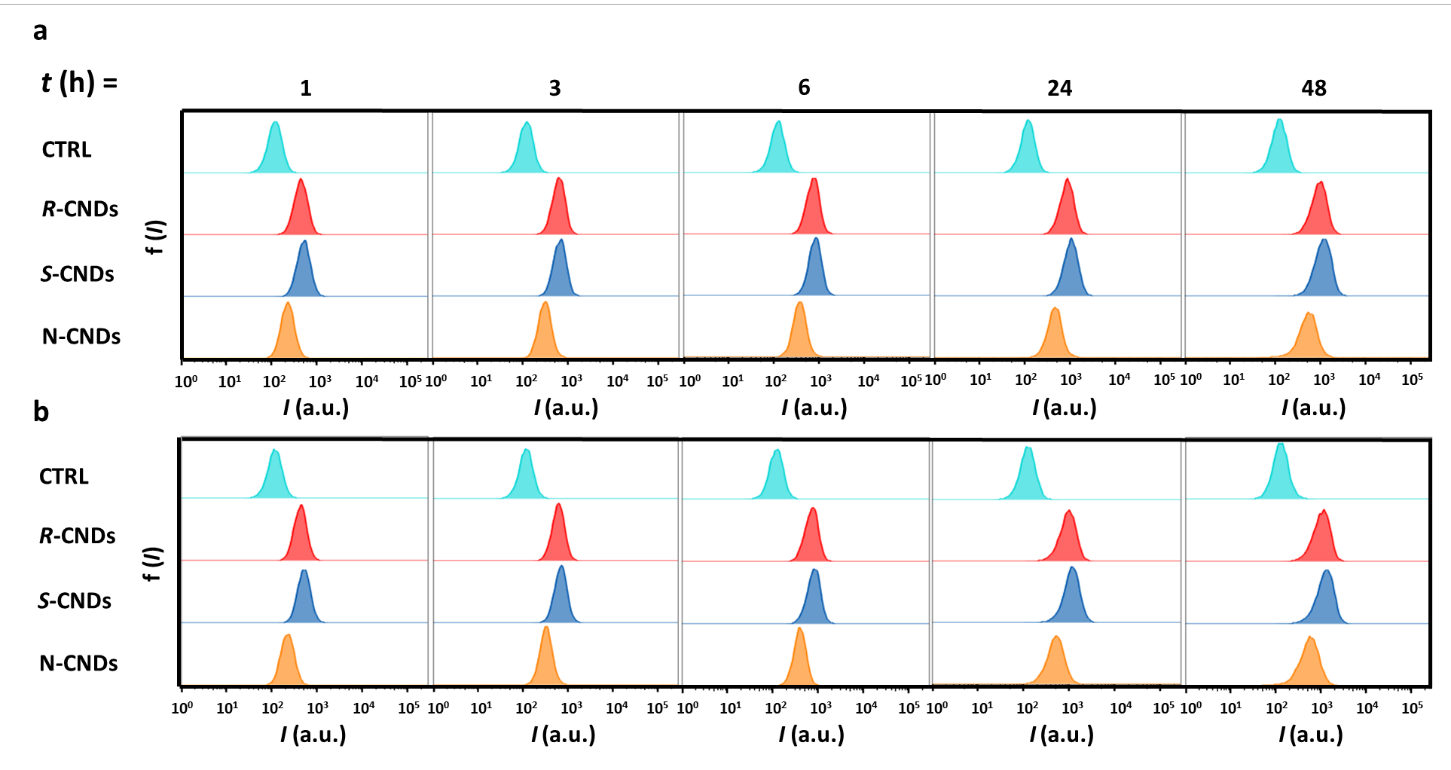


**Supplementary Figure 23.** **CNDs uptake detected by flow cytometry.** Fluorescence distribution f(*I*) per cell of HeLa cells after incubation with *R-*, *S-*, and N*-*CNDs for the time *t* (h) at an exposure concentration of *C*'_CNDs_ = 400 μg mL^–1^ in (**a**) 10% or (**b**) 0% FBS contained DMEM medium measured by flow cytometry. For the control sample (CTRL) no CNDs were added: *C*'_CNDs_ = 0.

**
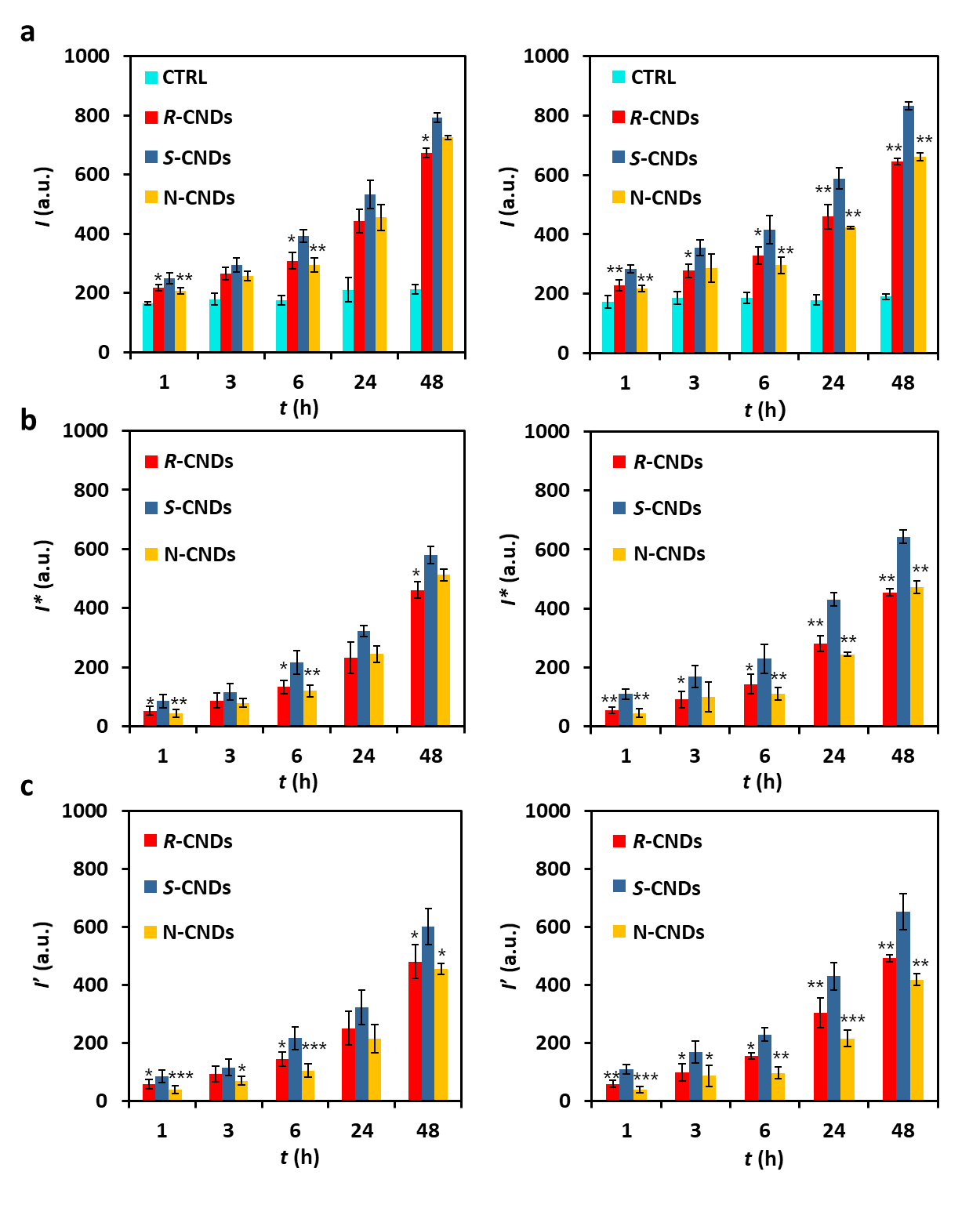
**

**Supplementary Figure 24.** **Mean fluorescence intensity per cell due to internalized *R-*, *S-* and N*-*CNDs as extracted from Supplementary Figure 25**. The left column refers to 10% serum supplement, the right column to serum free conditions. (**a**) Mean fluorescence intensity per cell *I* as detected directly from the flow cytometer data shown in Supplementary Figure 25. (**b**) Mean fluorescence intensity per cell *I*^*^ after background correction. (**c**) Mean fluorescence intensity per cell *I*' after background correction and adjustment for the different fluorescence intensities of each CNDs. Results are shown as mean value ± standard deviation (s.d.) from three independent samples (n = 3) over three independent experiments. *P* values were analyzed by Student’s *t*-test with two-tailed distribution and two-sample equal variance. **P*<0.05, ***P*<0.01, ****P*<0.001. The resulting data are enlisted in Supplementary Table 7.


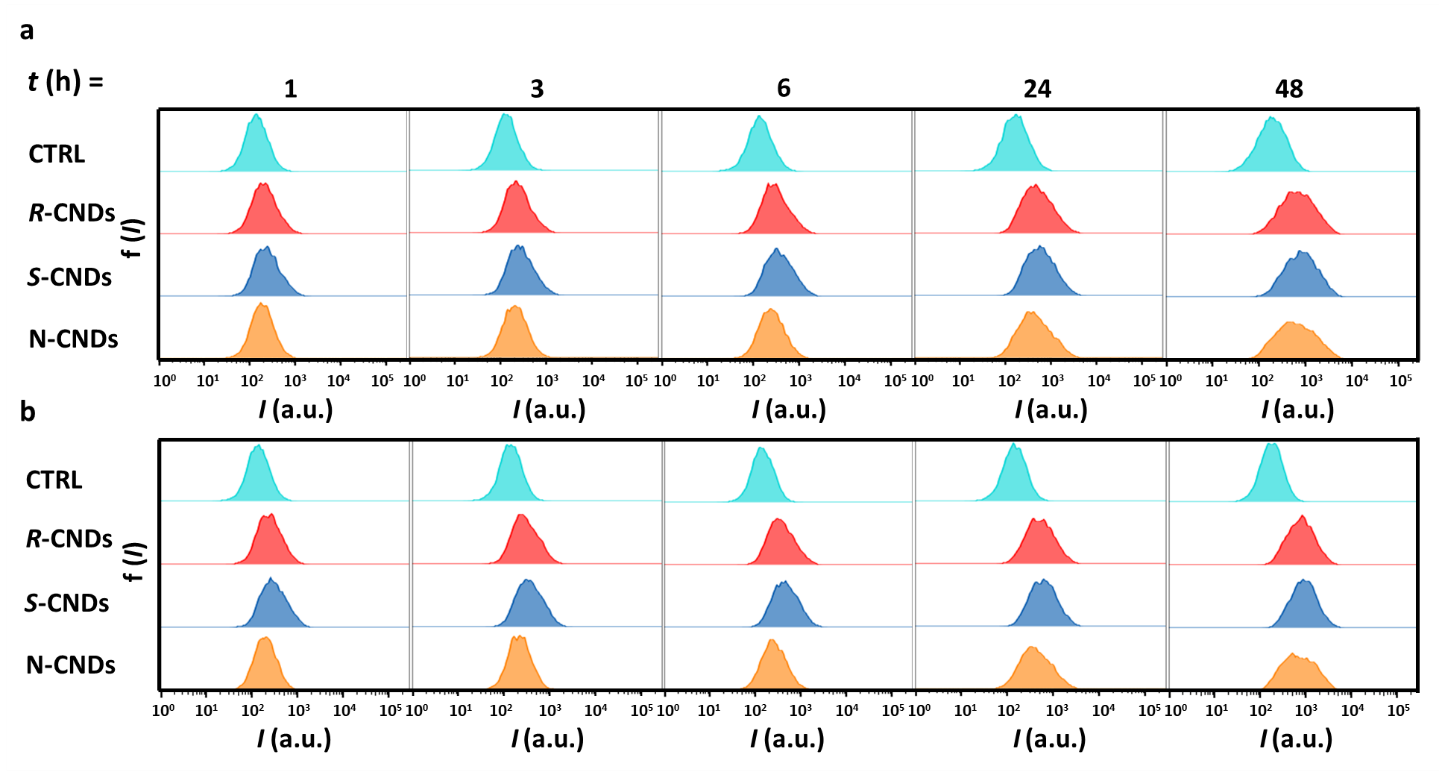


**Supplementary Figure 25.** **CNDs time-dependent uptake detected by flow cytometry.** Fluorescence distribution f(*I*) per cell of THP-1 derived macrophages after incubation with *R*-, *S*-, and N*-*CNDs for the time *t* (h) at an exposure concentration of *C*'_CNDs_ = 400 μg mL^–1^ in (**a**) 10% or (**b**) 0% FBS contained RPMI 1640 medium measured by flow cytometry. For the control sample (CTRL) no CNDs were added: *C*'_CNDs_ = 0.


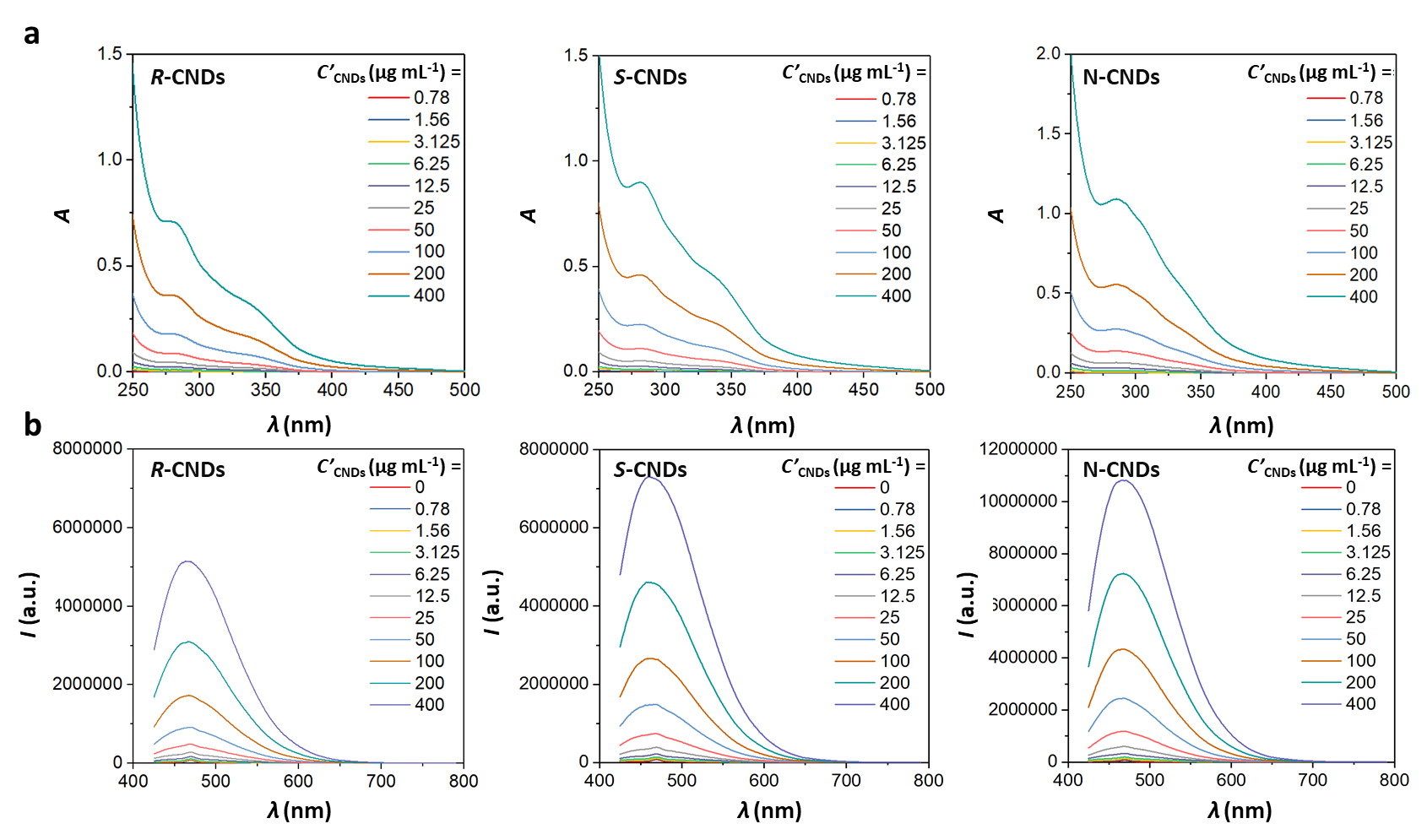


**Supplementary Figure 26. Dose dependent absorbance and fluorescence of *R-*, *S-* and N*-*CNDs at a series of concentrations *C*_CNDs_.** (**a**) UV-Vis absorption and (**b**) fluorescence spectra (*λ*_ex_ = 405 nm) of *R-*, *S-* and N*-*CNDs in Milli-Q water at 298 K. Data were recorded with batch #1.


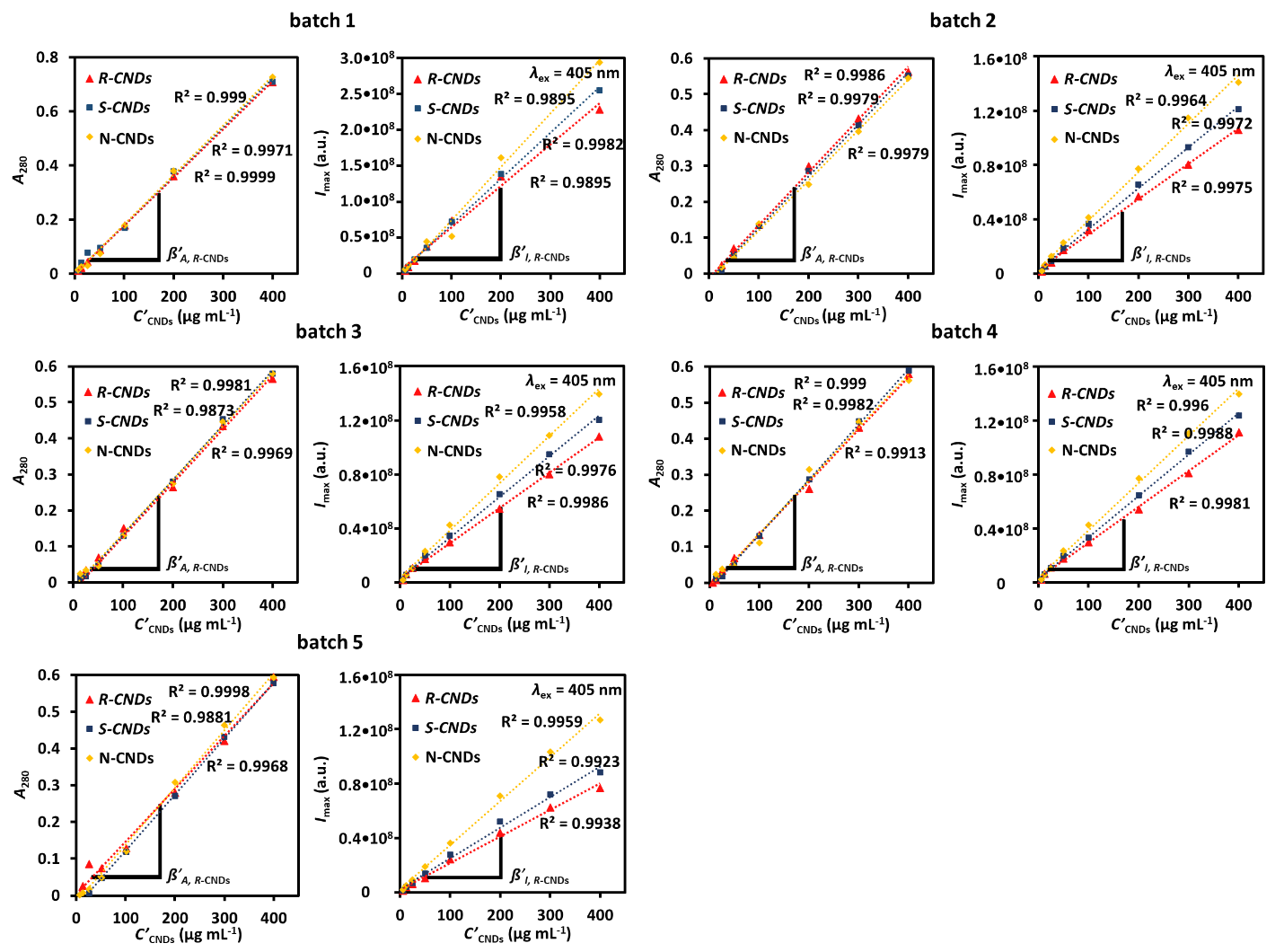


**Supplementary Figure 27.** **Absorption *A*_280_ at 280 nm and integrated fluorescence emission *I*_max_ ranging from 425-475 nm (excitation wavelength *λ*_ex_ = 405 nm) of *R-*, *S-* and N*-*CNDs dissolved in Milli-Q water at the adjusted mass concentration *C*'_CND_.** The *A*_280_(*C*'_CND_) curve was fitted with linear regression to yield the slope *ß*'*_A_*_,j_ (mL μg^–1^) = Δ*A*_280_(j) Δ*C*'_CND_^–1^ (j = *R-*CND, *S-*CND, N*-*CND). The *I*(*C*'_CND_) curve was fitted with linear regression to yield the slope *ß*'*_I_*_,j_ (mL μg^–1^) = Δ*I*_max_(j) Δ*C*'_CND_^–1^ (j = *R-*CND, *S-*CND, N-CND). From these slopes, first the percentual differences Δ*ß*'_i,j_ in the slopes between the *R-*CND and *S-*CND sample to the N*-*CND sample were derived for the absorption and intensity measurements as Δ*ß*'_i,j_ = (*ß*'_i,N_*_-_*_CND_ - *ß*'_i,j_) *ß*'_i,N_*_-_*_CND_^–1^ (i = *A*, *I*; j = *R-*CND, *S-*CND), and then the deviation Δ*ß*'_j_ in these differences between the absorption and intensity measurements were obtained as Δ*ß*'_j_ =|Δ*ß*'*_A_*_,j_ - Δ*ß*'*_I_*_,j_| (j = *R-*CND, *S-*CND). The percentual error in concentration determination was defined as the maximum of these values as Δ*C*'_CND_ *C*'_CND_^–1^ = max(Δ*ß*'*_R-_*_CND_, Δ*ß*'*_S-_*_CND_). The values are enlisted in Supplementary Table 3. Note that in this graph adjusted concentrations *C*'_CND_ are used, in contrast to Figure 1 of the main article, in which the concentrations *C*_CNDs_ as determined by weighting were used. The absorption is measured at 280 nm instead at 405 nm, due to higher extinction coefficients of the CNDs at 280 nm than those at 405 nm.


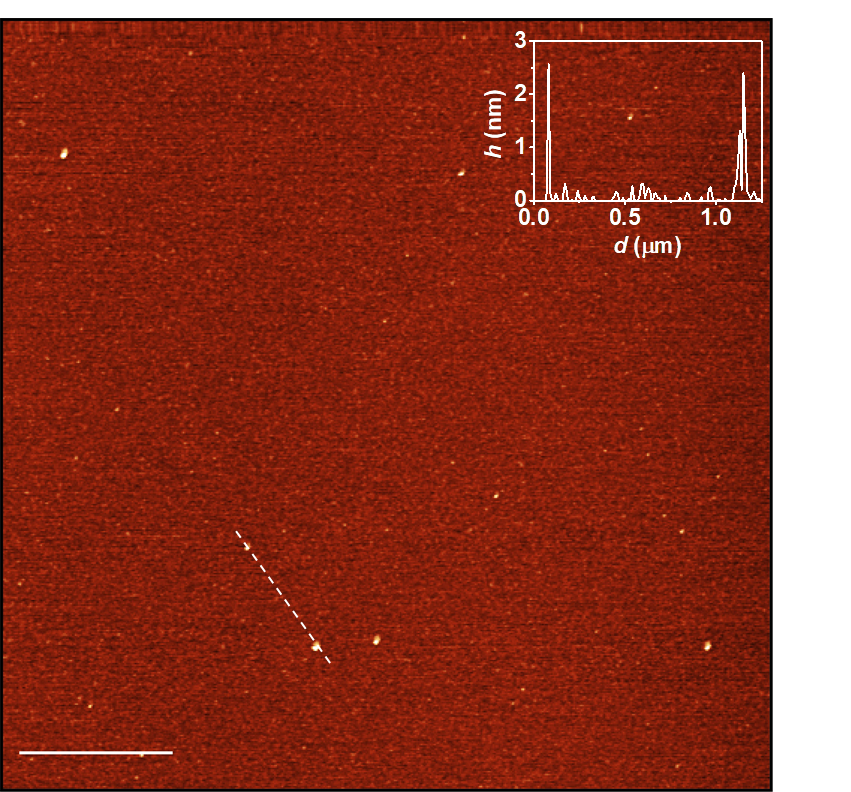


**Supplementary Figure 28.** **AFM of *R-*CNDs.** Tapping mode AFM (5.0 × 5.0 μm) from a drop-casted aqueous solution of *R-*CNDs on a mica substrate (scale bar, 1 μm). The inset is the height profile *h*(*d*) along the dashed line.


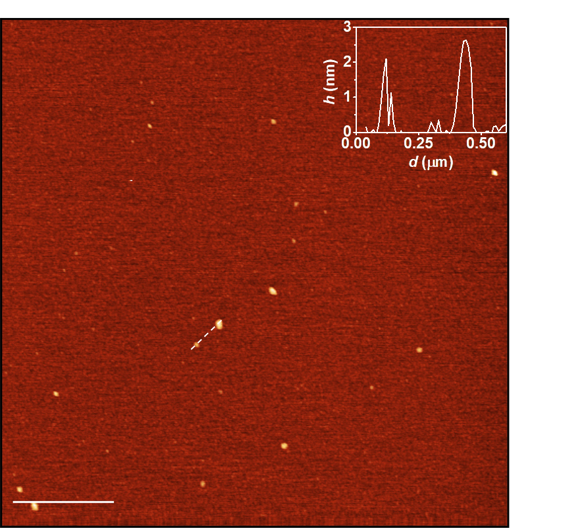


**Supplementary Figure 29.** **AFM of *S-*CNDs.** Tapping mode AFM (5.0 × 5.0 μm) from a drop-casted aqueous solution of *S-*CNDs on a mica substrate (scale bar, 1 μm). The inset is the height profile *h*(*d*) along the dashed line.

**Supplementary Figure 30.** **TEM of *R-*CNDs.** TEM images from dried drop-casted aqueous solution of *R-*CNDs on TEM grids. The experiment was conducted for 1 time (n = 1) and representative images are shown.

**Supplementary Figure 31.** **TEM of *S-*CNDs.** TEM images from dried drop-casted aqueous solution of *S-*CNDs on TEM grids. The experiment was conducted for 1 time (n = 1) and representative images are shown.


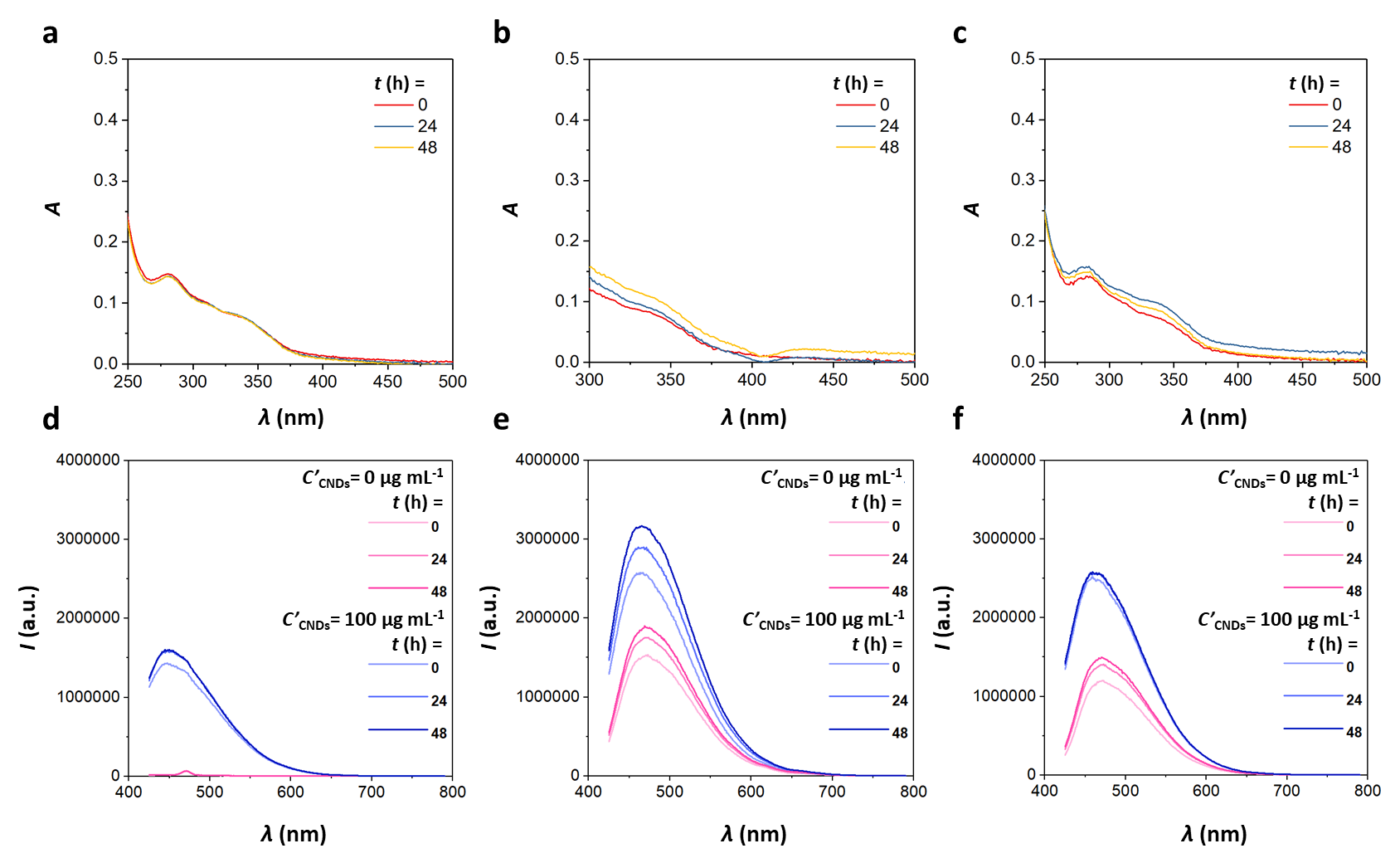


**Supplementary Figure 32. CNDs time- and concentration-dependent UV-Vis and fluorescence spectra.** UV-Vis absorption and fluorescence spectra (*λ*_ex_ = 405 nm) of *S-*CNDs after incubation for 0, 24 or 48 h with (**a**,**d**) H_2_O, (**b**,**e**) RPMI 1640 medium without phenol red containing 10% FBS, and (**c**,**f**) RPMI 1640 medium without phenol red without serum supplement measured. Data were recorded with batch #1. In the fluorescence spectra (**d**,**e**,**f**) the pink curves are the blanks (no CNDs) which show the autofluorescence of the medium and the blue curves originate from the *S*-CNDs dissolved in water or medium.


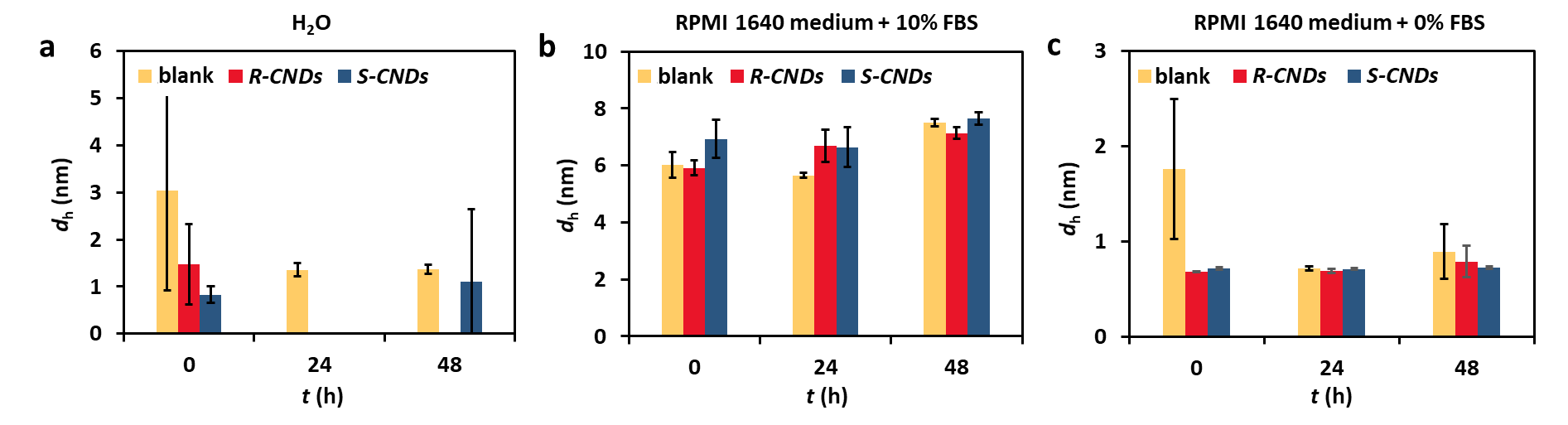


**Supplementary Figure 33. Hydrodynamic diameter *d*_h_ as derived from the number distribution from DLS measurements, after incubation of *R-*, *S-*CNDs in different media for the incubation time *t***. The data for the yellow bars were recorded in plain medium (*i.e.* water, medium with and without serum supplement). The red and blue bars correspond to samples where *R-* and *S-*CNDs had been added to the media, respectively. Data were recorded with batch #1. Results are shown as mean values with error bars (i.e., the corresponding standard deviations) from three independent samples (n = 3) over three independent experiments.


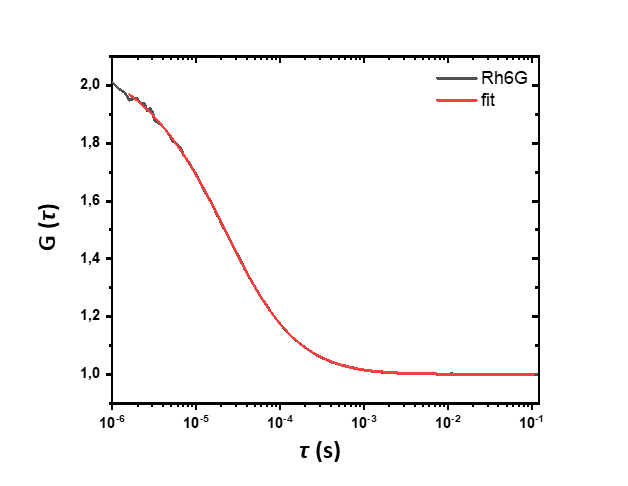


**Supplementary Figure 34.** **FCS of Rhodamine 6G.** Autocorrelation function *G(τ)* (grey line) and fit (red line) as obtained with FCS on Rhodamine 6G in Milli-Q water.


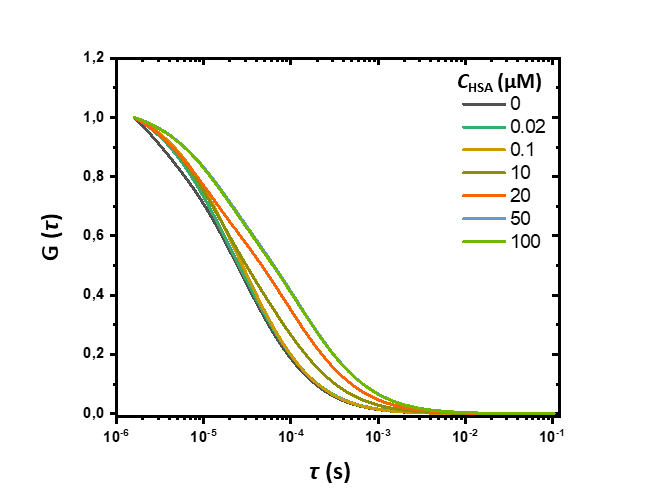


**Supplementary Figure 35.** **FCS of S-CNDs in presence of HSA.** Autocorrelation functions *G(τ)* and fit as determined with FCS measurements on *S-*CNDs (batch #1) incubated with different HSA concentrations *C*_HSA_ in PBS (legend is the *C*_HSA_ in µM). The parameters as determined from the fits of each curve are enlisted in Supplementary Table 11.


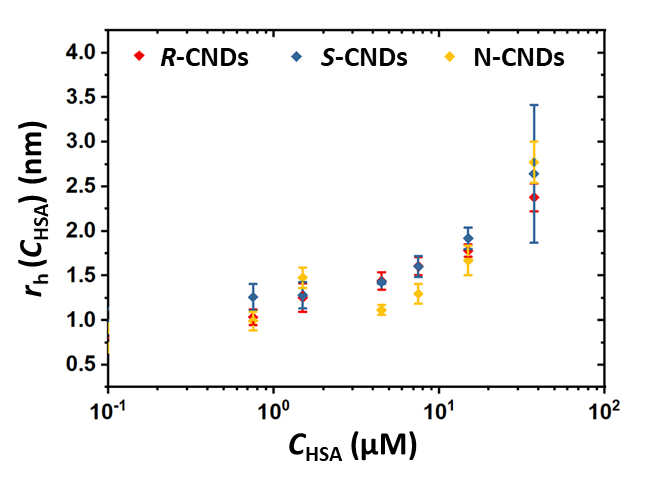


**Supplementary Figure 36. Hydrodynamic radius of CNDs.** Change of hydrodynamic radius *r*_h_ of CNDs (batch #1) in Milli-Q water and in the presence of human serum albumin (HSA) in dependence of the protein concentrations *C*_HSA_. Results are shown as mean values with error bars (i.e., the corresponding standard deviations) from three independent samples (n = 3) over three independent experiments.


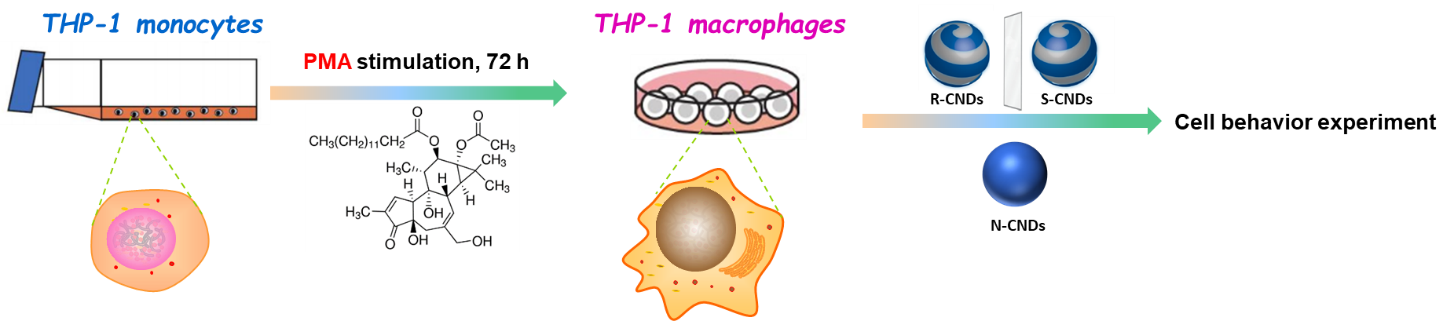


**Supplementary Figure 37.** **Schematic representation of THP-1 cell cultures preparation for CNDs exposure experiments.** THP-1 monocytes were differentiated to THP-1 derived macrophages, by stimulation with PMA, for CNDs application.

**
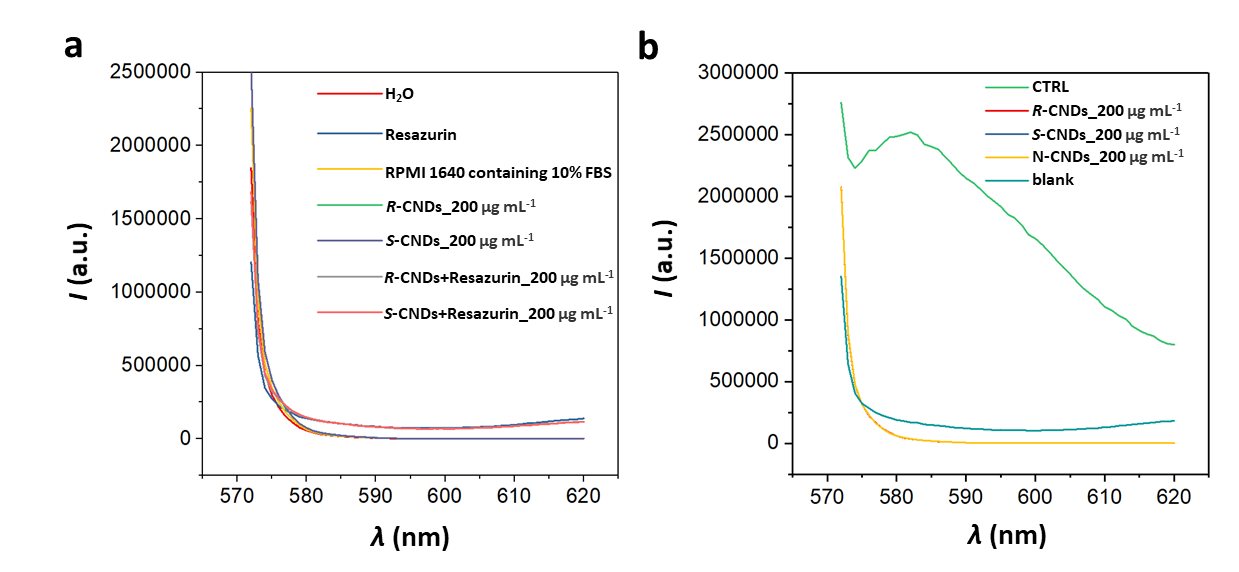
**

**Supplementary Figure 38.** **Fluorescence emission analysis of resazurin and THP-1.** (**a**) Fluorescence intensity *I* of resazurin after mixed with CNDs (no cells involved). (**b**) Fluorescence intensity *I* of THP-1 derived macrophages which have been either exposed to CNDs (*R-*, *S-*, N*-*CNDs) or to resazurin (control). Blanks were recorded on wells without seeded cells. Only the resazurin treated cells provide fluorescence, the intrinsic CND fluorescence in this spectral range is negligible.


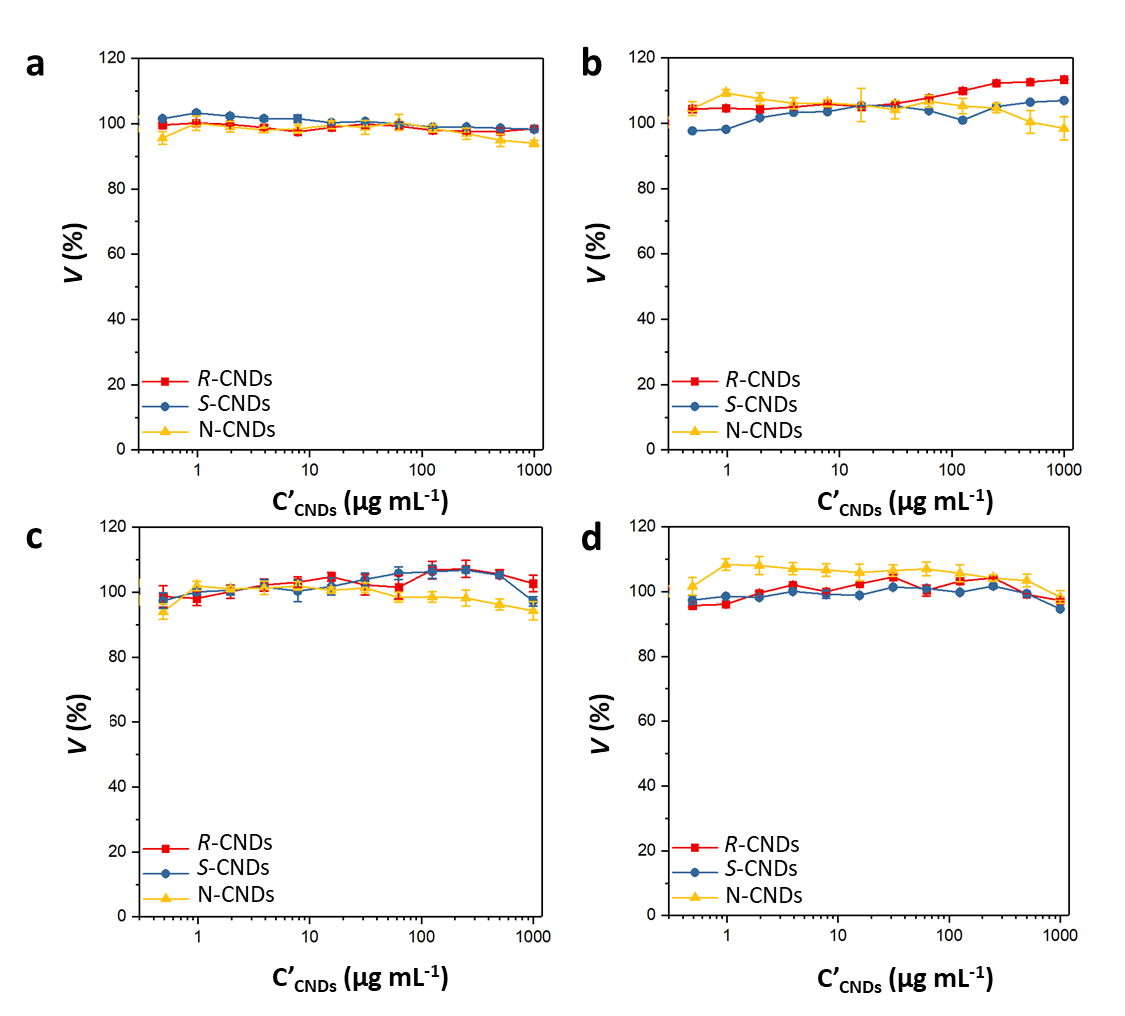


**Supplementary Figure 39.** **Cell viability V of THP-1-derived macrophages after exposure to CNDs at different concentrations *C*'_CND_**. V was measured by the resazurin assay after (**a**,**b**) 24 or (**c**,**d**) 48 h of CNDs exposure. In (**a, c**) the RPMI 1640 medium was supplemented with 10% FBS, or in (**b,d**) was without serum supplement. The viability V represents the fluorescence intensity of cells having been treated with CNDs after normalizing to the fluorescence intensity of untreated control cells. Results are showed as percent cell viability V (%) (mean) ± standard deviation (s.d.) from three independent experiments (n = 3).

**
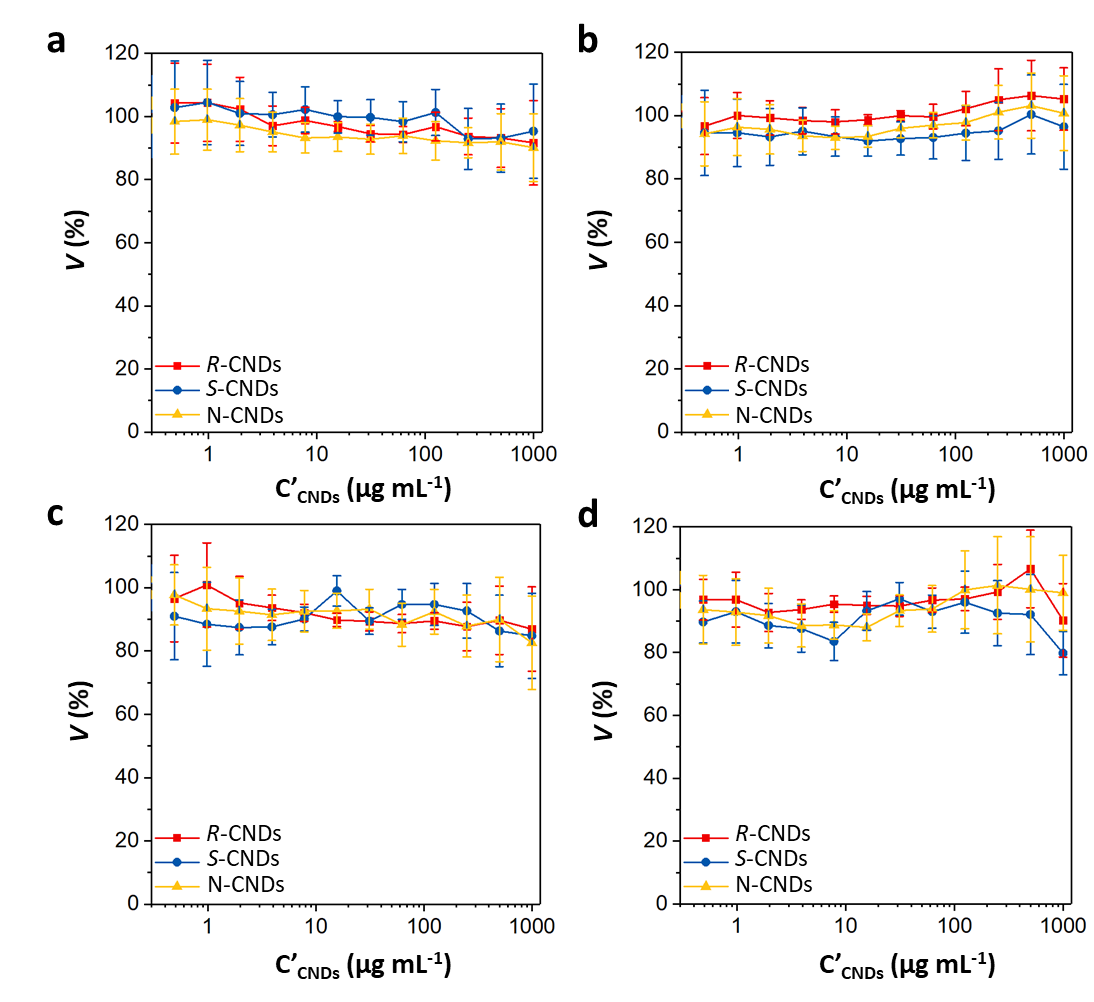
**

**Supplementary Figure 40.** **Cell viability V of HeLa cells after exposure to CNDs at different concentrations *C*'_CND_**. V was measured by the resazurin assay after (**a**,**b**) 24 or (**c**,**d**) 48 h of CNDs exposure. In (**a**, **c**) the DMEM medium was supplemented with 10% FBS, or in (**b**,**d**) was without serum supplement. The viability V represent the fluorescence intensity of cells having been treated with CNDs after normalizing to the fluorescence intensity of untreated control cells. Results are showed as percent cell viability V (%) (mean) ± standard deviation (s.d.) from three independent experiments (n = 3).


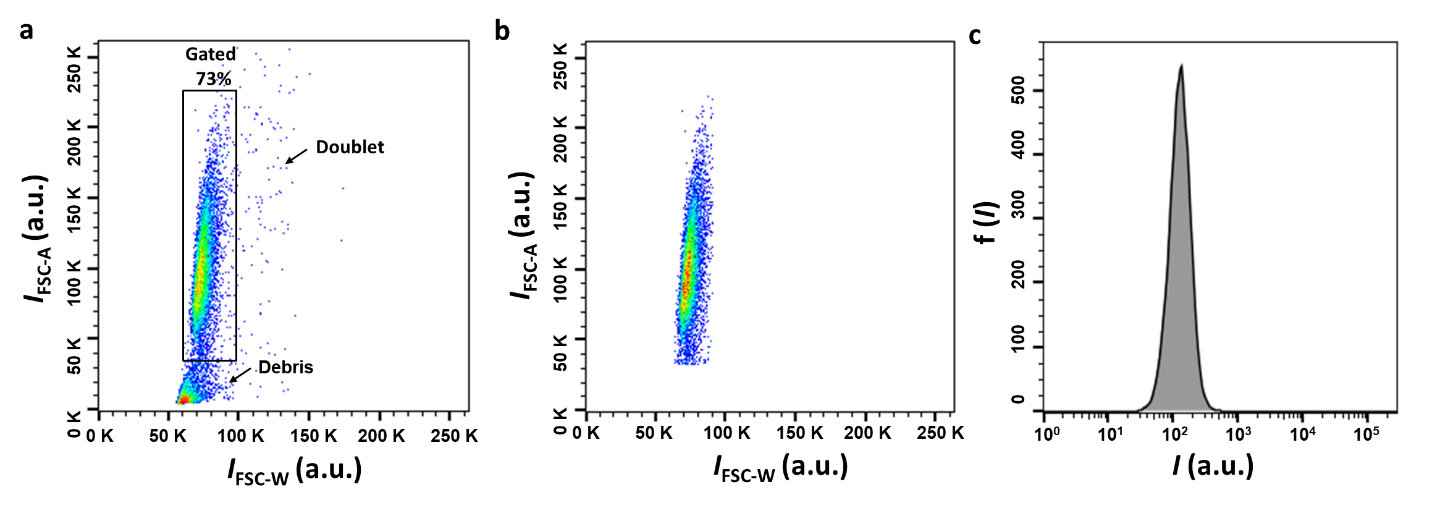


**Supplementary Figure 41.** **Gating strategy to distinguish cells with internalized CNDs from autofluorescent cells**. (**a**) Density plot of the forward scattering area intensity *I*_FSC-A_ versus the forward scattering width intensity *I*_FSC-W,_ as well as the gate to identify single cells. (**b**) Density plot of the gated single cells displayed as forward scattering area intensity *I*_FSC-A_ versus forward scattering width intensity *I*_FSC-W._ (**c**) Fluorescence distribution f(*I*) of the CNDs fluorescence per cell as measured by flow cytometry for HeLa cells.


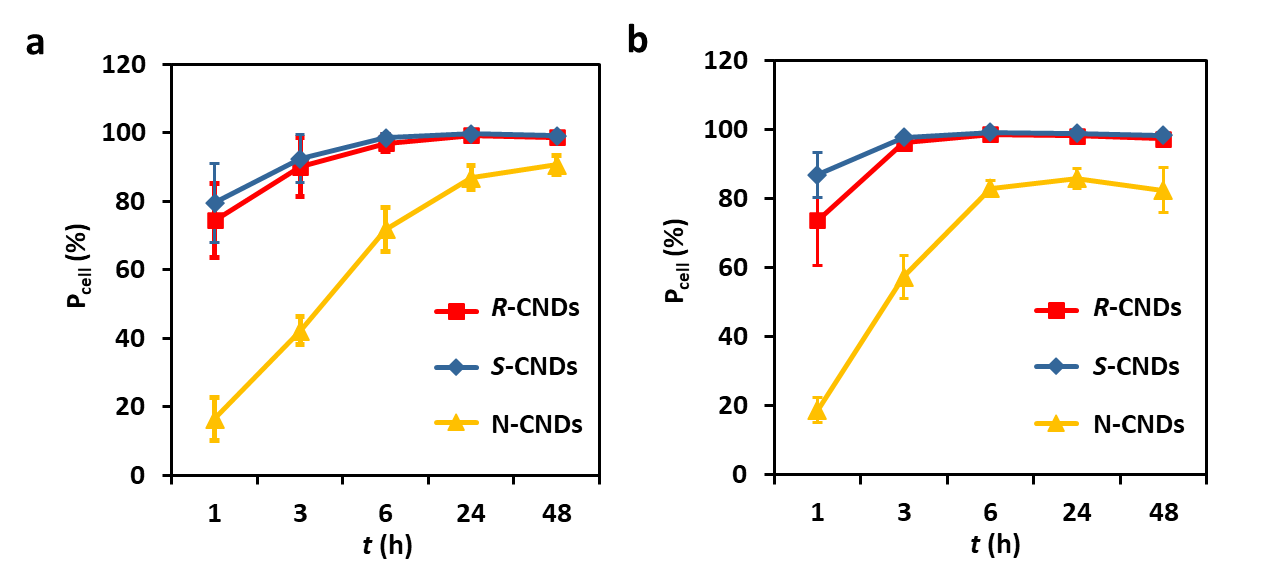


**Supplementary Figure 42.** **Percentage of HeLa cells P_cell_ which had incorporated CNDs**. P_cell_ above the threshold level of autofluorescence were measured after an exposure time *t* to CNDs at a concentration of *C*'_CNDs_ = 400 μg mL^–1^ in DMEM medium (**a**) with and (**b**) without serum supplement. Data are plotted as mean values ± standard deviations (s.d.) from three independent experiments (n = 3). Data are based on the measurements shown in Supplementary Figure 23.


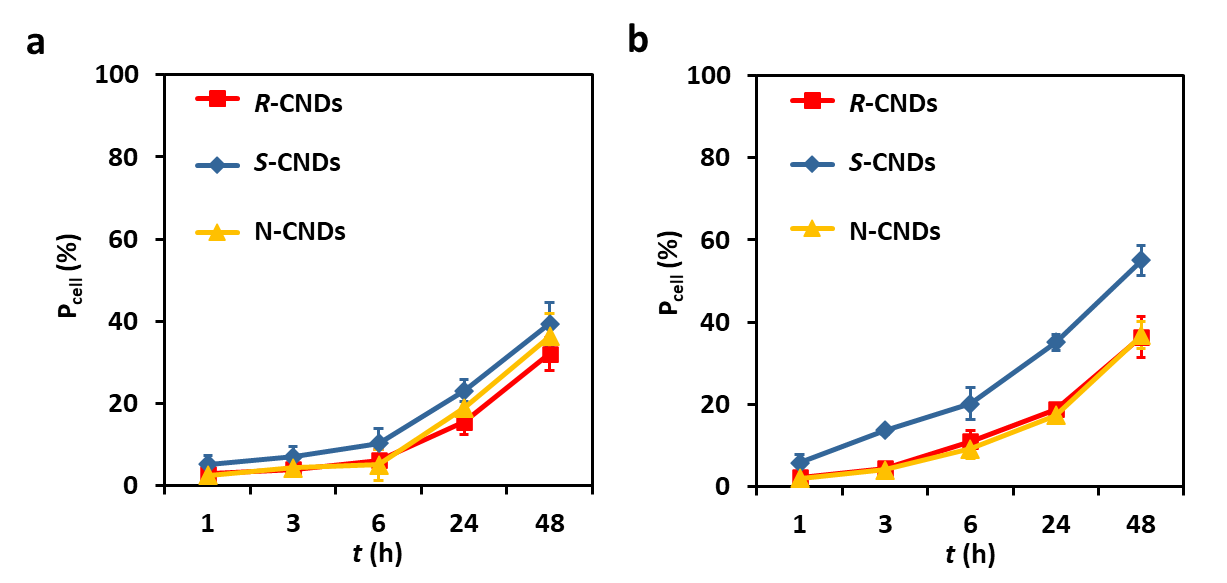


**Supplementary Figure 43.** **Percentage of THP-1 cells P_cell_ which had incorporated CNDs.** P_cell_ above the threshold level of autofluorescence were measured after an exposure time *t* to CNDs at a concentration of *C*'_CNDs_ = 400 μg mL^–1^ in RPMI medium (**a**) with and (**b**) without serum supplement. Data are plotted as mean values ± standard deviations (s.d.) from three independent experiments (n = 3). Data are based on the measurements shown in Supplementary Figure 25.

**
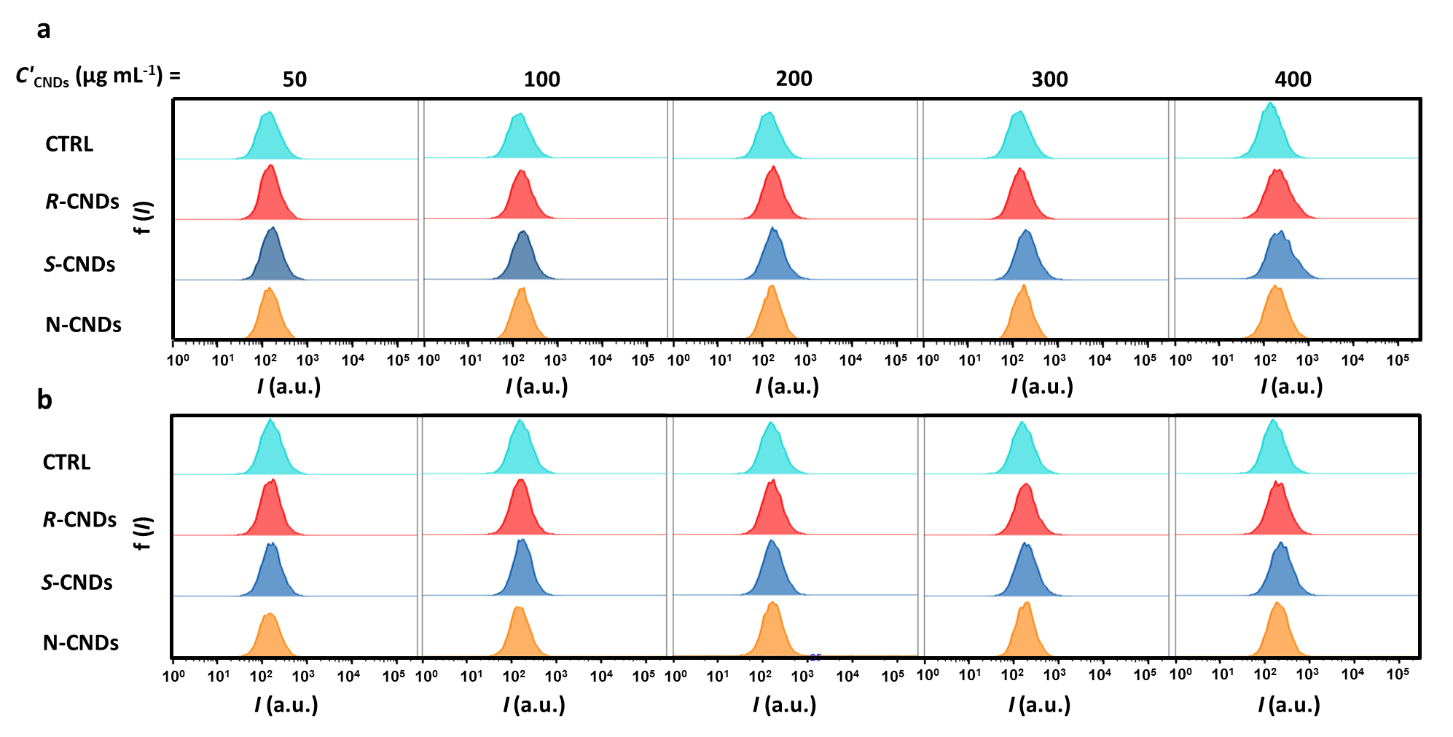
**

**Supplementary Figure 44.** **CNDs concentration-dependent uptake detected by flow cytometry**. Fluorescence distribution f(*I*) per cell of THP-1 derived macrophages after incubation with *R*-, *S*-, and N*-*CNDs for the time *t* = 1 h at different exposure concentrations *C*'_CNDs_ in (**a**) 10% or (**b**) 0% FBS contained RPMI 1640 medium measured by flow cytometry. For the control sample (CTRL) no CNDs were added: *C*'_CNDs_ = 0.

**
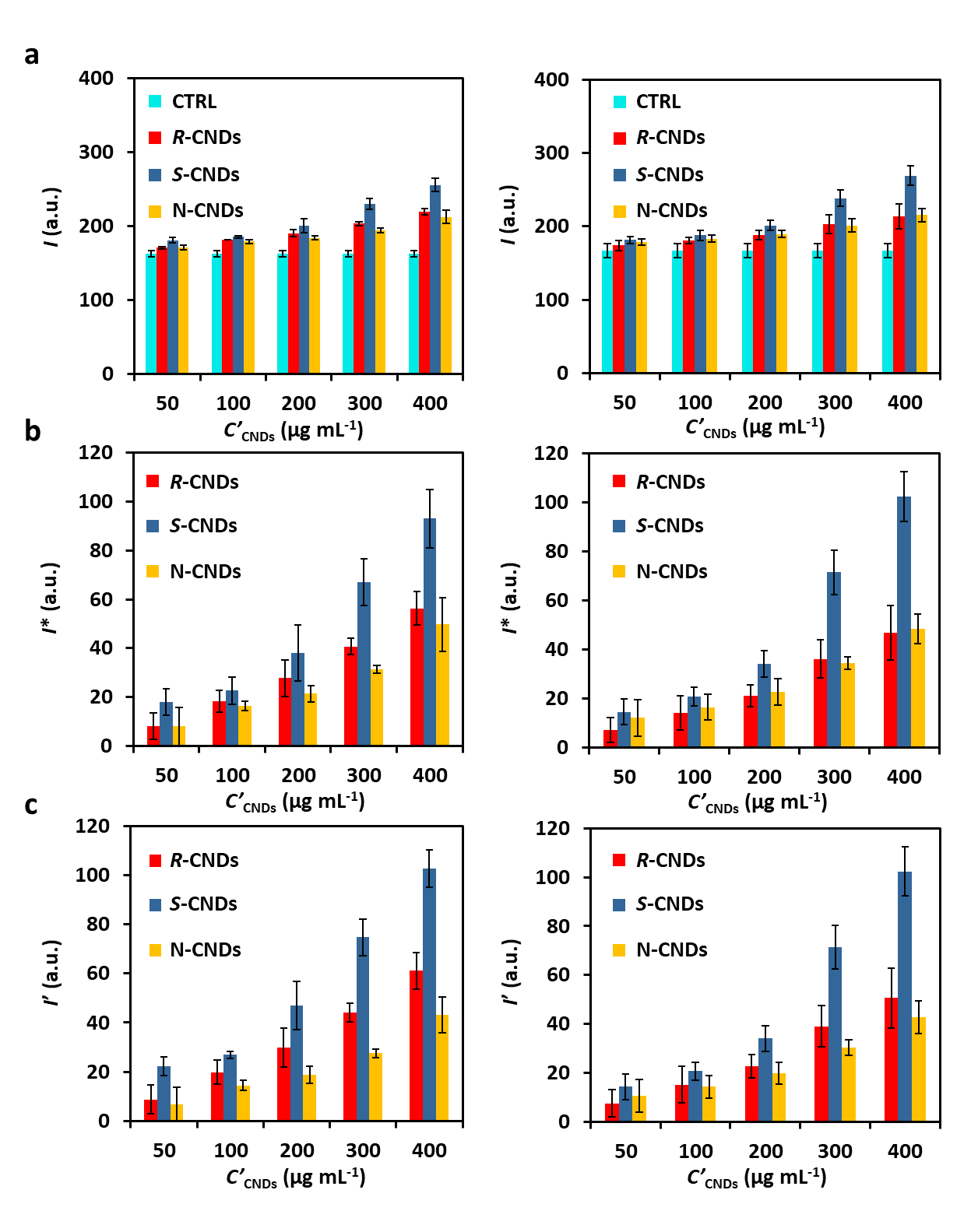
**

**Supplementary Figure 45.** **Mean fluorescence intensity per cell due to internalized *R-*, *S-* and N*-*CNDs as extracted from Supplementary Figure 44**. The left column refers to 10% serum supplement, the right column to serum free conditions. (**a**) Mean fluorescence intensity per cell *I* as detected directly from the flow cytometer data shown in Supplementary Figure 44. (**b**) Mean fluorescence intensity per cell *I*^*^ after background correction. (**c**) Mean fluorescence intensity per cell *I*' after background correction and adjustment for the different fluorescence intensities of each CNDs. Results are shown as mean value ± standard deviation (s.d.) from three independent samples (n = 3) over three independent experiments. The resulting data are enlisted in Supplementary Table 12.


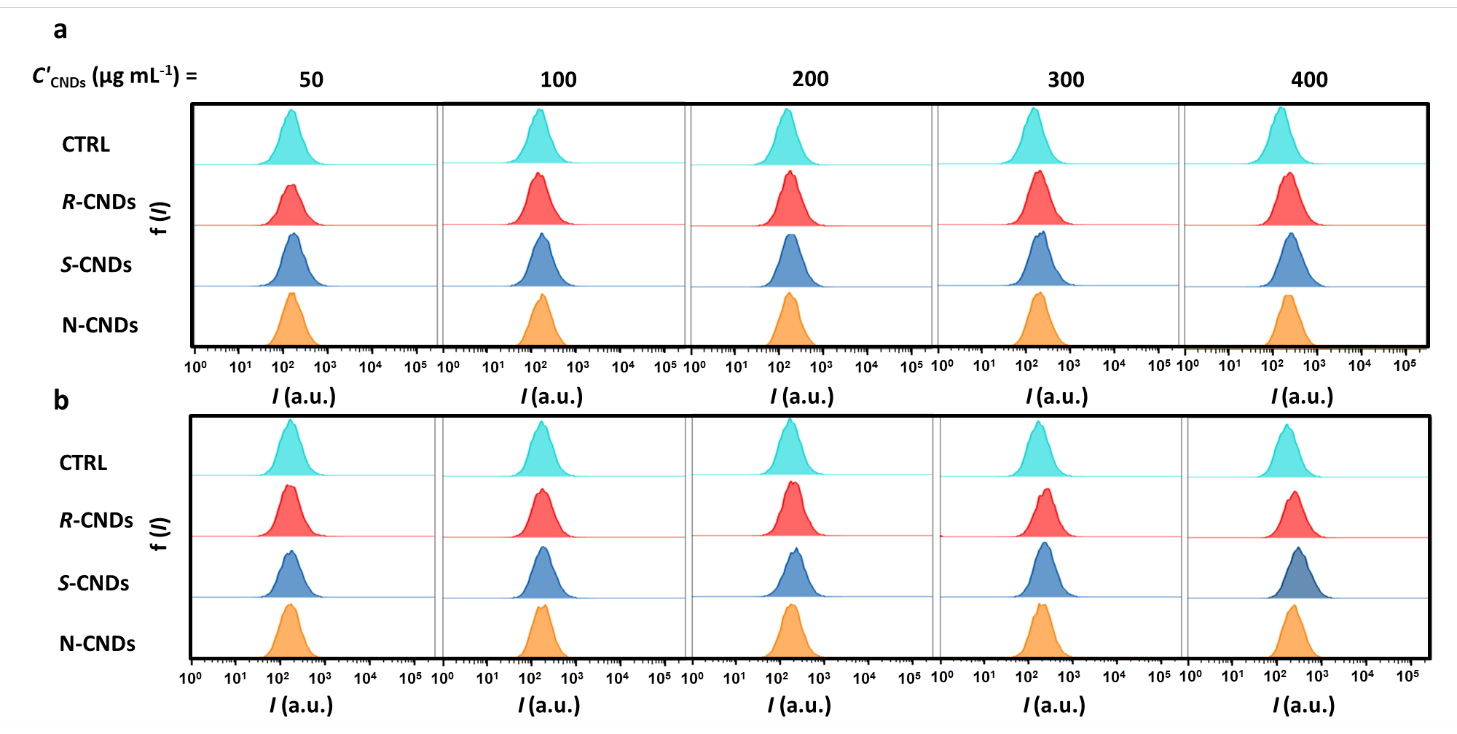


**Supplementary Figure 46.**  **CNDs concentration-dependent uptake detected by flow cytometry.** Fluorescence distribution f(*I*) per cell of THP-1 derived macrophages after incubation with *R*-, *S*-, and N*-*CNDs for the time *t* = 3 h at different exposure concentrations *C*'_CNDs_ in (**a**) 10% or (**b**) 0% FBS contained RPMI 1640 medium measured by flow cytometry. For the control sample (CTRL) no CNDs were added: *C*'_CNDs_ = 0.

**
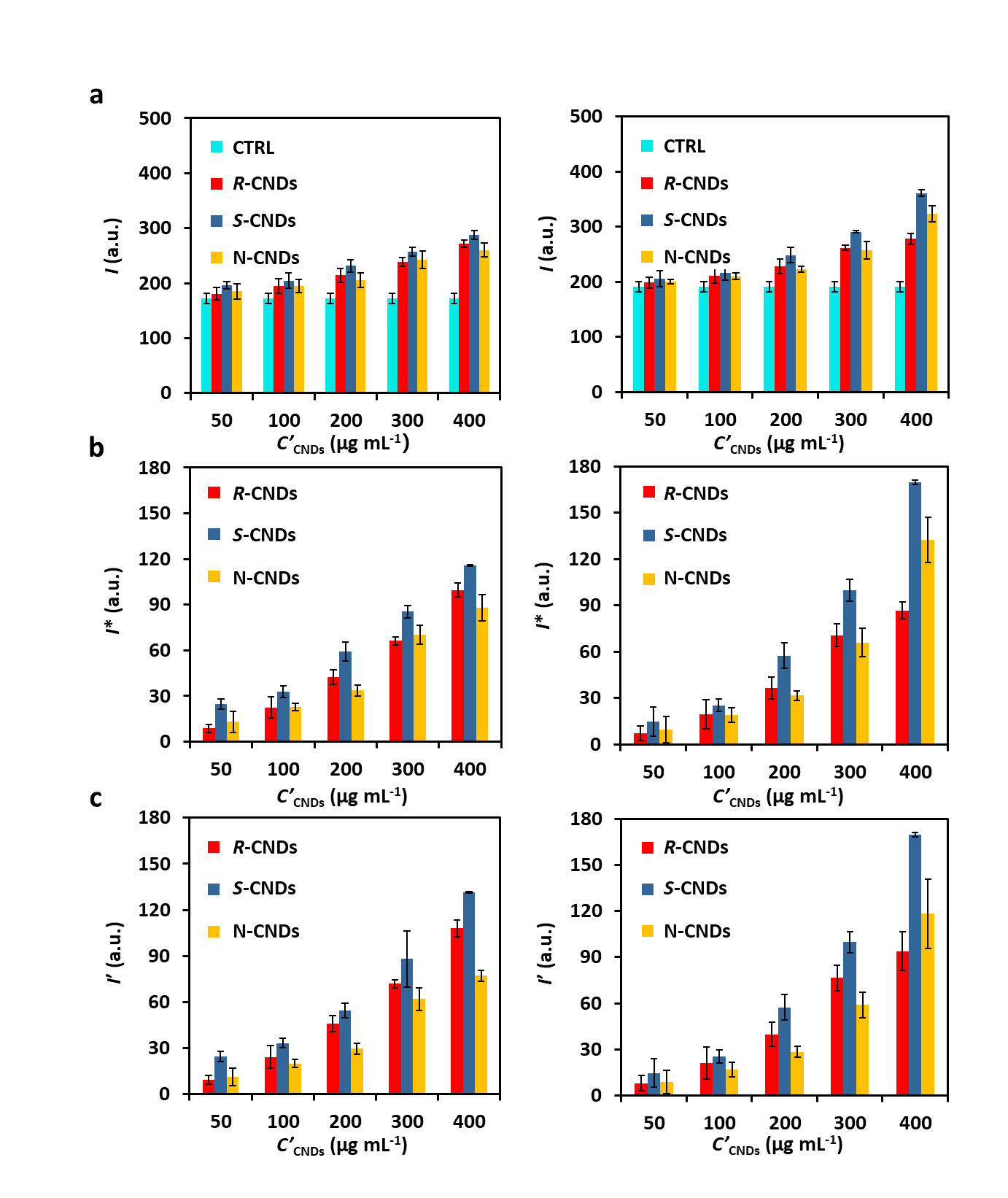
**

**Supplementary Figure 47.** **Mean fluorescence intensity per cell due to internalized CNDs as extracted from Supplementary Figure 46**. The left column refers to 10% serum supplement, the right column to serum free conditions. (**a**) Mean fluorescence intensity per cell *I* as detected directly from the flow cytometer data shown in Supplementary Figure 46. (**b**) Mean fluorescence intensity per cell *I*^*^ after background correction. (**c**) Mean fluorescence intensity per cell *I*' after background correction and adjustment for the different fluorescence intensities of the different types of CNDs. Results are shown as mean value ± standard deviation (s.d.) from three independent samples (n = 3) over three independent experiments. The resulting data are enlisted in Supplementary Table 13.

**
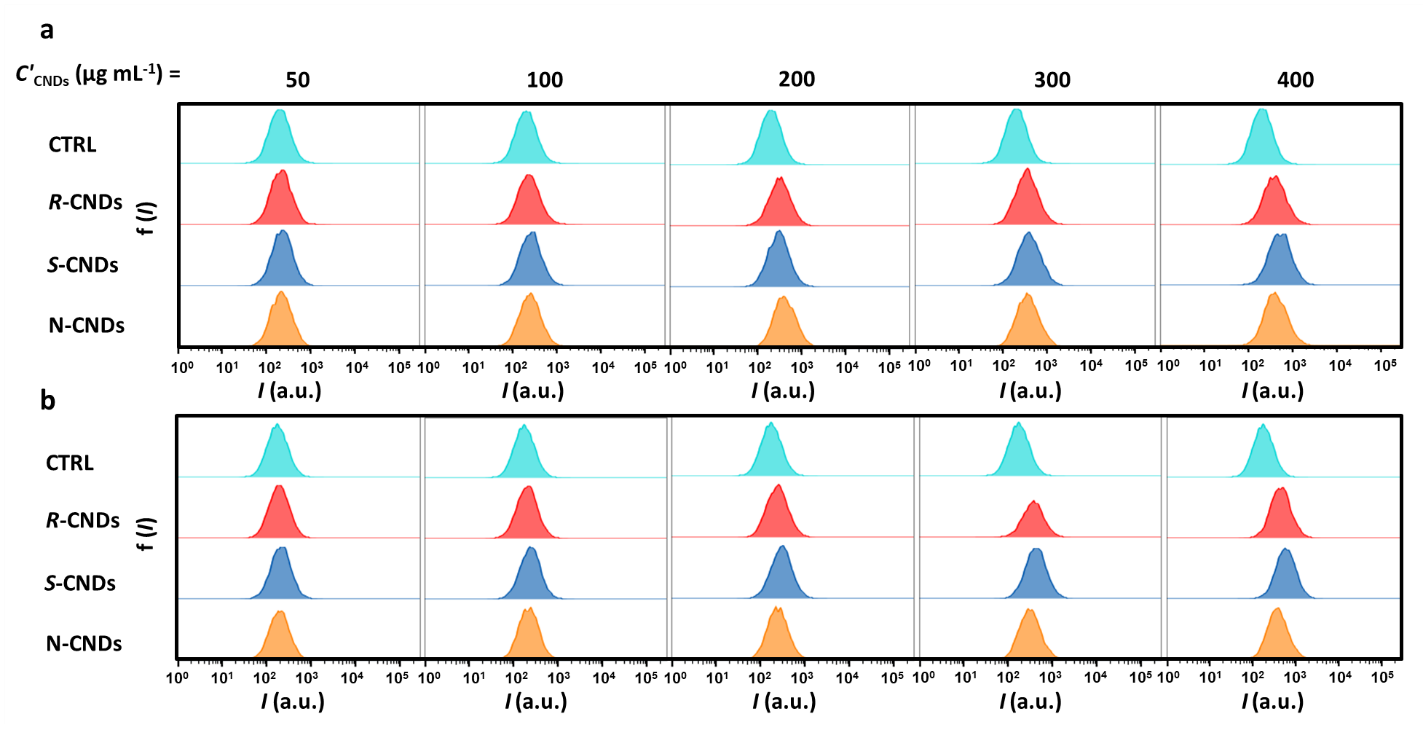
**

**Supplementary Figure 48.** **CNDs concentration-dependent uptake detected by flow cytometry.** Fluorescence distribution f(*I*) per cell of THP-1 derived macrophages after incubation with *R*-, *S*-, and N*-*CNDs for the time *t* = 24 h at different exposure concentrations *C*'_CNDs_ in (**a**) 10% or (**b**) 0% FBS contained RPMI 1640 medium measured by flow cytometry. For the control sample (CTRL) no CNDs were added: *C*'_CNDs_ = 0.


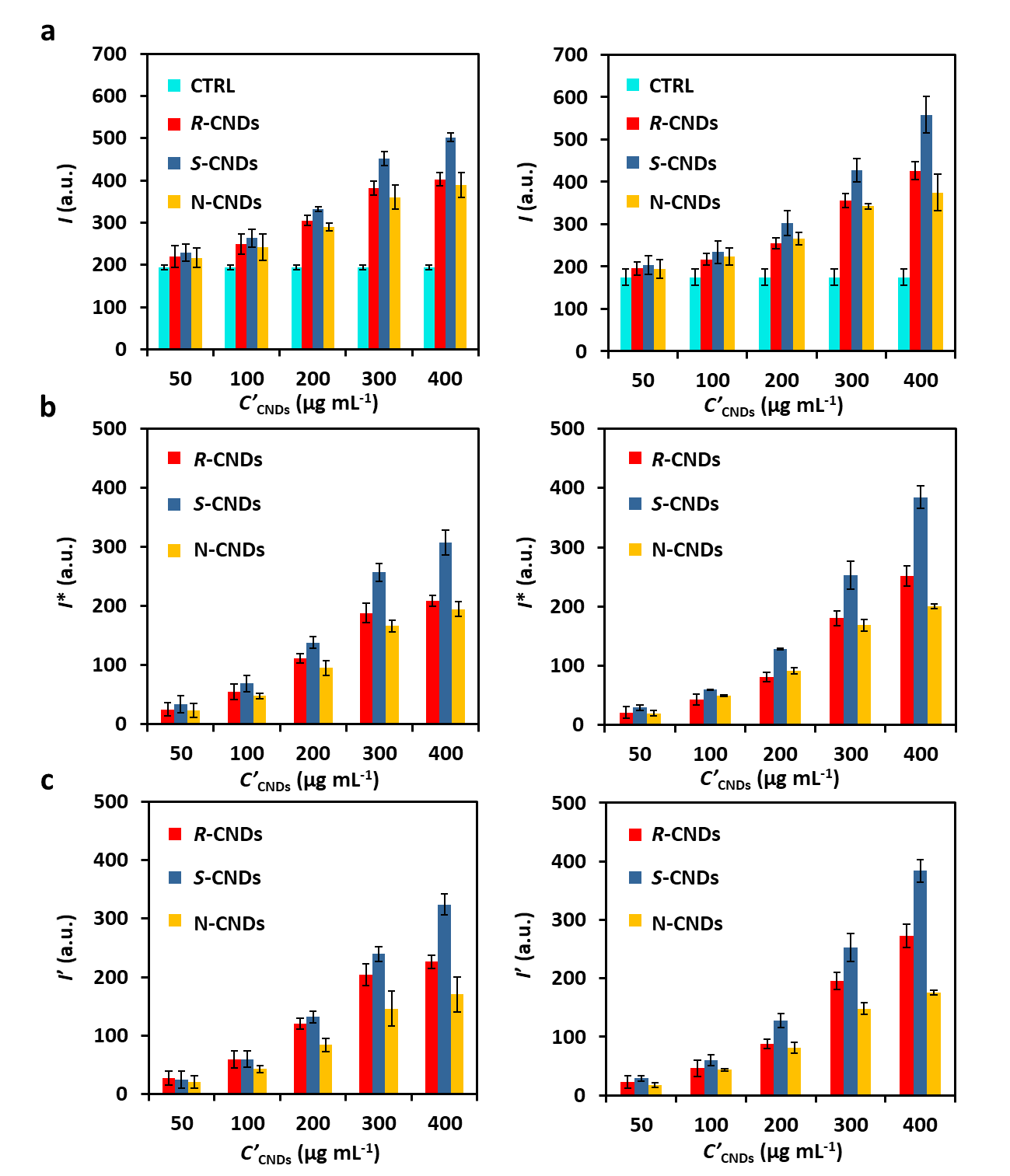


**Supplementary Figure 49.** **Mean fluorescence intensity per cell due to internalized CNDs as extracted from Supplementary Figure 48.** The left column refers to 10% serum supplement, the right column to serum free conditions. (**a**) Mean fluorescence intensity per cell *I* as detected directly from the flow cytometer data shown in Supplementary Figure 48. (**b**) Mean fluorescence intensity per cell *I*^*^ after background correction. (**c**) Mean fluorescence intensity per cell *I*' after background correction and adjustment for the different fluorescence intensities of the different types of CNDs. Results are shown as mean value ± standard deviation (s.d.) from three independent samples (n = 3) over three independent experiments. The resulting data are enlisted in Supplementary Table 14.


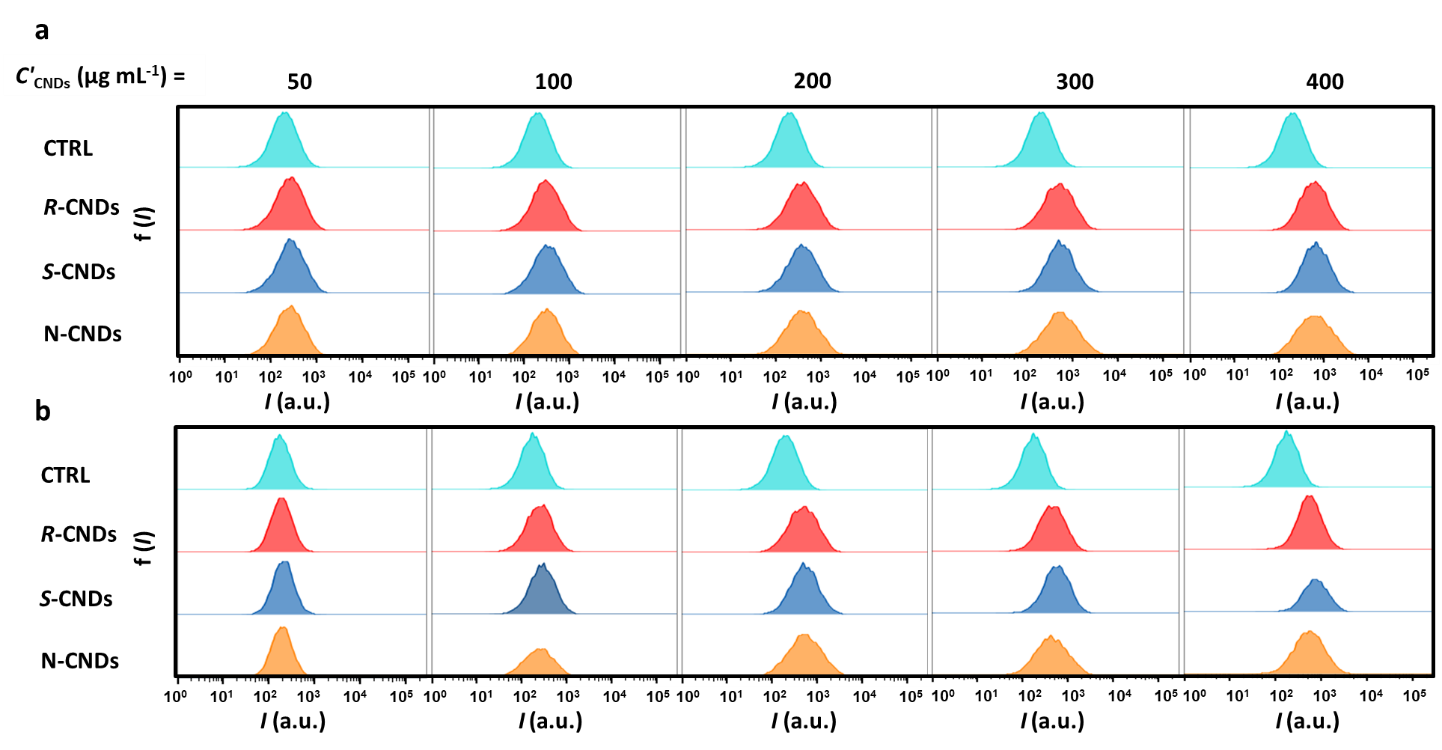


**Supplementary Figure 50.** **CNDs concentration-dependent uptake detected by flow cytometry.** Fluorescence distribution f(*I*) per cell of THP-1 derived macrophages after incubation with *R*-, *S*-, and N*-*CNDs for the time *t* = 48 h at different exposure concentrations *C*'_CNDs_ in (**a**) 10% or (**b**) 0% FBS contained RPMI 1640 medium measured by flow cytometry. For the control sample (CTRL) no CNDs were added: *C*'_CNDs_ = 0.


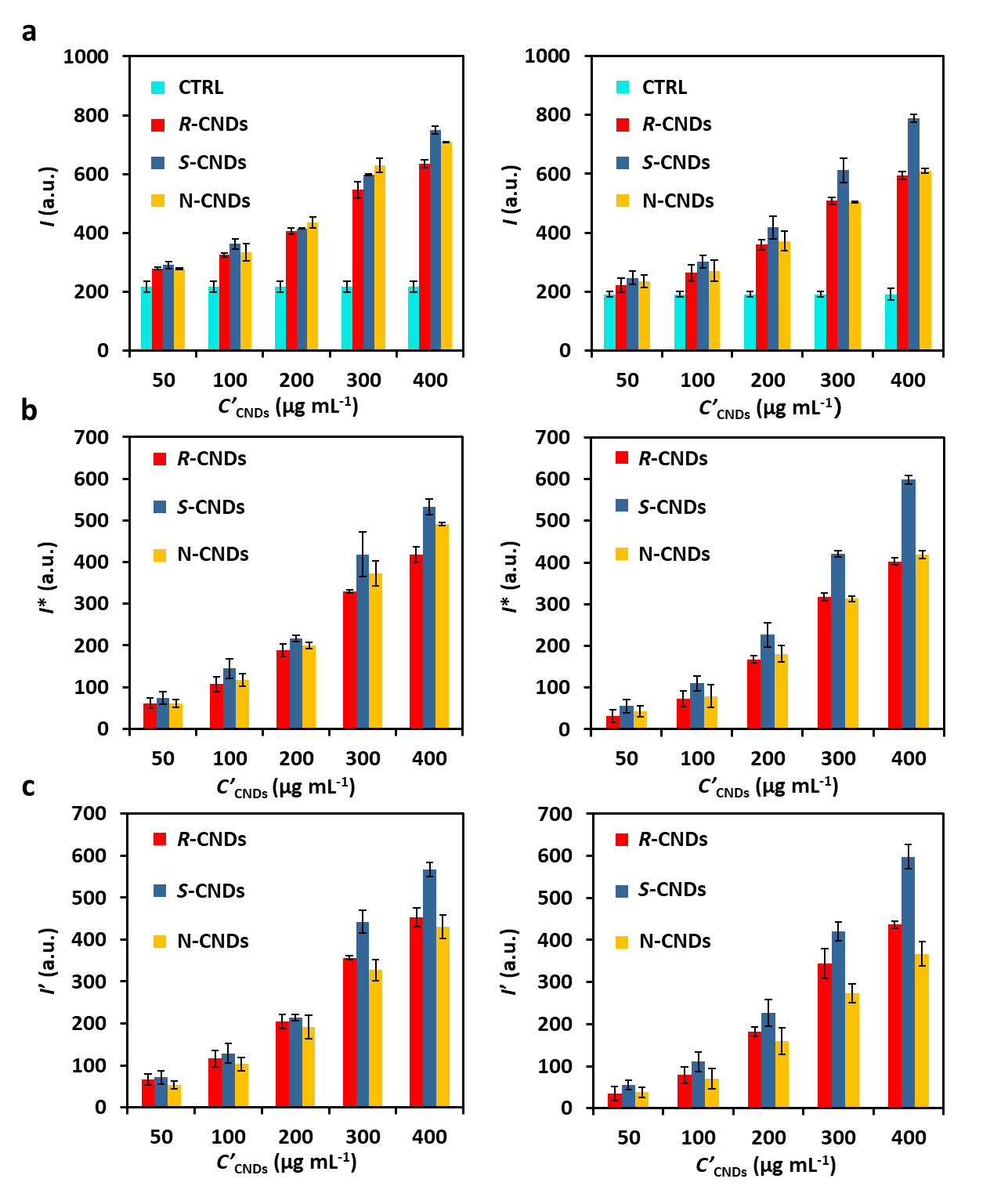


**Supplementary Figure 51.** **Mean fluorescence intensity per cell due to internalized CNDs as extracted from Supplementary Figure 50**. The left column refers to 10% serum supplement, the right column to serum free conditions. (**a**) Mean fluorescence intensity per cell *I* as detected directly from the flow cytometer data shown in Supplementary Figure 50. (**b**) Mean fluorescence intensity per cell *I*^*^ after background correction. (**c**) Mean fluorescence intensity per cell *I*' after background correction and adjustment for the different fluorescence intensities of the different types of CNDs. Results are shown as mean value ± standard deviation (s.d.) from three independent samples (n = 3) over three independent experiments. The resulting data are enlisted in Supplementary Table 15.


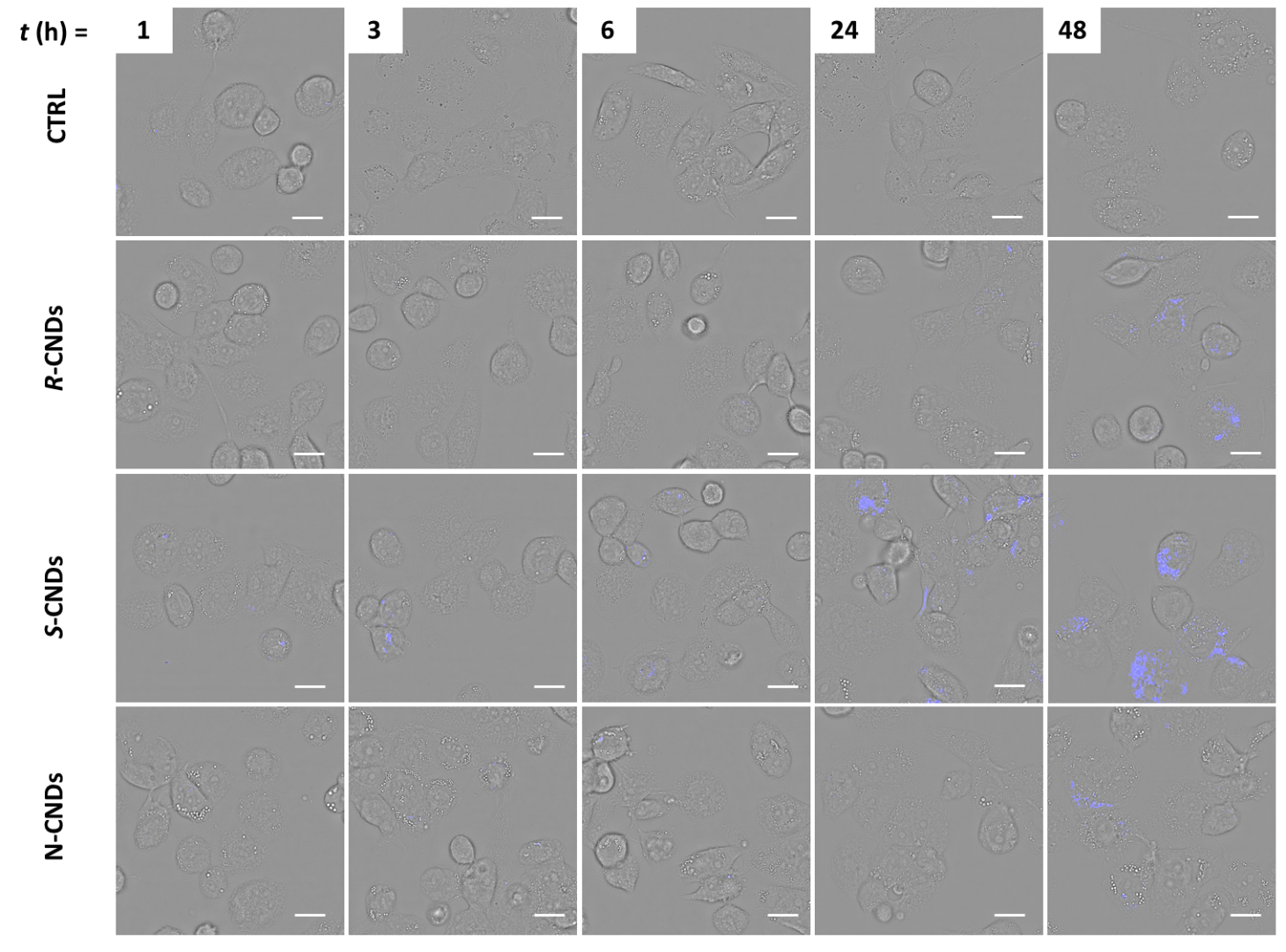


**Supplementary Figure 52.** **Representative CLSM images reflecting the cellular uptake of *R-*, *S-*, or N*-*CNDs by THP-1 derived macrophages**. Images were recorded after CNDs incubation at concentration of *C*'_CNDs_ = 400 μg mL^–1^ for the time *t* in 10% FBS containing RPMI 1640 medium. For the control (CTRL) no CNDs had been added (*C*'_CNDs_ = 0). The scale bar is 20 μm. An overlay of the brightfield and the fluorescence channel is shown. The experiments were repeated independently for three times (n = 3) and representative images are shown.


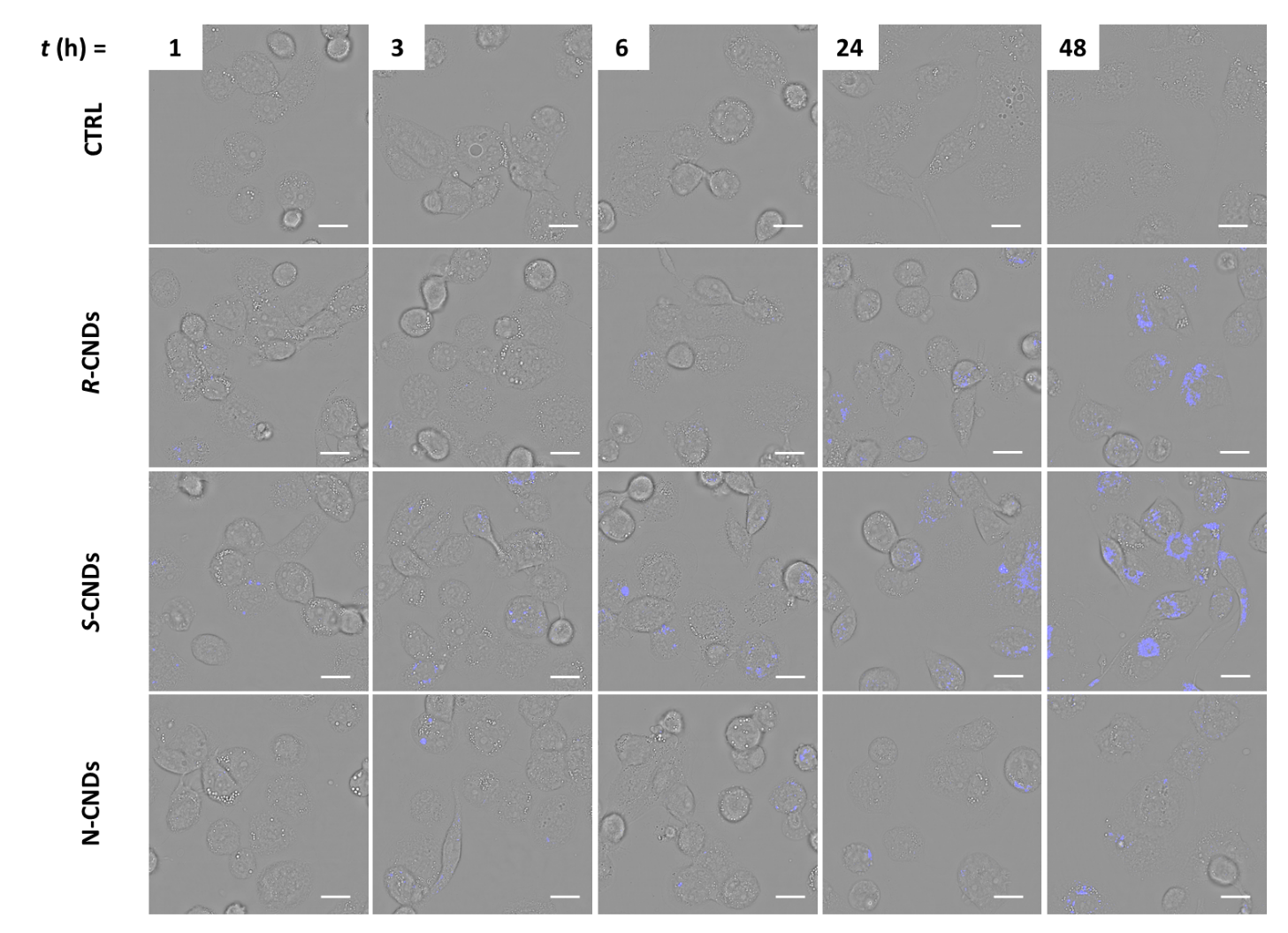


**Supplementary Figure 53.** **Representative CLSM images reflecting the cellular uptake of *R-*, *S-*, or N*-*CNDs by THP-1 derived macrophages**. Images were recorded after incubation at concentration of *C*'_CNDs_ = 400 μg mL^–1^ for the time *t* in serum-free RPMI 1640 medium. For the control (CTRL) no CNDs had been added (*C*'_CNDs_ = 0). The scale bar is 20 μm. An overlay of the brightfield and the fluorescence channel is shown. The experiments were repeated independently for three times (n = 3) and representative images are shown.

**
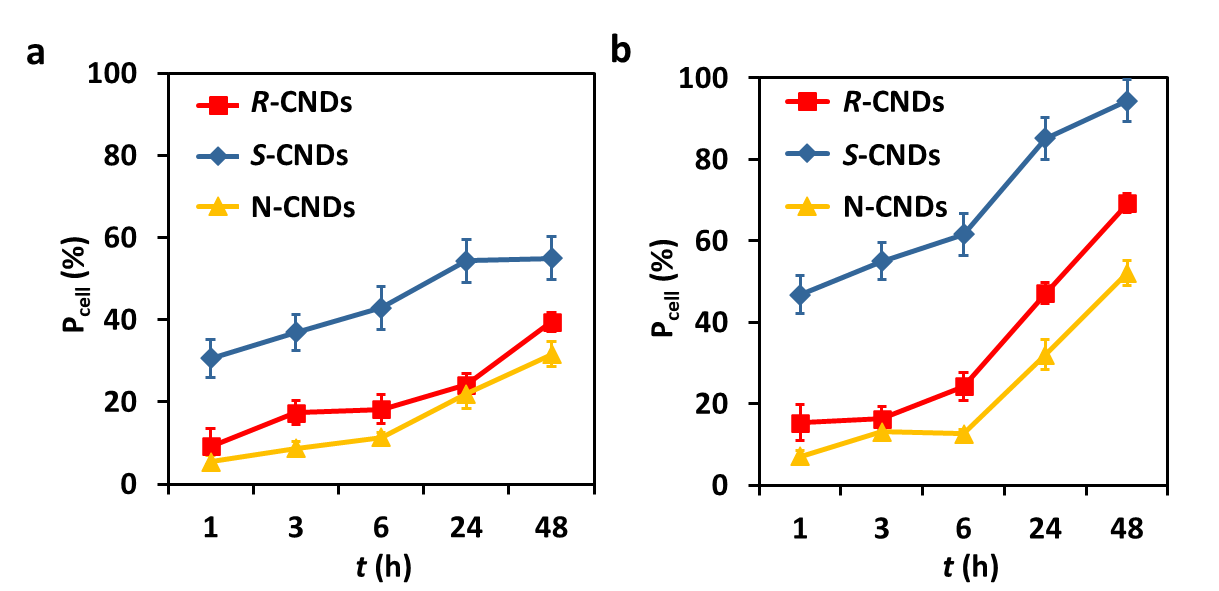
**

**Supplementary Figure 54.** **Percentage P_cell_ of THP-1-derived macrophages which had incorporated CNDs.** P_cell_ was calculated at different exposure times of CNDs at a concentration of *C*'_CND_ = 400 μg mL^–1^ in RPMI 1640 medium supplemented with (**a**) 10% or (**b**) 0% serum. n ≥ 200 cells in at least 20 images from 3 independent experiments were analyzed for each time point. Results are shown as mean value ± standard deviation (s.d.) from three independent samples (n = 3) over three independent experiments.

**
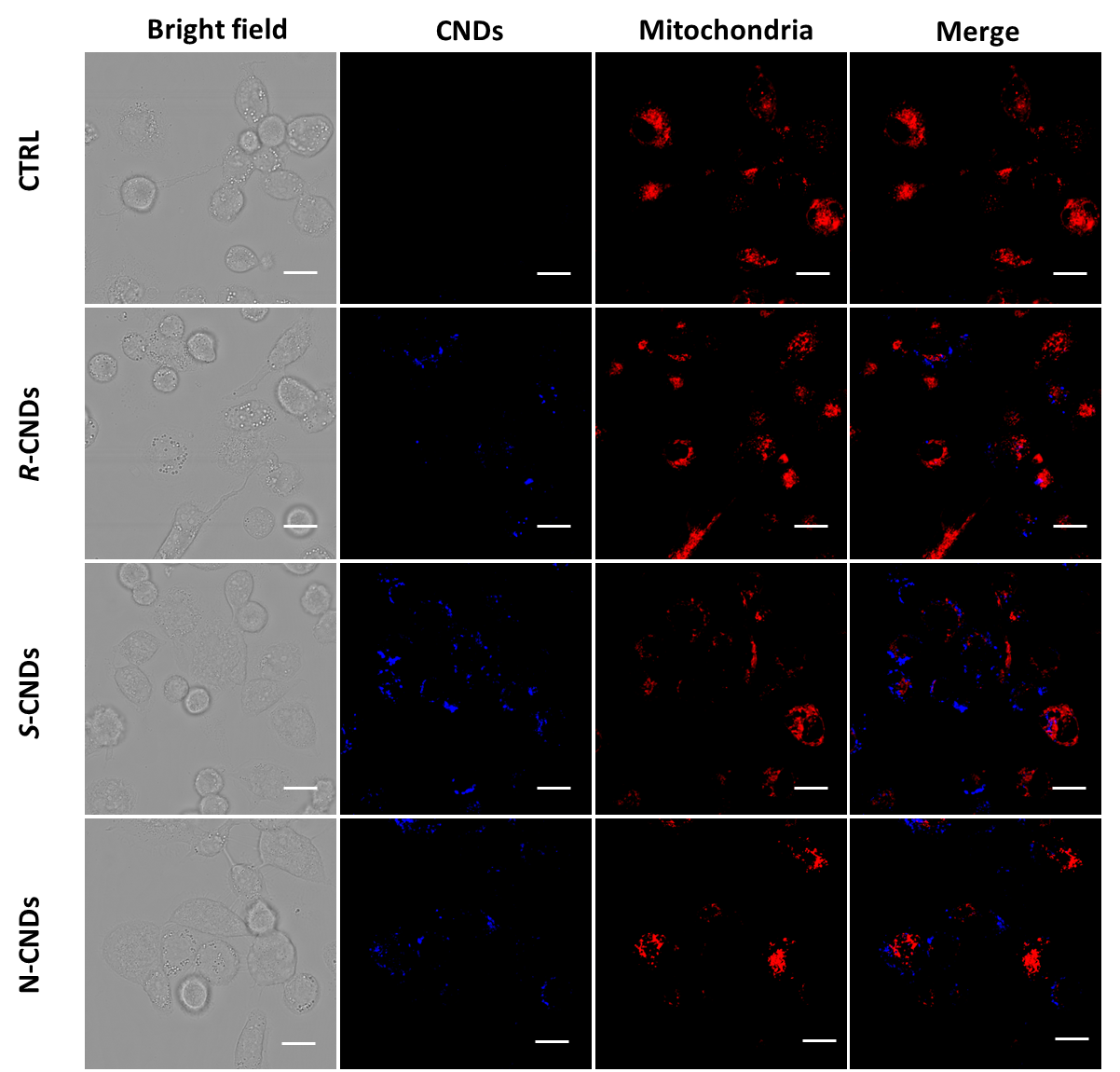
**

**Supplementary Figure 55.** **Mithocondria/CNDs co-localization studies of THP-1 derived macrophages**. Cell were analyzed in 10% FBS containing RPMI 1640 medium after 24 h exposure to *R-*, *S-*, and N*-*CNDs (blue fluorescence channel). Mitochondria were stained by MitoTracker™ Deep Red ^FM^ (red fluorescence channel) at a concentration of 400 nM. The scale bar represents 20 μm. The experiments were repeated independently for three times (n = 3) and representative images are shown.


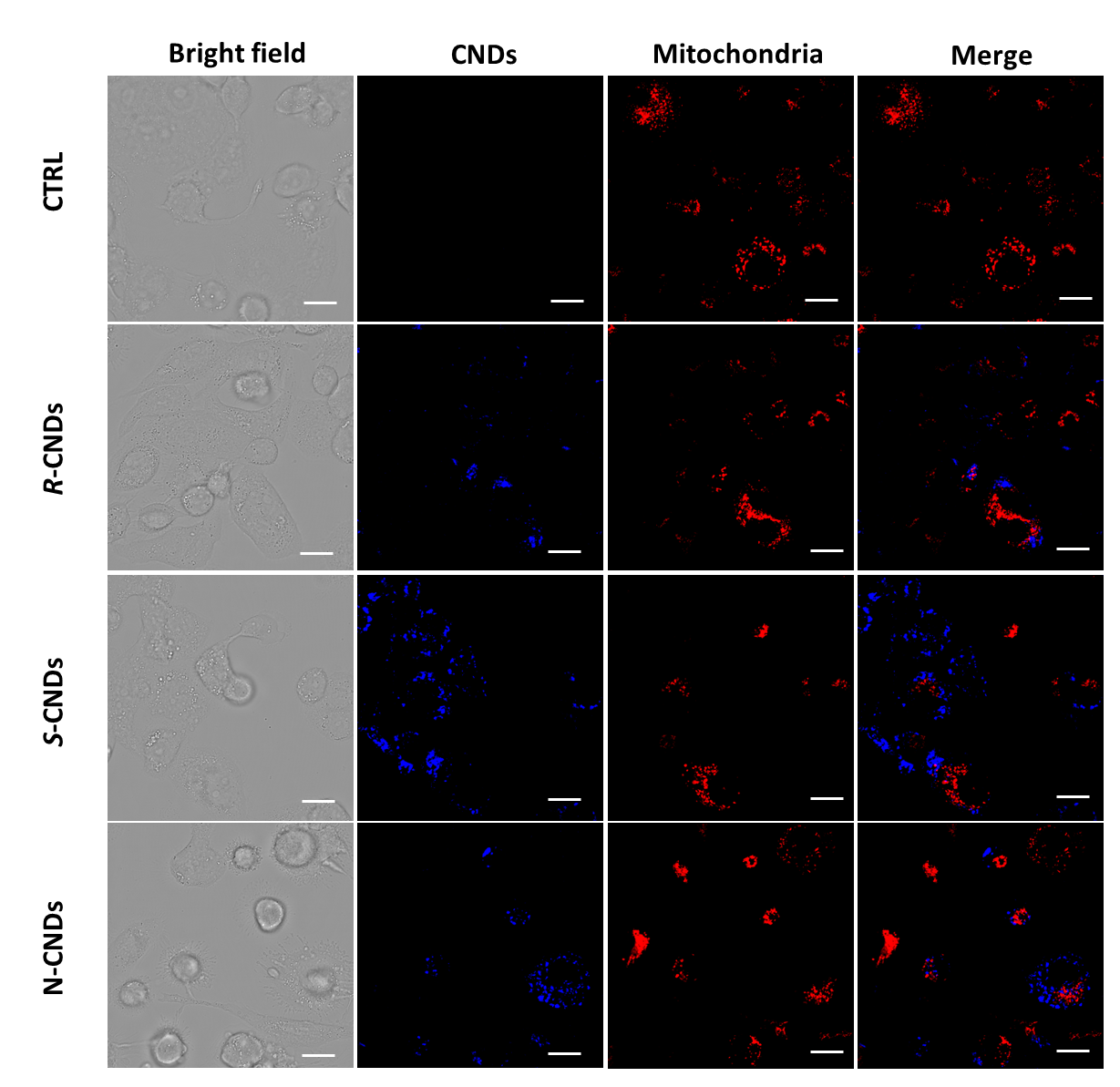


**Supplementary Figure 56.** **Mithocondria/CNDs co-localization studies of THP-1 derived macrophages**. Cells were analyzed in 10% FBS containing RPMI 1640 medium after 48 h exposure to *R-*, *S-*, and N*-*CNDs (blue fluorescence channel). Mitochondria were stained by MitoTracker™ Deep Red ^FM^ (red fluorescence channel) at a concentration of 400 nM. The scale bar represents 20 μm. The experiments were repeated independently for three times (n = 3) and representative images are shown.


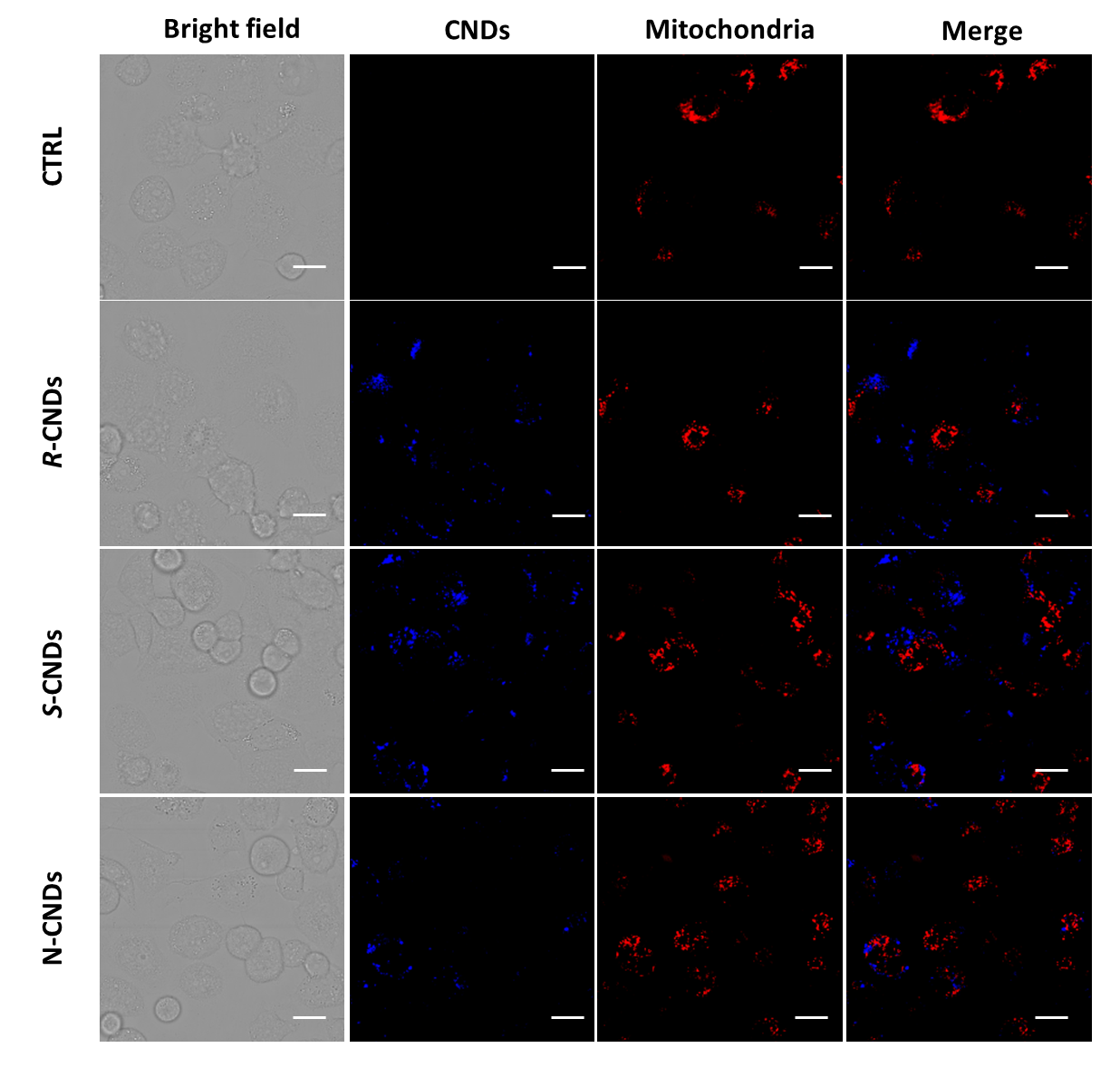


**Supplementary Figure 57.** **Mithocondria/CNDs co-localization studies of THP-1 derived macrophages**. Cells were analyzed in serum-free RPMI 1640 medium after 24 h exposure to *R-*, *S-*, and N*-*CNDs (blue fluorescence channel). Mitochondria were stained by MitoTracker™ Deep Red ^FM^ (red fluorescence channel) at a concentration of 400 nM. The scale bar represents 20 μm. The experiments were repeated independently for three times (n = 3) and representative images are shown.


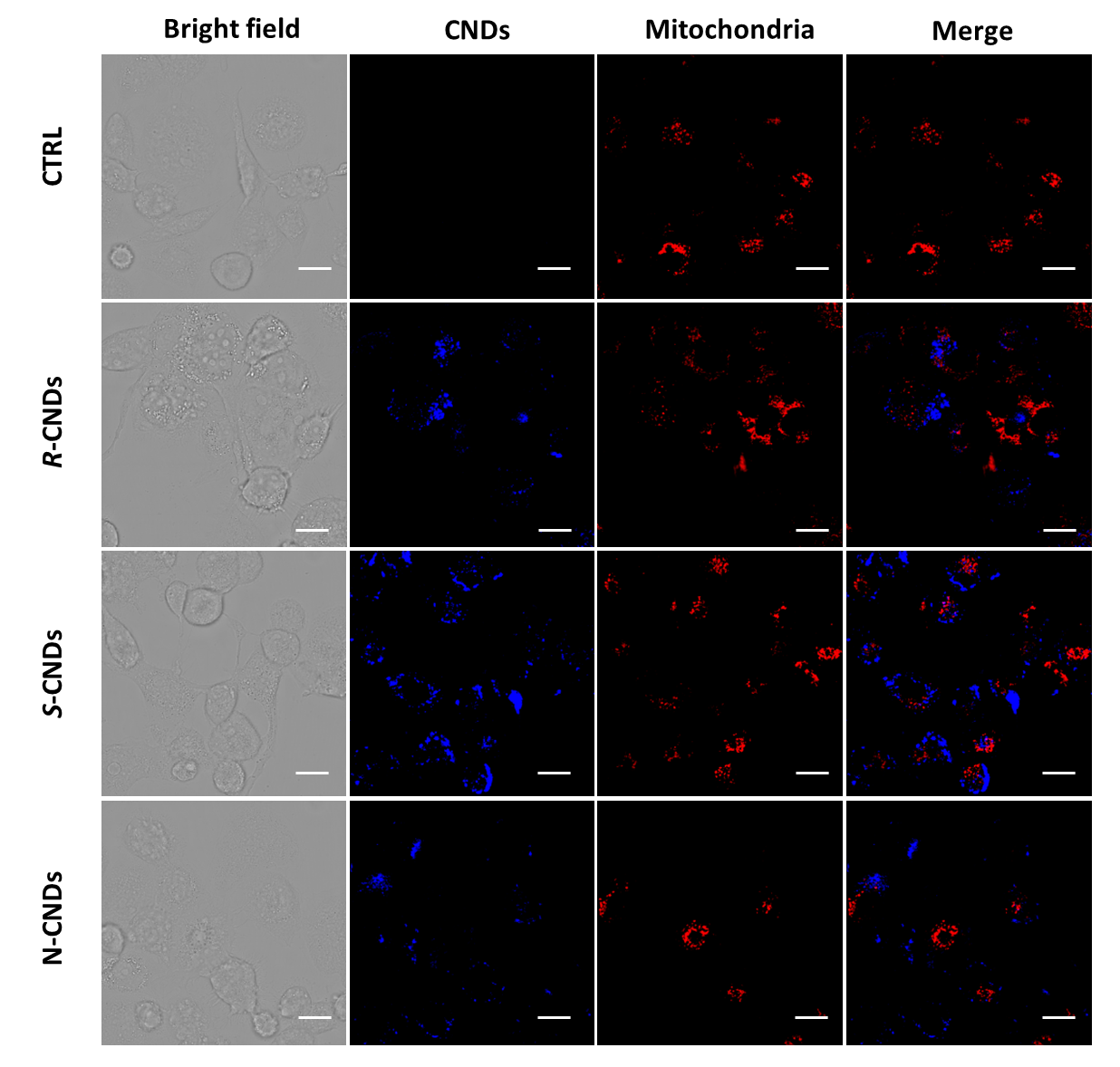


**Supplementary Figure 58.**  **Mithocondria/CNDs co-localization studies of THP-1 derived macrophages**. Cells were analyzed in serum-free RPMI 1640 medium after 48 h exposure to *R-*, *S-*, and N*-*CNDs (blue fluorescence channel). Mitochondria were stained by MitoTracker™ Deep Red ^FM^ (red fluorescence channel) at a concentration of 400 nM. The scale bar represents 20 μm.

The experiments were repeated independently for three times (n = 3) and representative images are shown.

***
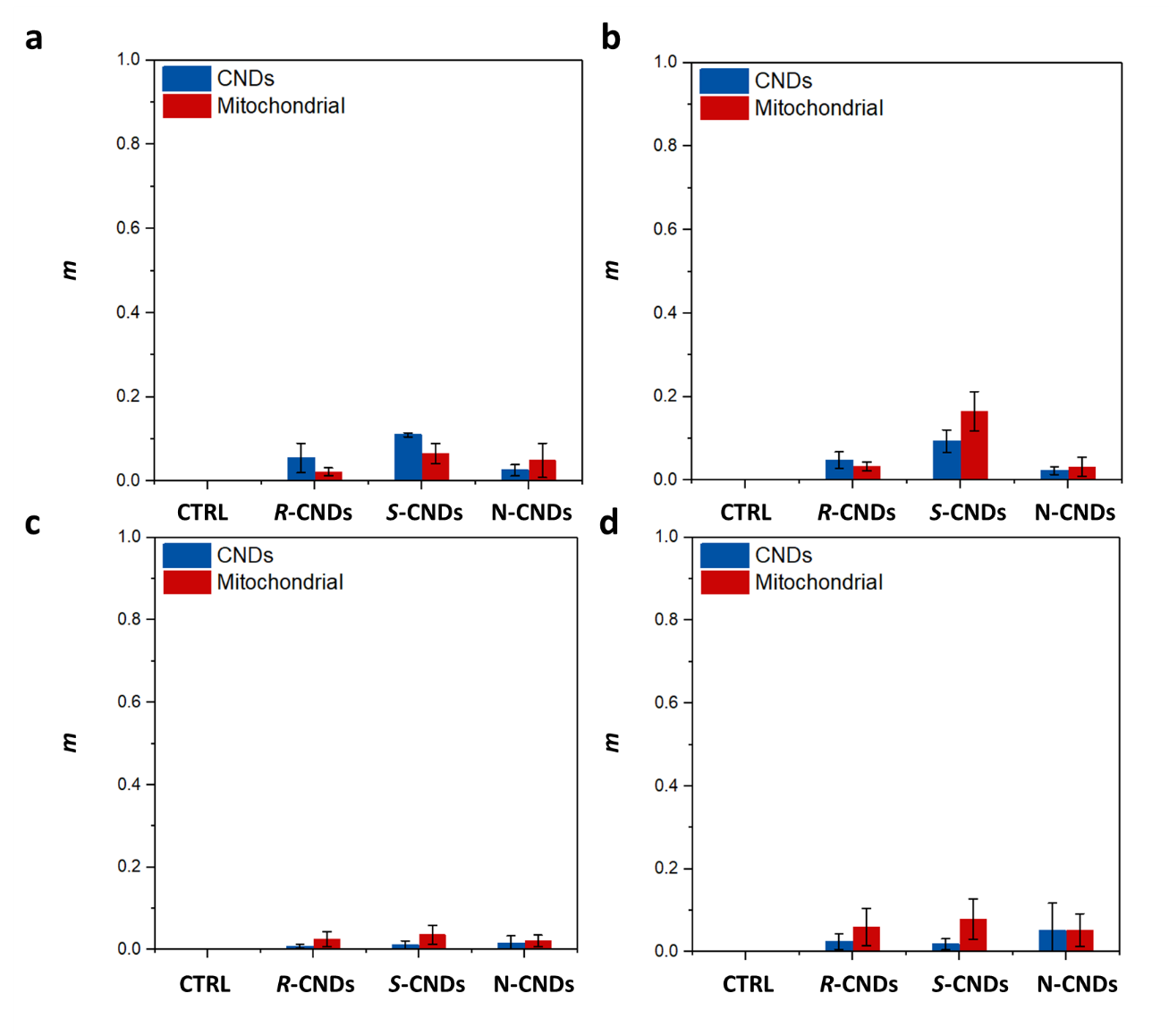
***

**Supplementary Figure 59.** **Overlap degree of CNDs and MitoTracker™ Deep Red^FM^ stained mitochondria (Manders' coefficients) in THP-1 derived macrophages**. (**a**,**b**) 24 or (**c**,**d**) 48 h exposure to CNDs in 10% (left) or 0% (right) FBS containing RPMI 1640 medium. Examples for the raw data from which Manders' coefficients have been calculated are provided in Supplementary Figure 55, 56, 57 and 58. The blue bars show Manders' coefficient *m*_1_, i.e. the percentage of blue fluorescent pixels (i.e. parts of the cells that contained CNDs) that overlapped with red fluorescent pixels (i.e. parts of the cells belonging to mitochondria). The red bars show Manders' coefficient *m*_2_, the percentage of red fluorescent pixels (i.e. parts of cells belonging to mitochondria) which overlapped with blue fluorescent pixels (i.e. parts of the cells which contained CNDs). The control (CTRL) refers to cells which have not been exposed to CNDs but have been stained with MitoTracker™ Deep Red^FM^. No fluorescence was detected in the control samples. n ≥ 210 cells in 21 images from three independent experiments were analyzed for each group. Results are shown as mean values with error bars (i.e., the corresponding standard deviations) from three independent samples (n = 3) over three independent experiments. Data indicate that the CNDs and mitochondria do not significantly co-localize.


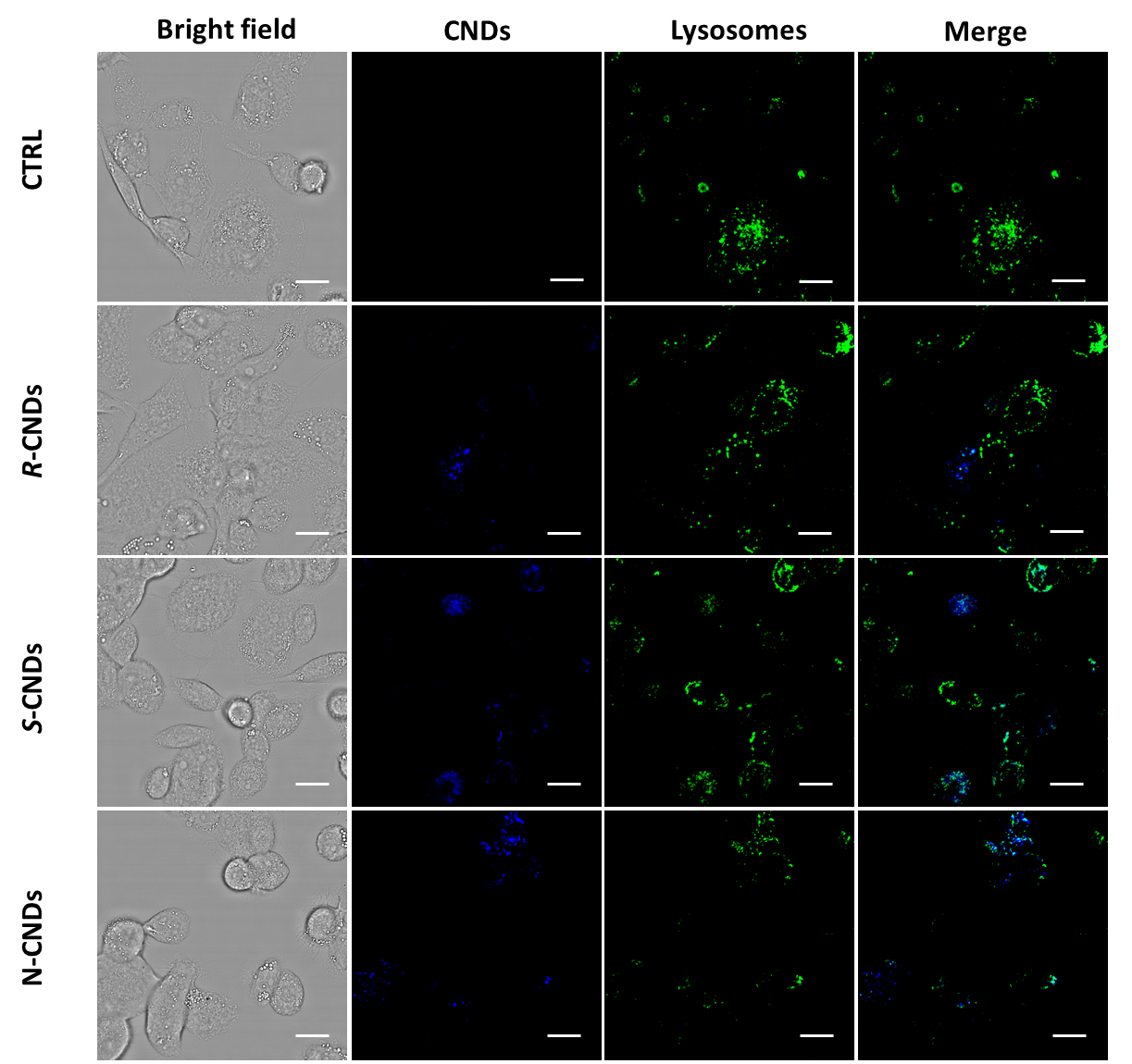


**Supplementary Figure 60.** **Lysosomes/CNDs co-localization studies of THP-1 derived macrophages**. Cells were analyzed in 10% FBS containing RPMI 1640 medium after 24 h exposure to *R-*, *S-*, and N*-*CNDs (blue fluorescence channel). Lysosomes were stained by LysoTracker™ Green DND-26 (green fluorescence channel). The scale bar represents 20 μm. The experiments were repeated independently for three times (n = 3) and representative images are shown.


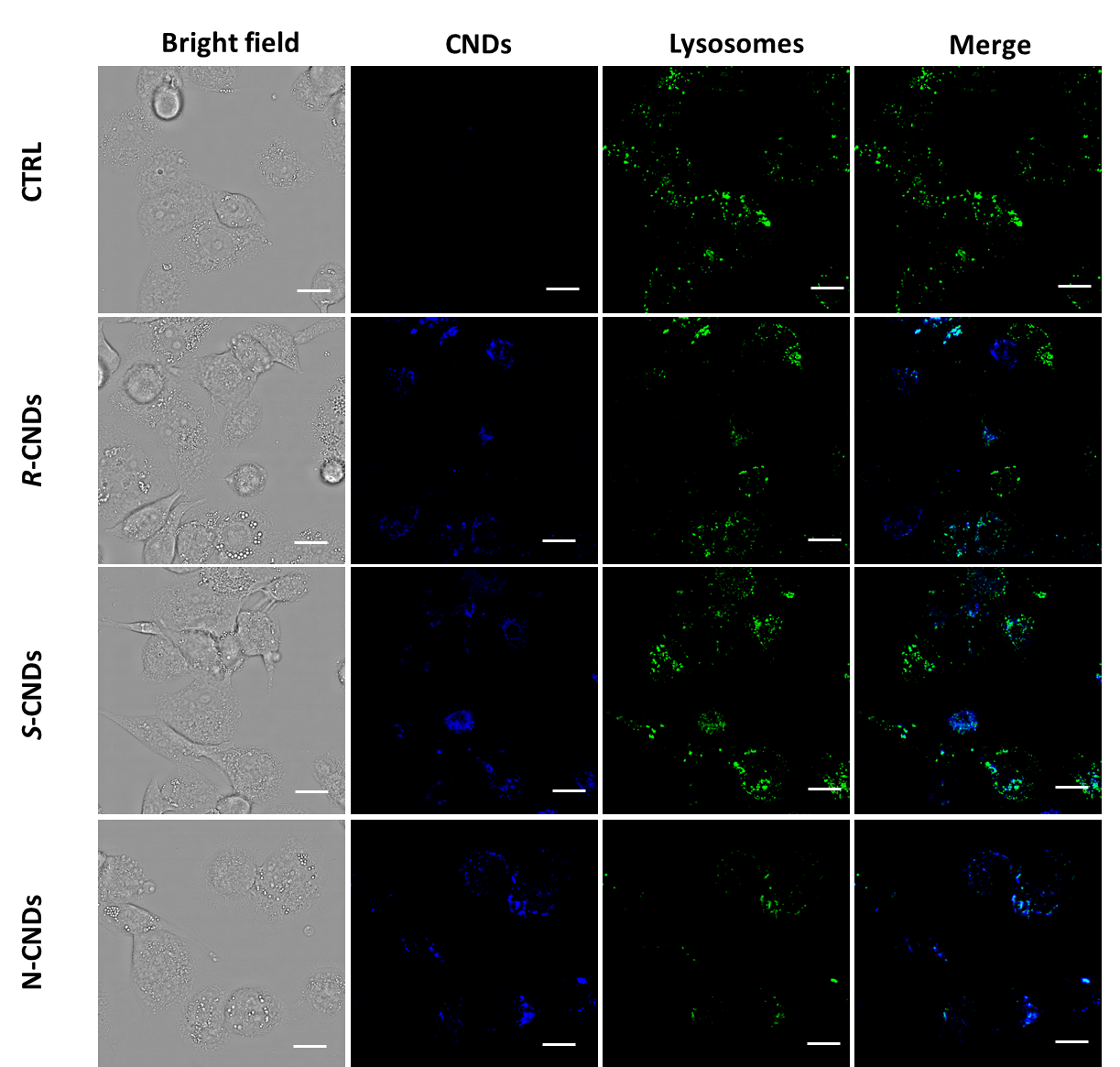


**Supplementary Figure 61.** **Lysosomes/CNDs co-localization studies of THP-1 derived macrophages**. Cells were analyzed in 10% FBS containing RPMI 1640 medium after 48 h exposure to *R-*, *S-*, and N*-*CNDs (blue fluorescence channel). Lysosomes were stained by LysoTracker™ Green DND-26 (green fluorescence channel). The scale bar represents 20 μm. The experiments were repeated independently for three times (n = 3) and representative images are shown.


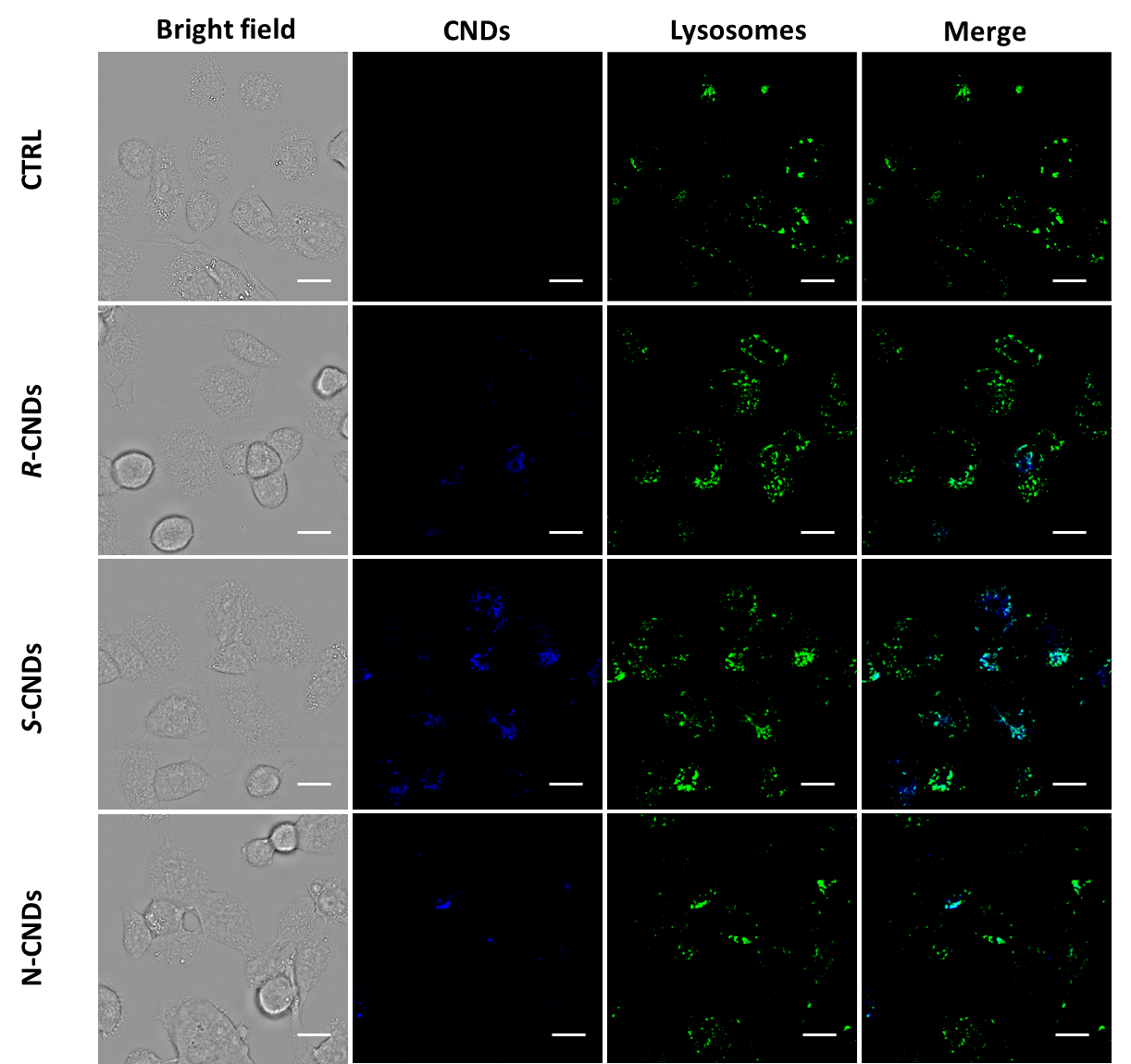


**Supplementary Figure 62.** **Lysosomes/CNDs co-localization studies of THP-1 derived macrophages**. Cells were analyzed in serum-free RPMI 1640 medium after 24 h exposure to *R-*, *S-*, and N*-*CNDs (blue fluorescence channel). Lysosomes were stained by LysoTracker™ Green DND-26 (green fluorescence channel). The scale bar represents 20 μm. The experiments were repeated independently for three times (n = 3) and representative images are shown.


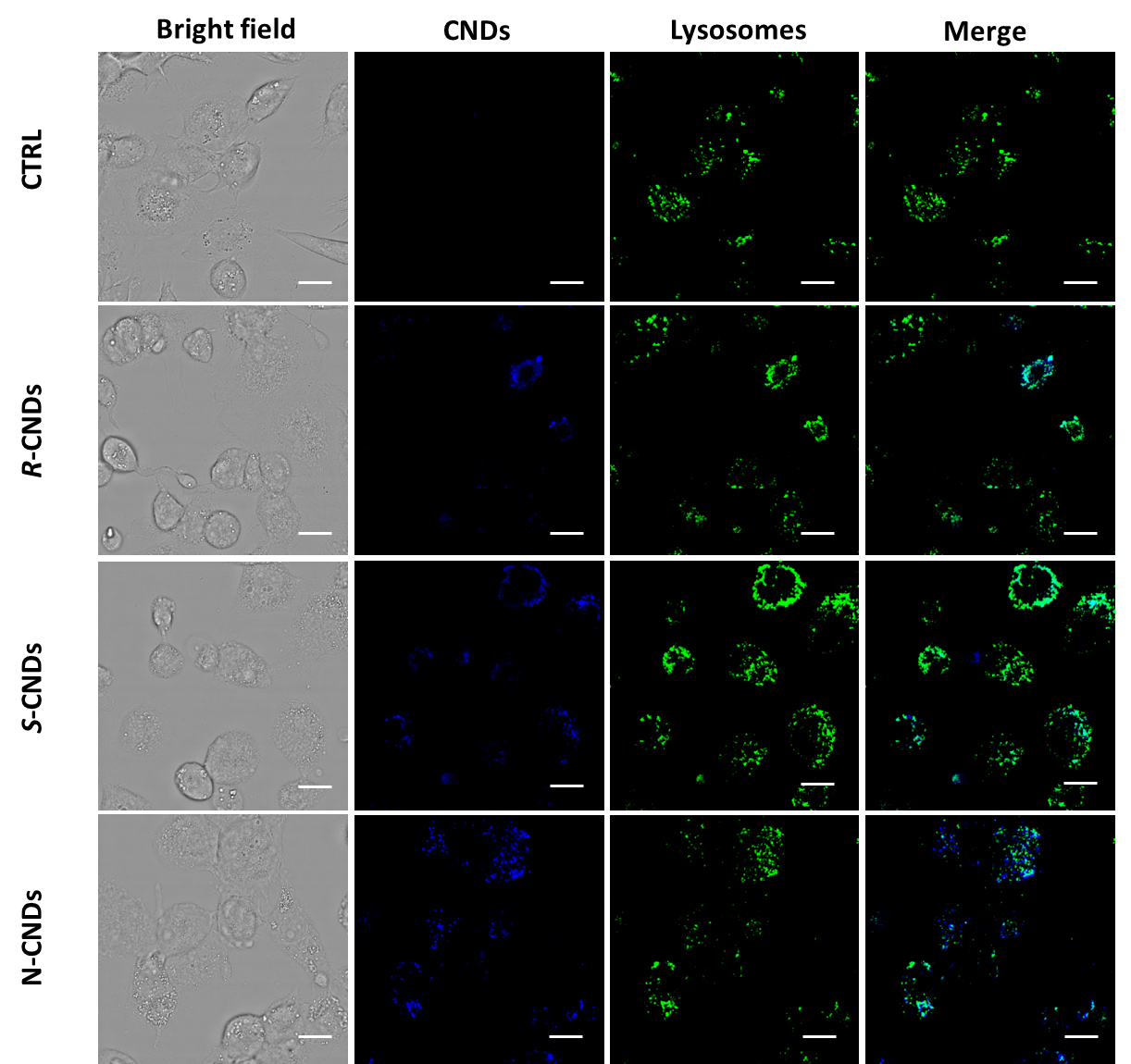


**Supplementary Figure 63.** **Lysosomes/CNDs co-localization studies of THP-1 derived macrophages**. Cells were analyzed in serum-free RPMI 1640 medium after 48 h exposure to *R-*, *S-*, and N*-*CNDs (blue fluorescence channel). Lysosomes were stained by LysoTracker™ Green DND-26 (green fluorescence channel). The scale bar represents 20 μm. The experiments were repeated independently for three times (n = 3) and representative images are shown.


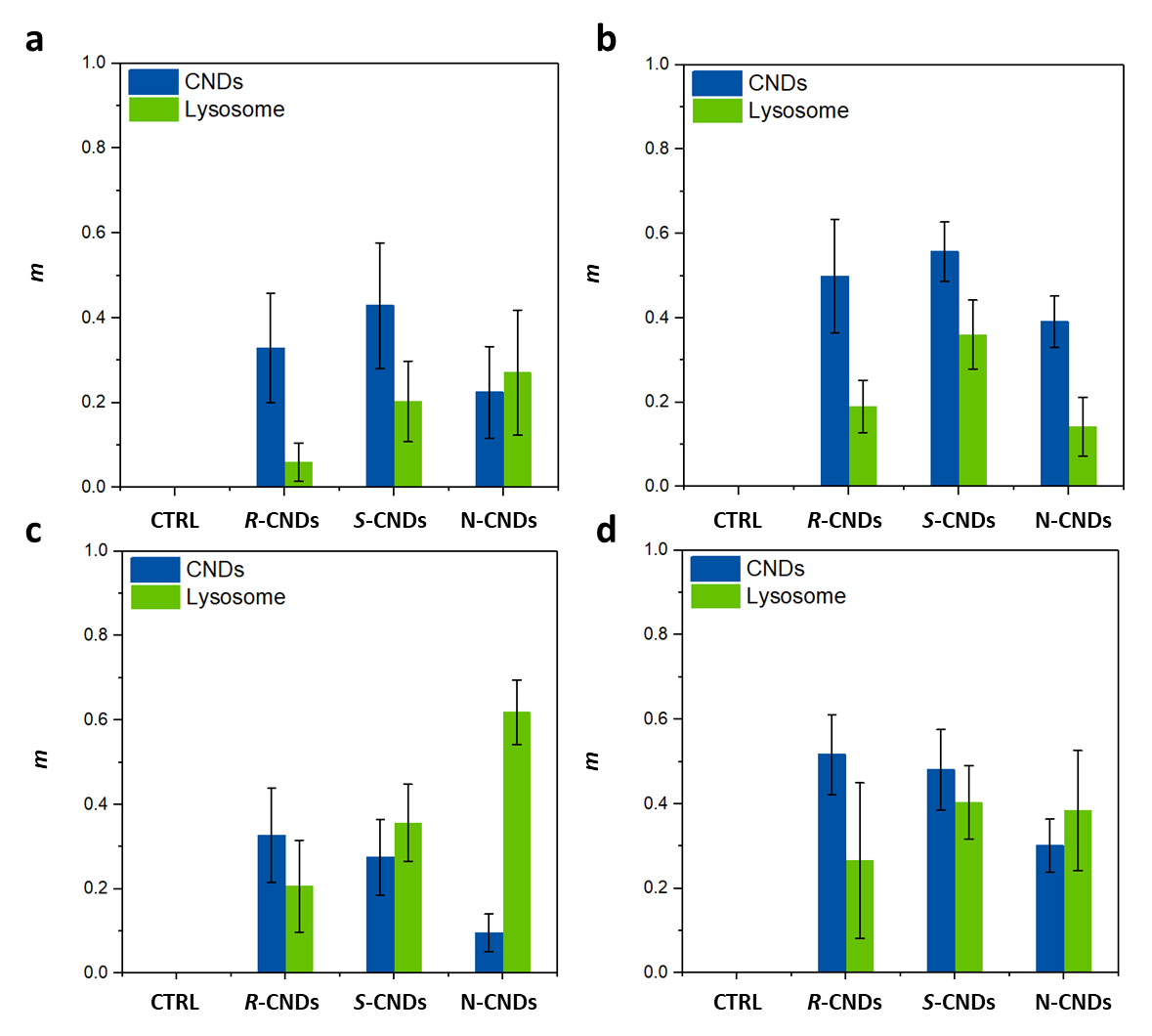


**Supplementary Figure 64.** **Overlap degree of CNDs and stained lysosomes (Manders' coefficients) in THP-1 derived macrophages**. (**a**,**b**) 24 or (**c**,**d**) 48 h exposure to CNDs in 10% (left) or 0% (right) FBS containing RPMI 1640 medium. Examples for the raw data from which Manders' coefficients have been calculated are provided in Supplementary Figure 60, 61, 62 and 63. The blue bars show Manders' coefficient *m*_1_, i.e. the percentage of blue fluorescent pixels (i.e. parts of the cells that contained CNDs) that overlapped with green fluorescent pixels (i.e. parts of the cells belonging to lysosomes). The green bars show Manders' coefficient *m*_2_, the percentage of green fluorescent pixels (i.e. parts of cells belonging to lysosomes) which overlapped with blue fluorescent pixels (i.e. parts of the cells which contained CNDs). The control (CTRL) refers to cells that have not been exposed to CNDs but have been stained with LysoTracker™ Green DND-26. No fluorescence was detected in the control samples. n ≥ 210 cells in 21 images from three independent experiments were analyzed for each group. Results are shown as mean values with error bars (i.e., the corresponding standard deviations) from three independent samples (n = 3) over three independent experiments. Data indicate that the CNDs are largely localized in lysosomes (high *m*_1_ values). Note that there is a certain degree of overexposure in the CND channel. Thus the fluorescence area of CNDs seems larger than the fluorescence area of lysosomes, which is a clear artifact due to overexposure. *m*_2_ thus cannot be quantitatively compared to *m*_1_. The data are still presented here as a demonstration for potential errors which rule out quantitative analysis. The only conclusion which can be drawn in a non*-*quantitative way is that the CNDs largely co-localize with lysosomes.


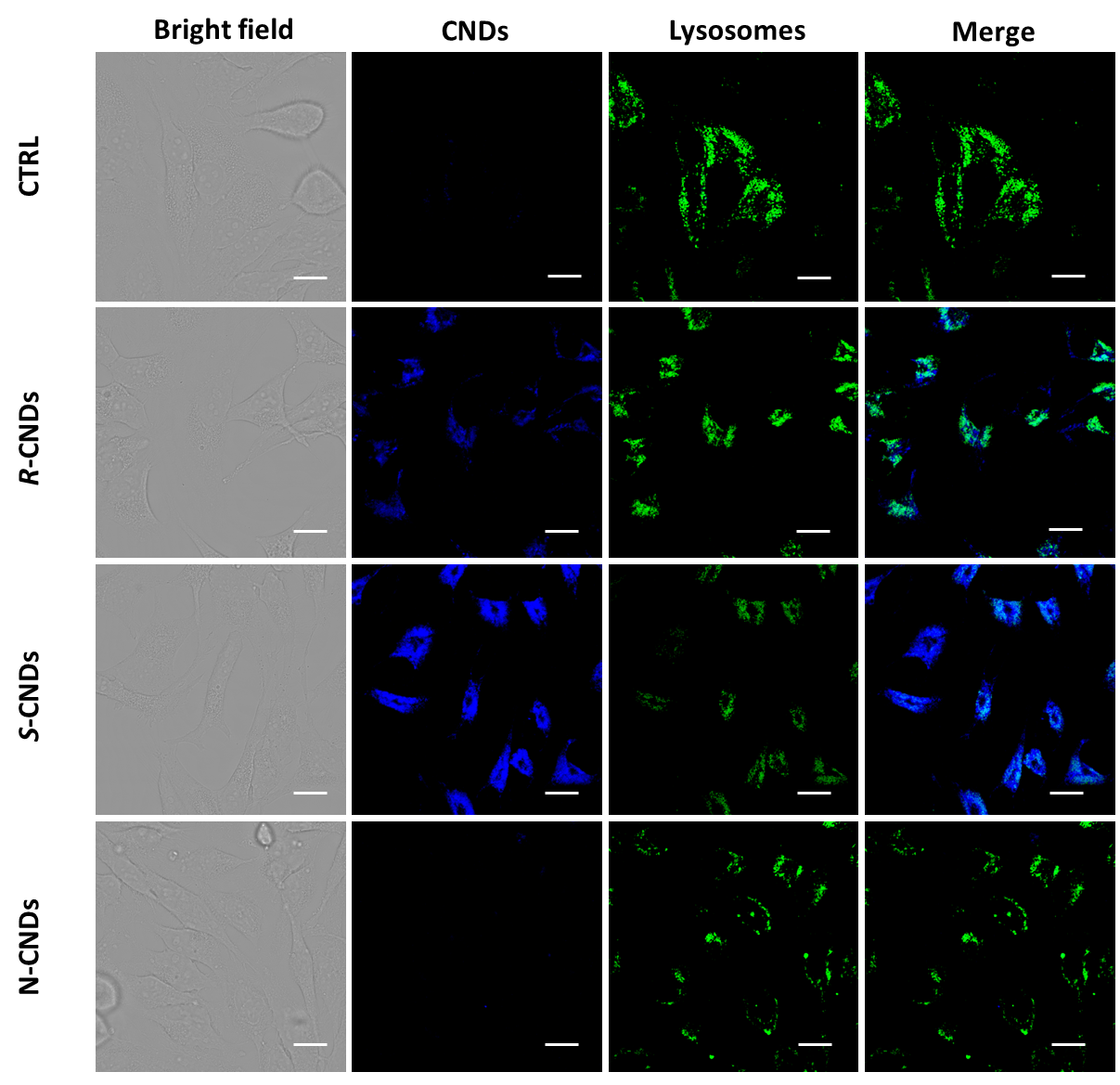


**Supplementary Figure 65. Lysosomes/CNDs co-localization studies of Hela cells**. Cells were analyzed in 10% FBS containing DMEM medium after 48 h exposure to *R-*, *S-*, and N*-*CNDs (blue fluorescence channel). Lysosomes were stained by LysoTracker™ Green DND-26 (green fluorescence channel). The scale bar represents 20 μm. The experiments were repeated independently for three times (n = 3) and representative images are shown.


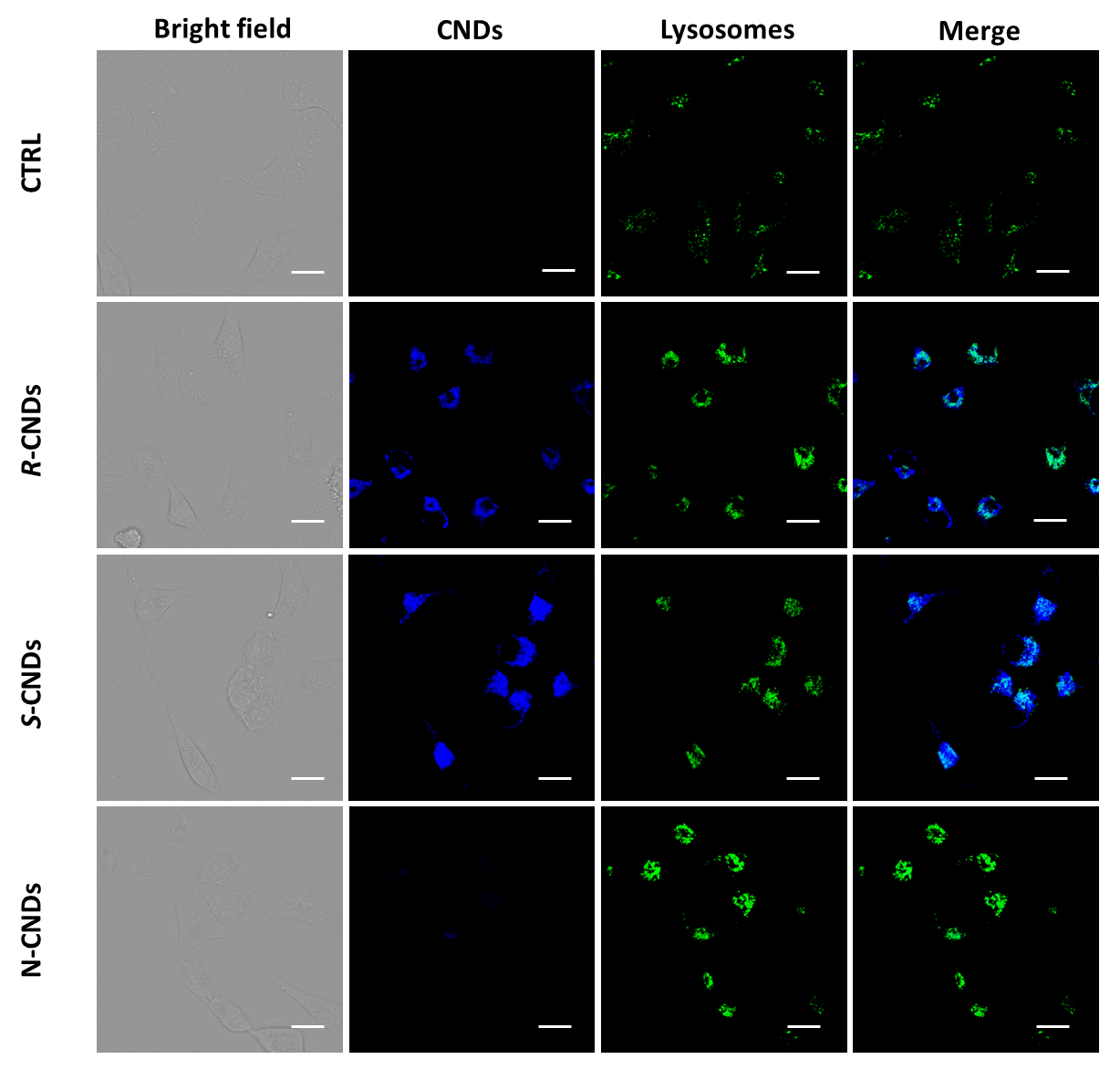


**Supplementary Figure 66.** **Lysosomes/CNDs co-localization studies of Hela cells**. Cells were analyzed in serum-free DMEM medium after 24 h exposure to *R-*, *S-*, and N*-*CNDs (blue fluorescence channel). Lysosomes were stained by LysoTracker™ Green DND-26 (green fluorescence channel). The scale bar represents 20 μm. The experiments were repeated independently for three times (n = 3) and representative images are shown.


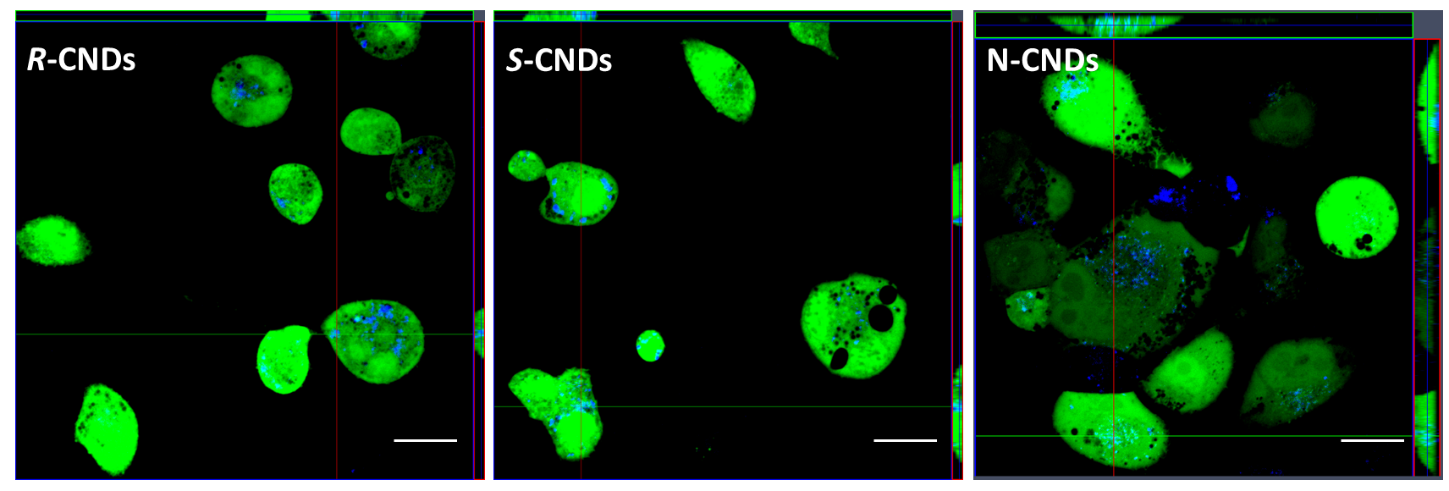


**Supplementary Figure 67. Z-stack CLSM images of THP-1 derived macrophages exposed to CNDs.** Cells were exposed to CNDs for 24 h at a concentration of *C*'_CNDs_ = 400 μg mL^–1^ in 10% FBS containing RPMI 1640 medium (blue fluorescence channel). Cells were also stained with Calcein AM (green fluorescence channel). Only one plane out of several z-stacks is shown. From the z-stacks cross*-*sections along the red and green lines could be made (i.e. the height profiles perpendicular to the imaging plane). The cross*-*sections are show on the bottom and on the left of each image. The scale bars indicated 20 μm. The experiment was conducted for one time (n = 1) and representative images are shown.


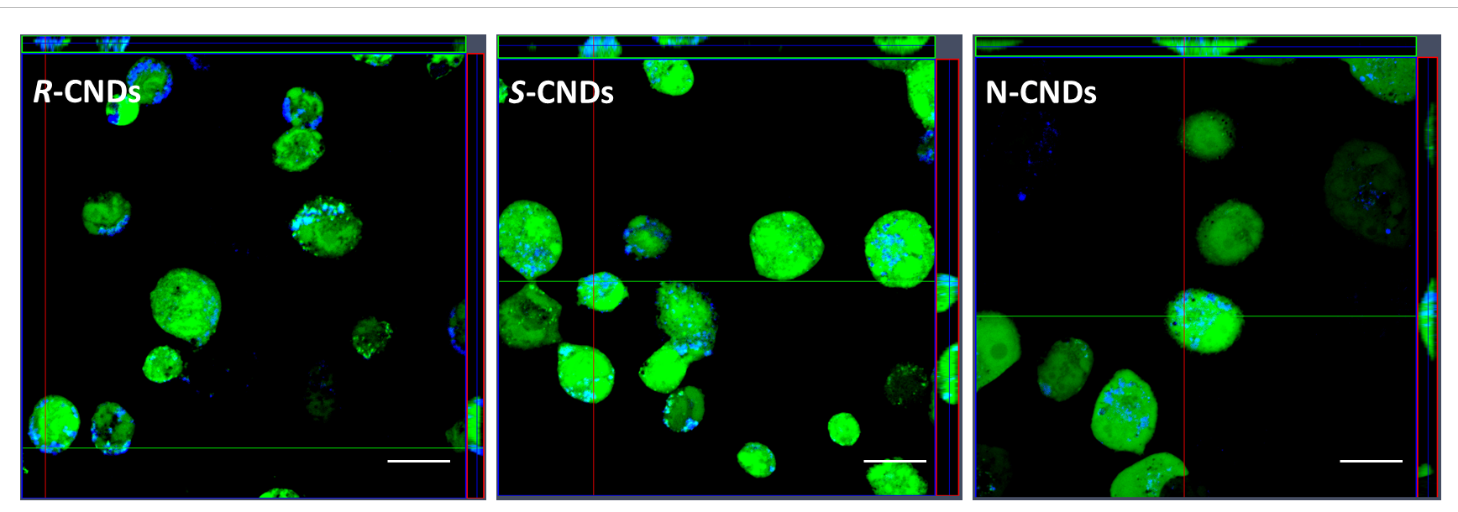


**Supplementary Figure 68. Z-stack CLSM images of THP-1 derived macrophages exposed** **to CNDs**. Cells were exposed to CNDs for 24 h at a concentration of *C*'_CNDs_ = 400 μg mL^–1^ in serum-free RPMI 1640 medium (blue fluorescence channel). Cells were also stained with Calcein AM (green fluorescence channel). Only one plane out of several z-stacks is shown. From the z-stacks cross*-*sections along the red and green lines could be made (i.e. the height profiles perpendicular to the imaging plane). The cross*-*sections are show on the bottom and on the left of each image. The scale bars indicated 20 μm. The experiment was conducted for one time (n = 1) and representative images are shown.

**
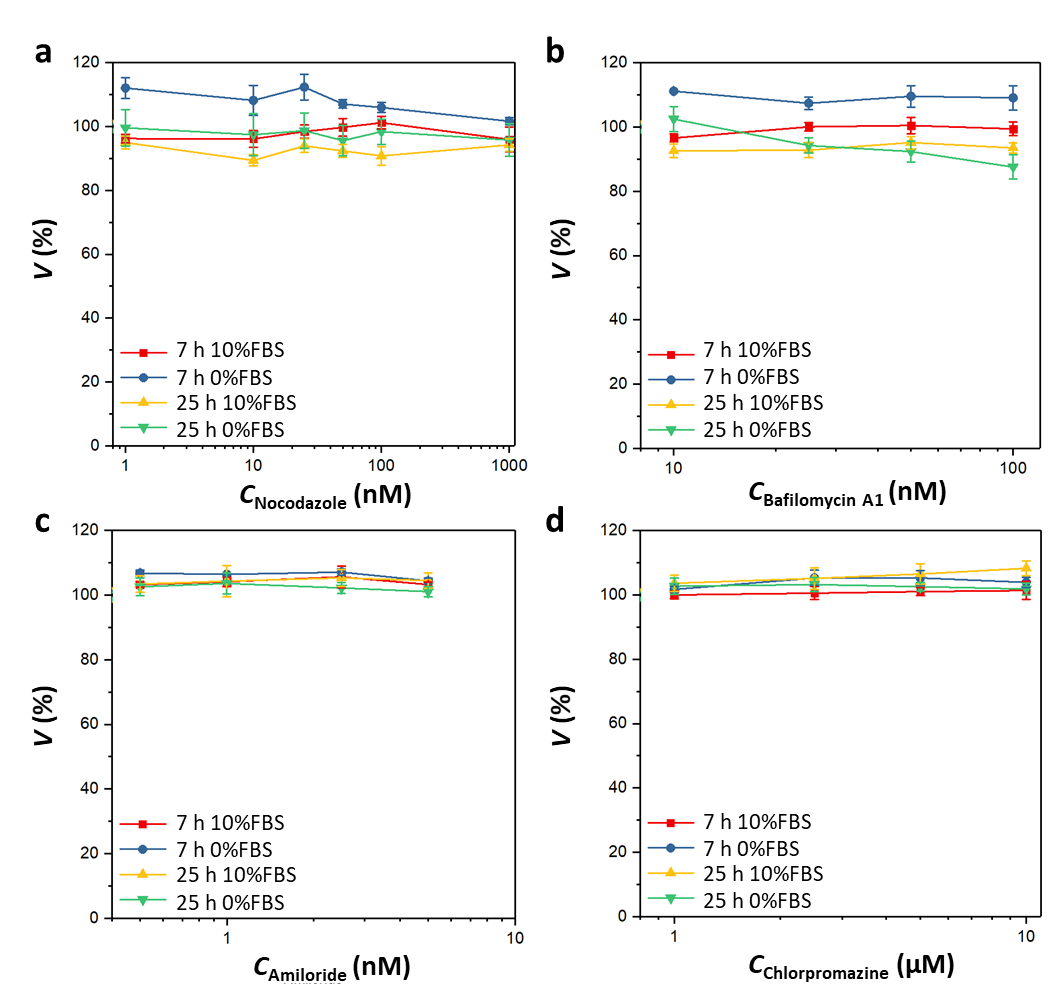
**

**Supplementary Figure 69. Cell viability V of THP-1 derived macrophages after exposed to cellular uptake inhibitors**. Cells were treated with (**a**) Nocodazole, (**b**) Bafilomycin A1, (**c**) Amiloride, and (**d**) Chlorpromazine for 7 h or 25 h in 10% or 0% FBS containing RPMI 1640 medium, as measured by the resazurin assay. Resazurin was added at a final concentration of 0.025 mg mL^–1^ and the cells were further incubated for 4 h before fluorescence analysis by a fluorimeter. Results are shown as mean values with error bars (i.e., the corresponding standard deviations) from three independent samples (n = 3) over three independent experiments.

**
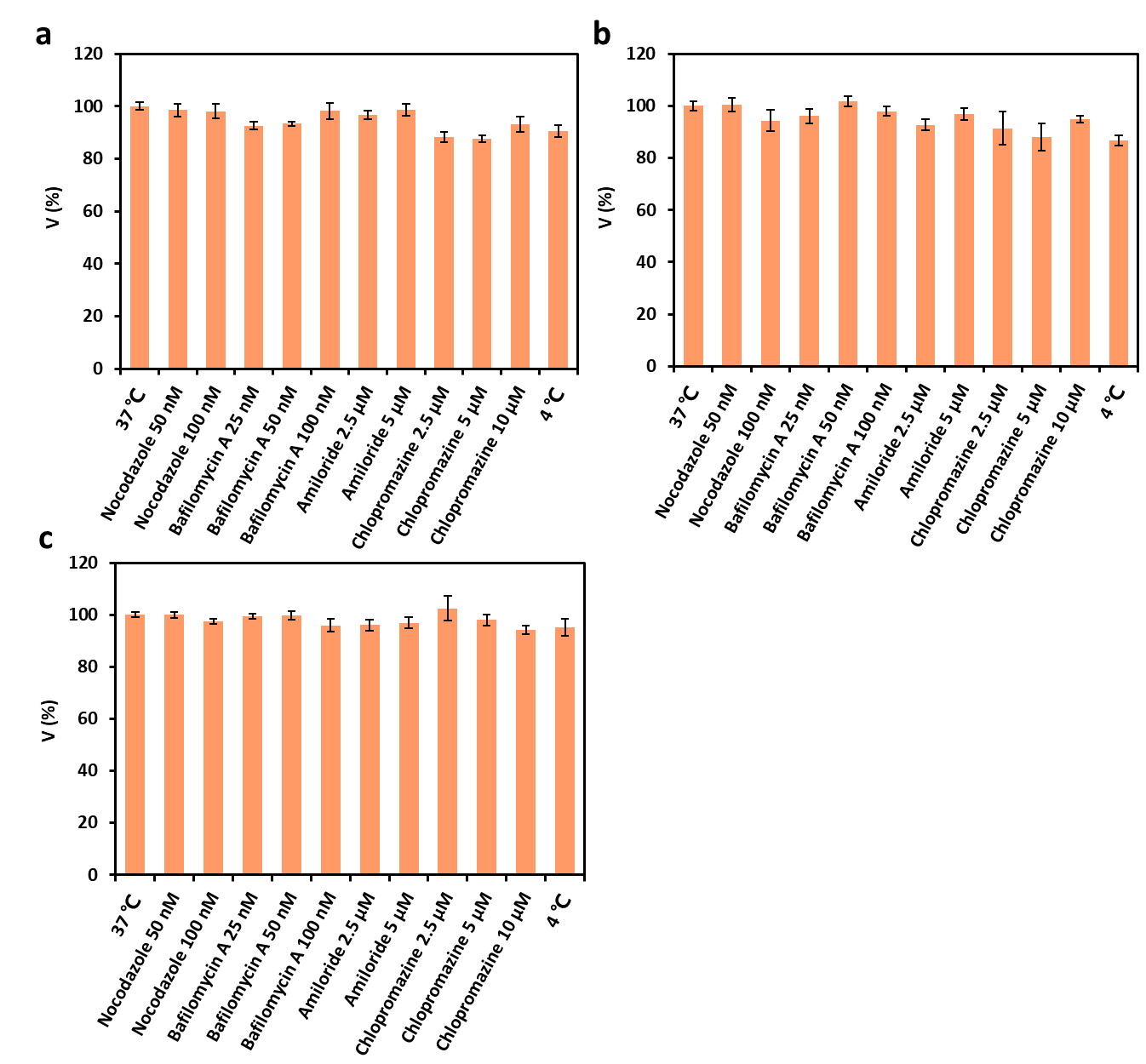
**

**Supplementary Figure 70. Cell viability V of THP-1-derived macrophages after exposed to cellular uptake inhibitors for 1 h followed by 6 h incubation with CNDs.** Cells were exposed to (**a**) *R-*, (**b**) *S-* or (**c**) N*-*CNDs at a concentration of *C*'_CNDs_ = 400 μg mL^–1^ in 10% FBS containing RPMI 1640 medium as measured by the resazurin assay. Resazurin was added at a final concentration of 0.025 mg mL^–1^ and the cells were further incubated for 4 h before analysis. Results are shown as mean values with error bars (i.e., the corresponding standard deviations) from three independent samples (n = 3) over three independent experiments.


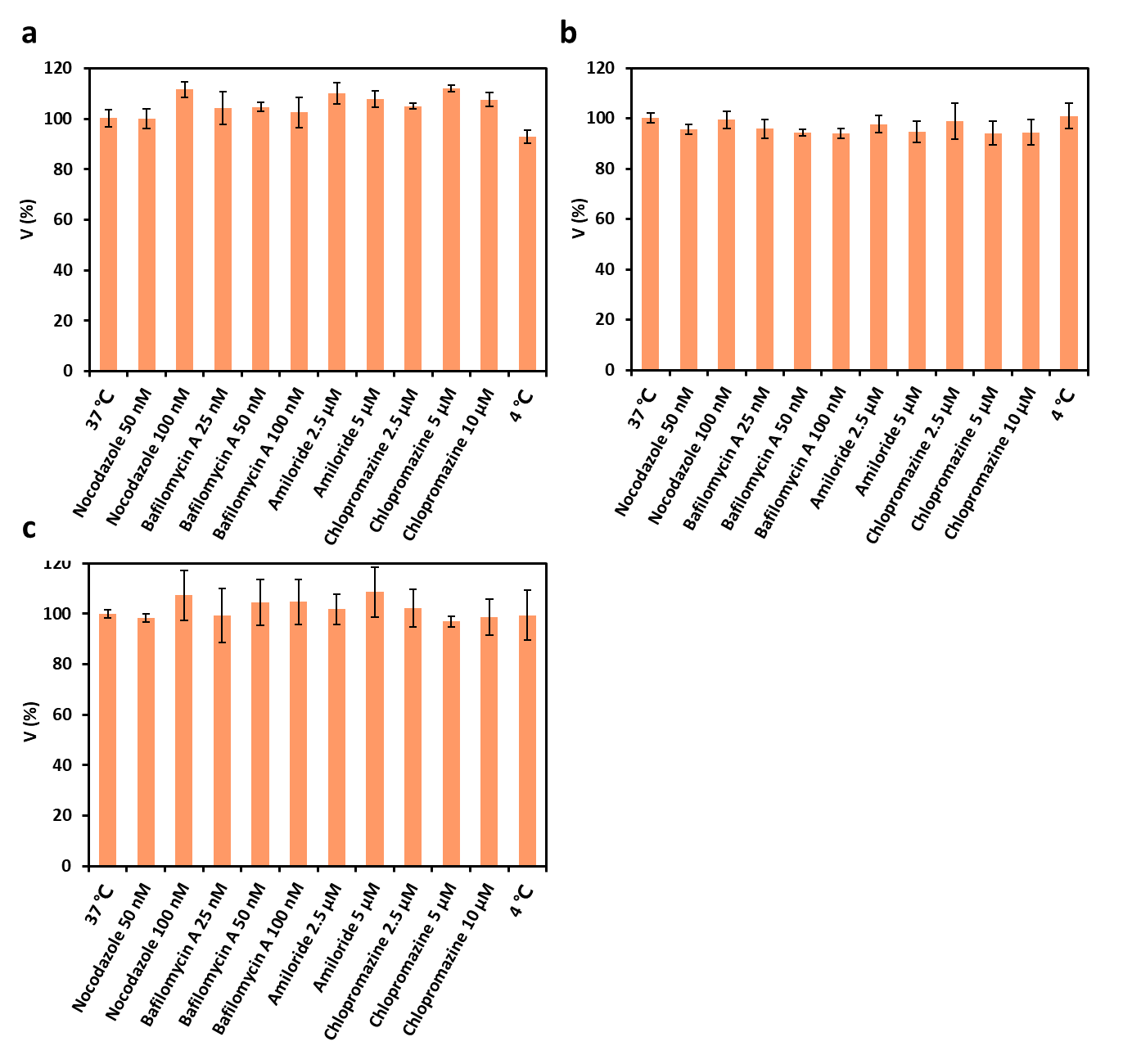


**Supplementary Figure 71. Cell viability V of THP-1-derived macrophages after exposed to cellular uptake inhibitors for 1 h followed by 6 h incubation with** **CNDs**. Cells were exposed to (**a**) *R-*, (**b**) *S-* or (**c**) N*-*CNDs at a concentration of *C*'_CNDs_ = 400 μg mL^–1^ in serum-free RPMI 1640 medium as measured by the resazurin assay. Resazurin was added at a final concentration of 0.025 mg mL^–1^ and the cells were further incubated for 4 h before analysis. Results are shown as mean values with error bars (i.e., the corresponding standard deviations) from three independent samples (n = 3) over three independent experiments.


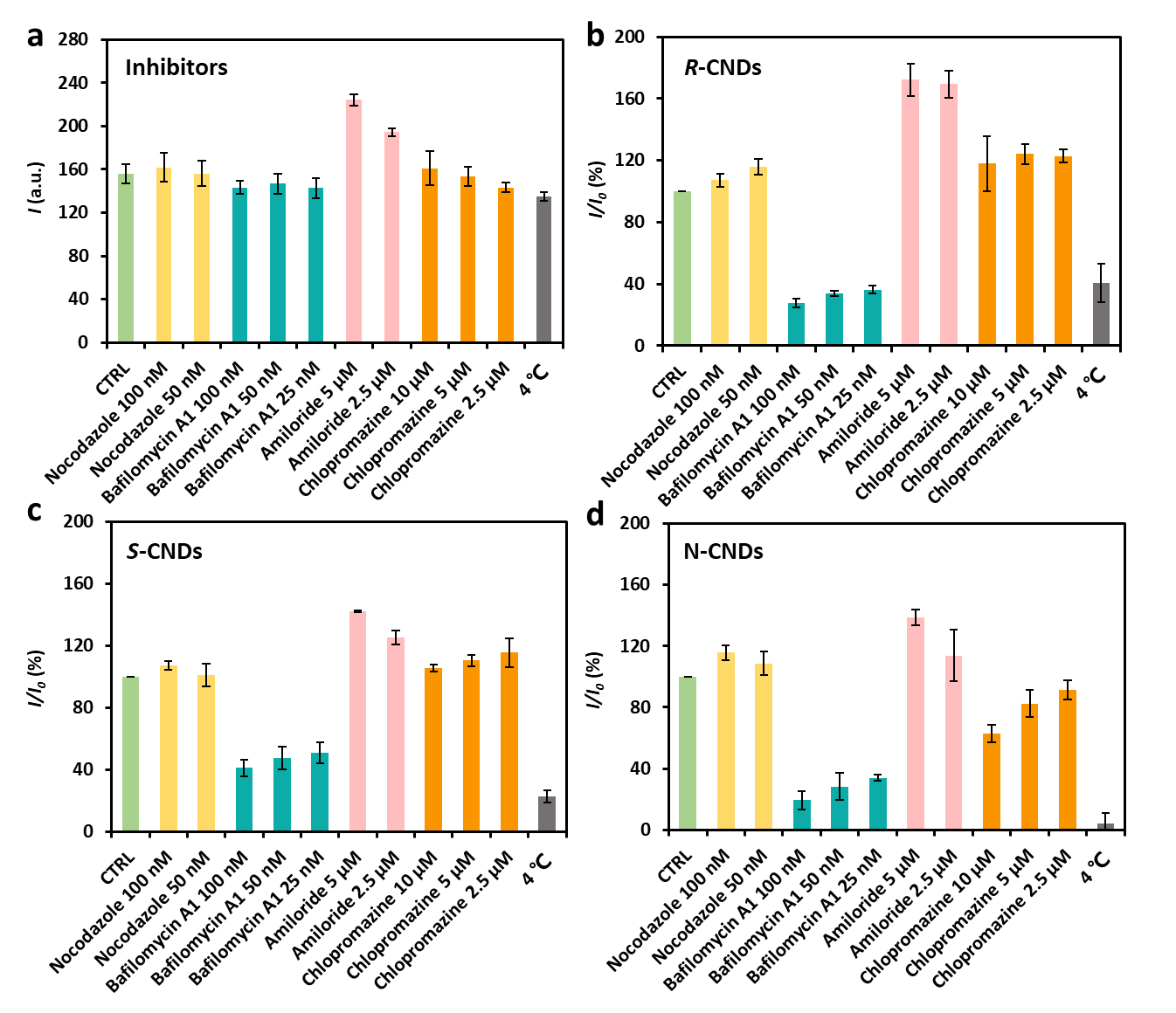


**Supplementary Figure 72**. **Analysis of cell uptake pathway trough flow cytometry**. (**a**) Fluorescence intensity *I* of THP-1 derived macrophages in 10% FBS containing RPMI 1640 medium after exposure to different inhibitors for 7 h. The control (CTRL) are cells to which no inhibitor had been added. As negative control cells without added inhibitors were incubated at 4 °C instead of 37 °C. The fluorescence recorded here with flow cytometry corresponds to the autofluorescence of the cells and the added inhibitors. There is significant autofluorescence due to amiloride. (**b**,**c**,**d**) THP-1 derived macrophages in 10% FBS containing RPMI 1640 medium were exposed for 1 h to the different inhibitors and then in addition with the (**b**) *R-*, (**c**) *S-*, and (**d**) N*-*CNDs (*C*'_CNDs_ = 400 μg mL^–1^) for 6 more h. The fluorescence intensity *I* of the cells due to the internalized CNDs (and due to the autofluorescence of cells and inhibitors) was normalized to the fluorescence intensity *I*_0_ of the control sample (cells incubated with CNDs at 37 °C without addition of inhibitors). *I* *I*_0_^–1^ < 100% indicates that the respective inhibitor blocks CND uptake and that thus the mechanism this inhibitor is blocking is a contributing uptake pathway of the CNDs. Results are shown as mean values with error bars (i.e., the corresponding standard deviations) from three independent samples (n = 3) over three independent experiments.

**
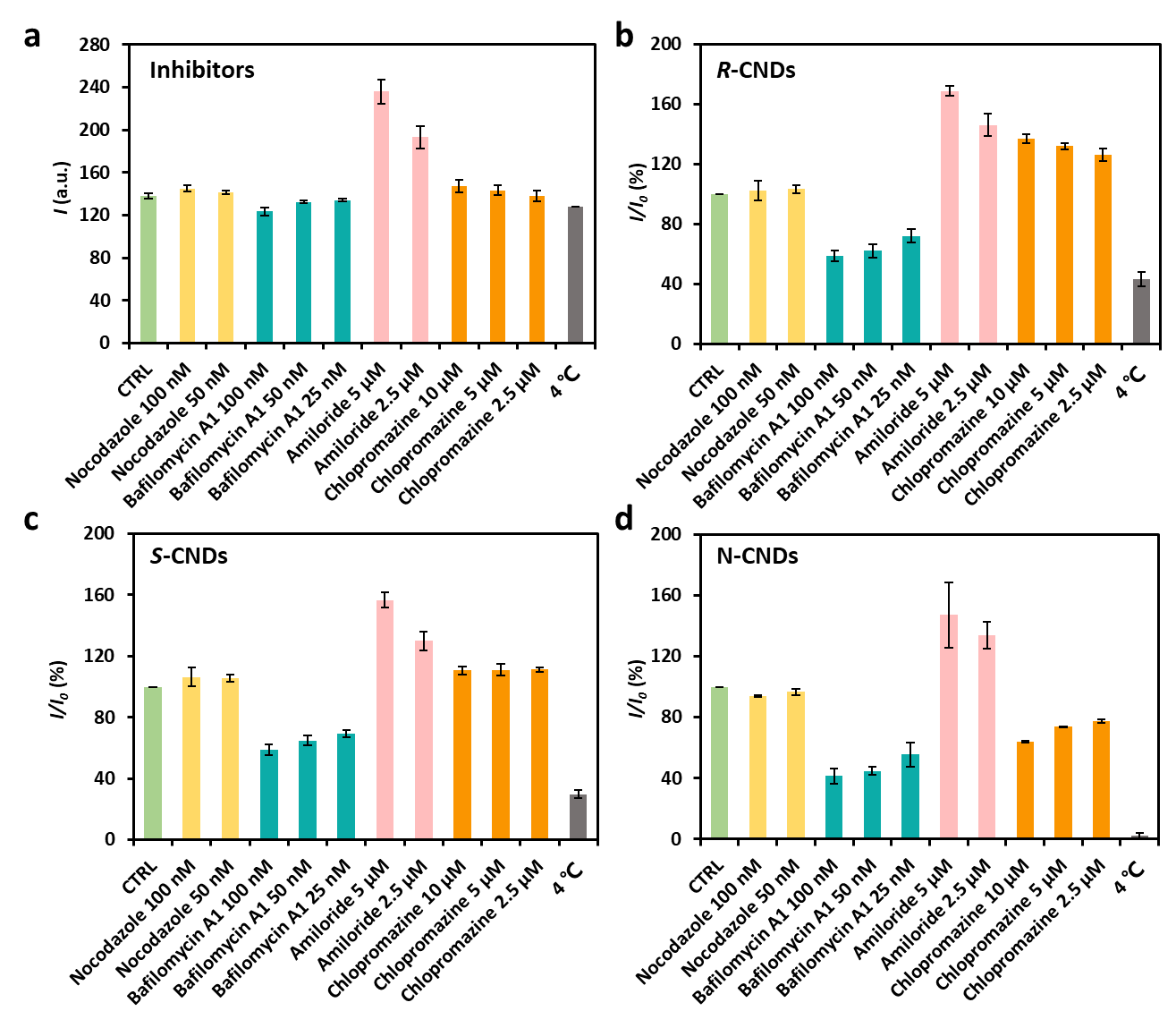
**

**Supplementary Figure 73. Analysis of cell uptake pathway trough flow cytometry**. (**a**) Fluorescence intensity *I* of THP-1 derived macrophages in serum-free RPMI 1640 medium after exposure to different inhibitors for 7 h. The control (CTRL) are cells to which no inhibitor had been added. As negative control cells without added inhibitors were incubated at 4 °C instead of 37 °C. The fluorescence recorded here with flow cytometry corresponds to the autofluorescence of the cells and the added inhibitors. There is significant autofluorescence due to amiloride. (**b**,**c**,**d**) THP-1 derived macrophages in serum-free RPMI 1640 medium were exposed for 1 h to the different inhibitors and then in addition with the b) *R-*, c) *S-*, and d) N*-*CNDs (*C*'_CNDs_ = 400 μg mL^–1^) for 6 more h. The fluorescence intensity *I* of the cells due to the internalized CNDs (and due to the autofluorescence of cells and inhibitors) was normalized to the fluorescence intensity *I*_0_ of the control sample (cells incubated with CNDs at 37 °C without addition of inhibitors). *I* *I*_0_^–1^ < 100% indicates that the respective inhibitor blocks CND uptake and that thus the mechanism this inhibitor is blocking is a contributing uptake pathway of the CNDs. Results are shown as mean values with error bars (i.e., the corresponding standard deviations) from three independent samples (n = 3) over three independent experiments.

**
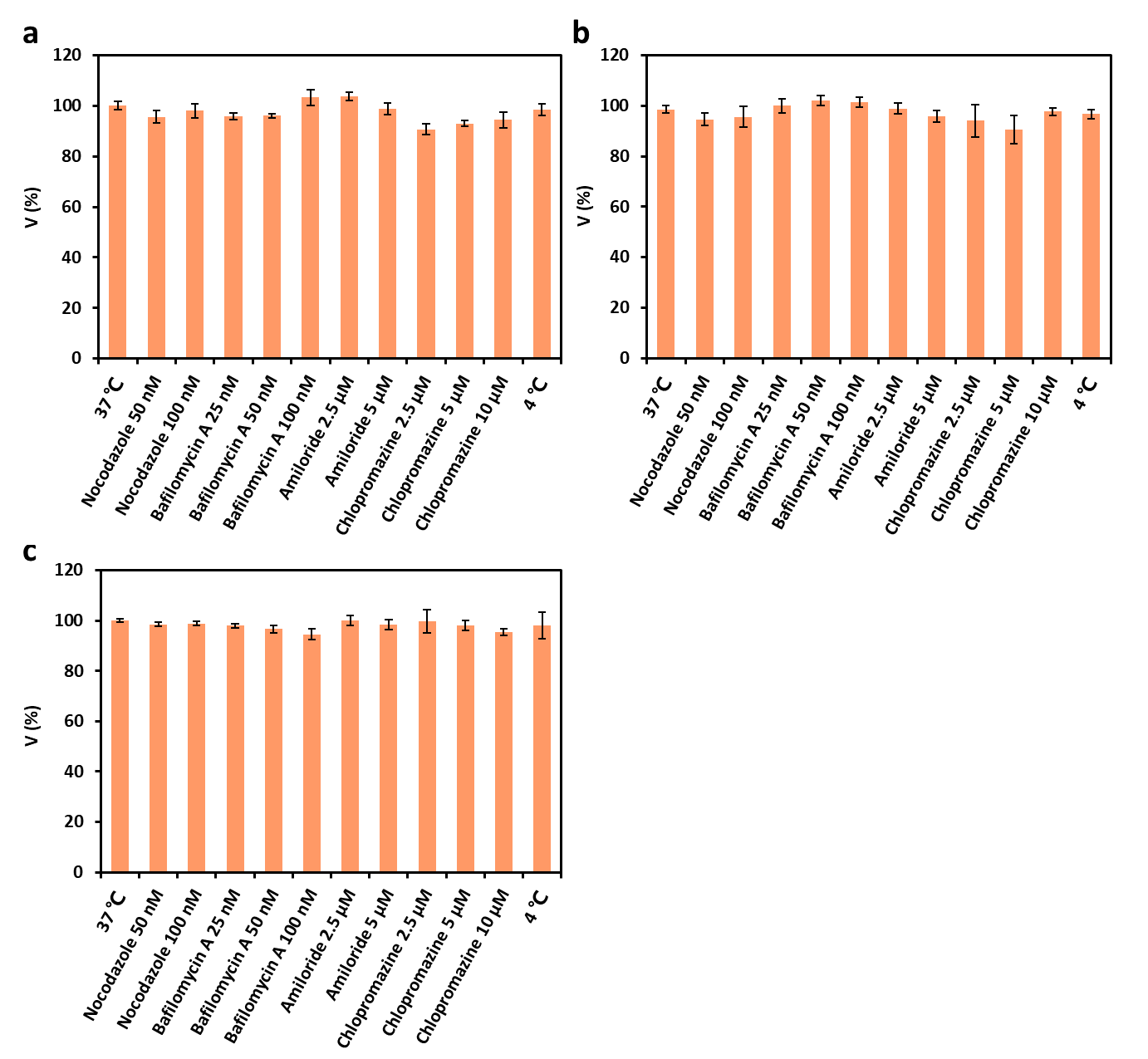
**

**Supplementary Figure 74. Cell viability V of Hela cells after exposed to cellular uptake inhibitors for 1 h followed by 6 h incubation with CNDs.** Cells were exposed to (**a**) *R-*, (**b**) *S-* or (**c**) N*-*CNDs at a concentration of *C*'_CNDs_ = 400 μg mL^–1^ in 10% FBS containing DMEM medium as measured by the resazurin assay. Resazurin was added at a final concentration of 0.025 mg mL^–1^ and the cells were further incubated for 4 h before analysis. Results are shown as mean values with error bars (i.e., the corresponding standard deviations) from three independent samples (n = 3) over three independent experiments.


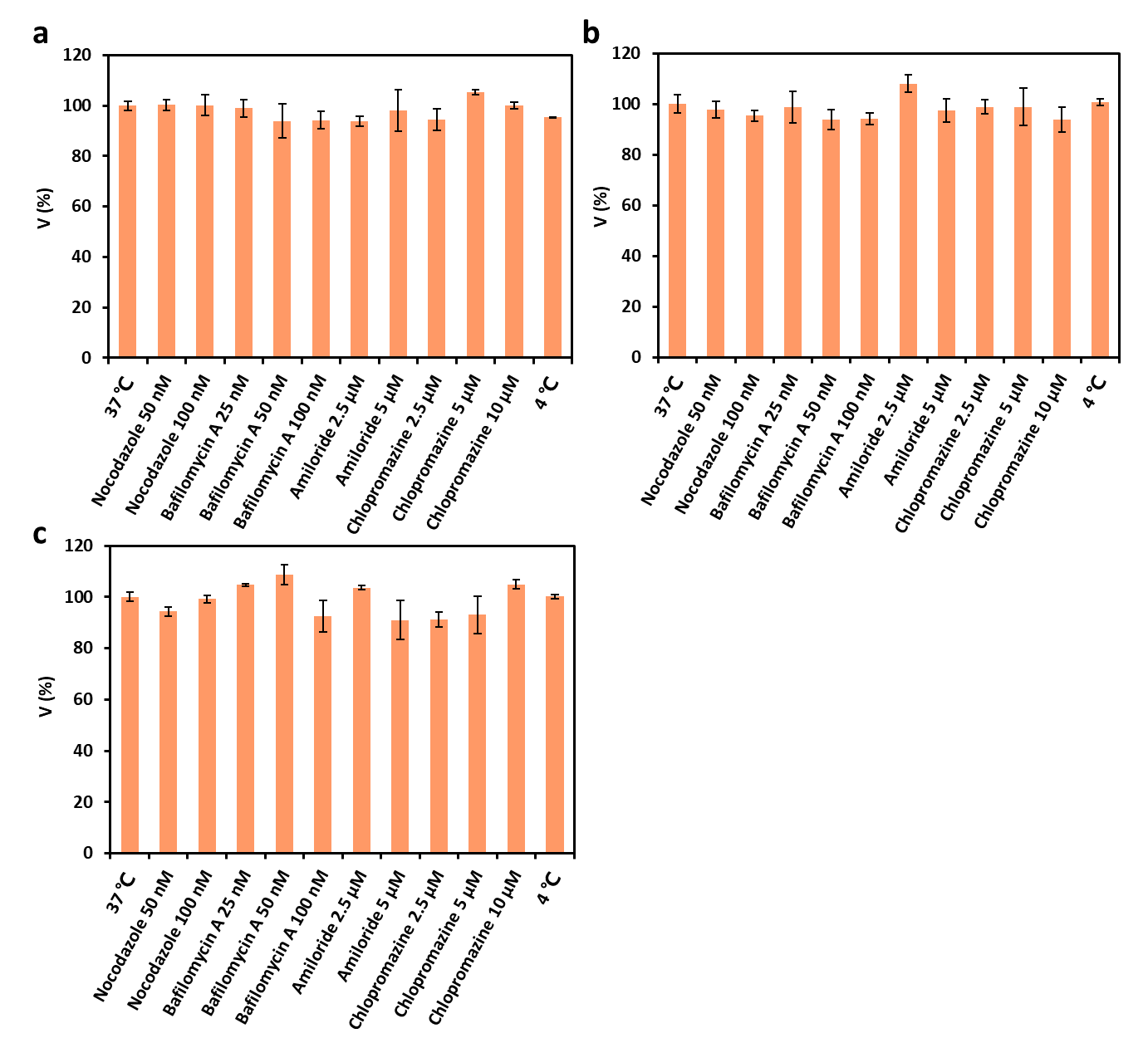


**Supplementary Figure 75. Cell viability V of Hela cells after exposed to cellular uptake inhibitors for 1 h followed by 6 h incubation with CNDs.** Cells were exposed to (**a**) *R-*, (**b**) *S-* or (**c**) N*-*CNDs at a concentration of *C*'_CNDs_ = 400 μg mL^–1^ in serum-free DMEM medium as measured by the resazurin assay. Resazurin was added at a final concentration of 0.025 mg mL^–1^ and the cells were further incubated for 4 h before analysis. Results are shown as mean values with error bars (i.e., the corresponding standard deviations) from three independent samples (n = 3) over three independent experiments.


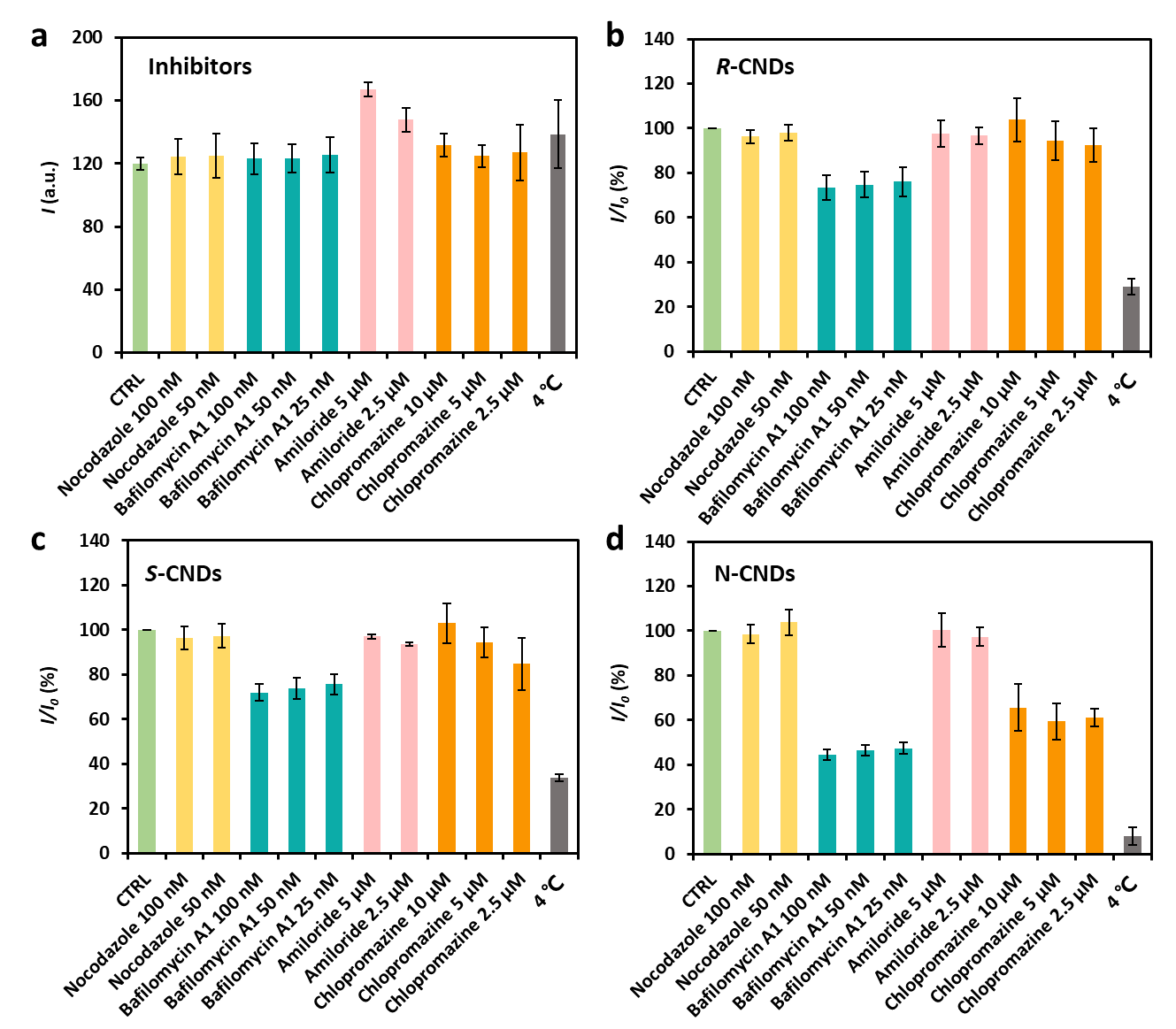


**Supplementary Figure 76.** **Analysis of cell uptake pathway trough flow cytometry.** (**a**) Fluorescence intensity *I* of Hela cells in 10% FBS containing DMEM medium after exposure to different inhibitors for 7 h. The control (CTRL) are cells to which no inhibitor had been added. As negative control cells without added inhibitors were incubated at 4 °C instead of 37 °C. The fluorescence recorded here with flow cytometry corresponds to the autofluorescence of the cells and the added inhibitors. There is significant autofluorescence due to amiloride. (**b**,**c**,**d**) THP-1 derived macrophages in 10% FBS containing DMEM medium were exposed for 1 h to the different inhibitors and then in addition with the (**b**) *R-*, (**c**) *S-*, and (**d**) N*-*CNDs (*C*'_CNDs_ = 400 μg mL^–1^) for 6 more h. The fluorescence intensity *I* of the cells due to the internalized CNDs (and due to the autofluorescence of cells and inhibitors) was normalized to the fluorescence intensity *I*_0_ of the control sample (cells incubated with CNDs at 37 °C without addition of inhibitors). *I* *I*_0_^–1^ < 100% indicates that the respective inhibitor blocks CND uptake and that thus the mechanism this inhibitor is blocking is a contributing uptake pathway of the CNDs. Results are shown as mean values with error bars (i.e., the corresponding standard deviations) from three independent samples (n = 3) over three independent experiments.

**
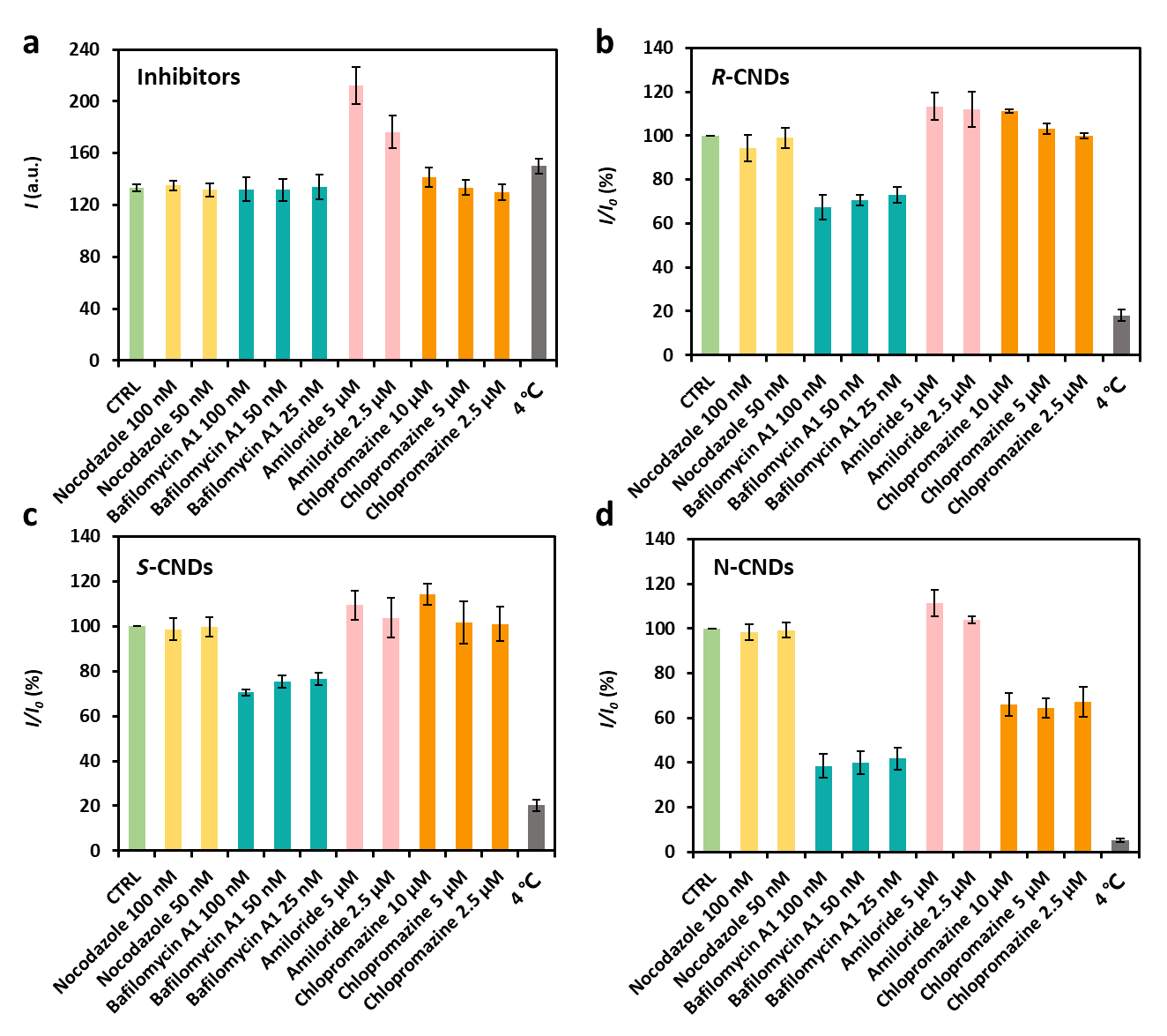
**

**Supplementary Figure 77**. **Analysis of cell uptake pathway trough flow cytometry.** (**a**) Fluorescence intensity *I* of Hela cells in serum-free DMEM medium after exposure to different inhibitors for 7 h. The control (CTRL) are cells to which no inhibitor had been added. As negative control cells without added inhibitors were incubated at 4 °C instead of 37 °C. The fluorescence recorded here with flow cytometry corresponds to the autofluorescence of the cells and the added inhibitors. There is significant autofluorescence due to amiloride. (**b**,**c**,**d**) THP-1 derived macrophages in serum-free DMEM medium were exposed for 1 h to the different inhibitors and then in addition with the (**b**) *R-*, (**c**) *S-*, and (**d**) N*-*CNDs (*C*'_CNDs_ = 400 μg mL^–1^) for 6 more h. The fluorescence intensity *I* of the cells due to the internalized CNDs (and due to the autofluorescence of cells and inhibitors) was normalized to the fluorescence intensity *I*_0_ of the control sample (cells incubated with CNDs at 37 °C without addition of inhibitors). *I* *I*_0_^–1^ < 100% indicates that the respective inhibitor blocks CND uptake and that thus the mechanism this inhibitor is blocking is a contributing uptake pathway of the CNDs. Results are shown as mean values with error bars (i.e., the corresponding standard deviations) from three independent samples (n = 3) over three independent experiments.

**Supplementary Tables**

**Supplementary Table 1. Counting of CNDs with AFM.**

| CND type | Sample number | *A*_scan_  (μm^2^) | *C*_CNDs_  (μg mL^–1^) | *N*_CNDs_ | < *N*_CNDs_ > | Δ*N*_CNDs_ | Δ*N*_CNDs_ < *N*_CNDs_ >^–1^ | Theoretical  < *n*_CNDs_ >  (μm^–2^) | Calculated  < *n*_CNDs_ >  (μm^–2^) |
| --- | --- | --- | --- | --- | --- | --- | --- | --- | --- |
| *R*-CND | 1 | 25 | 327 | 35 | 41.7 | 6.1 | 0.15 | 1.73×10^6^ | 1.67 |
| *R*-CND | 2 | 25 | 327 | 43 |  |  |  |  |  |
| *R*-CND | 3 | 25 | 327 | 47 |  |  |  |  |  |
| *R*-CND | 4 | 25 | 108 | 18 | 15.7 | 2.5 | 0.16 | 4.01×10^5^ | 0.63 |
| *R*-CND | 5 | 25 | 108 | 16 |  |  |  |  |  |
| *R*-CND | 6 | 25 | 108 | 13 |  |  |  |  |  |
| *S*-CND | 1 | 25 | 363 | 9 | 8.7 | 2.5 | 0.28 | 1.20×10^6^ | 0.35 |
| *S*-CND | 2 | 25 | 363 | 11 |  |  |  |  |  |
| *S*-CND | 3 | 25 | 363 | 6 |  |  |  |  |  |
| *S*-CND | 4 | 25 | 121 | 8 | 7.0 | 1.7 | 0.24 | 4.99×10^5^ | 0.28 |
| *S*-CND | 5 | 25 | 121 | 8 |  |  |  |  |  |
| *S*-CND | 6 | 25 | 121 | 5 |  |  |  |  |  |

**Supplementary Table 2. Counting of CNDs with TEM.**

| CND type | Sample number | *A*_scan_  (nm^2^) | *c*_CNDs_  (μM) | *N*_CNDs_ | *n*_CNDs_  (μm^–2^) | < *n*_CNDs_ >  (μm^–2^) | Δ*n*_CNDs_  (μm^–2^) | Δ*n*_CNDs_ < *n*_CNDs_ >^–1^ |
| --- | --- | --- | --- | --- | --- | --- | --- | --- |
| *R*-CND | 1 | 1.68×10^4^ | 540 | 75 | 4464 | 1920 | 1620 | 0.84 |
| *R*-CND | 2 | 6.42×10^4^ | 540 | 141 | 2196 |  |  |  |
| *R*-CND | 3 | 1.79×10^4^ | 540 | 55 | 3073 |  |  |  |
| *R*-CND | 4 | 4.22×10^4^ | 540 | 136 | 3223 |  |  |  |
| *R*-CND | 5 | 2.59×10^5^ | 540 | 108 | 417 |  |  |  |
| *R*-CND | 6 | 2.59×10^5^ | 540 | 37 | 143 |  |  |  |
| *R*-CND | 7 | 2.59×10^5^ | 540 | 454 | 1753 |  |  |  |
| *R*-CND | 8 | 1.03×10^6^ | 540 | 96 | 93 |  |  |  |
| *S*-CND | 1 | 1.16×10^5^ | 180 | 685 | 5905 | 3014 | 1542 | 0.51 |
| *S*-CND | 2 | 1.16×10^5^ | 180 | 435 | 3750 |  |  |  |
| *S*-CND | 3 | 4.22×10^4^ | 180 | 136 | 3223 |  |  |  |
| *S*-CND | 4 | 4.22×10^4^ | 180 | 119 | 2820 |  |  |  |
| *S*-CND | 5 | 4.22×10^4^ | 180 | 136 | 3223 |  |  |  |
| *S*-CND | 6 | 4.22×10^4^ | 180 | 129 | 3057 |  |  |  |
| *S*-CND | 7 | 4.22×10^4^ | 180 | 51 | 1209 |  |  |  |
| *S*-CND | 8 | 2.59×10^5^ | 180 | 240 | 927 |  |  |  |

**Supplementary Table 3.** **Error analysis in CNDs concentration determination.** CNDs concentration was calculated by absorption and fluorescence intensity measurements for different batches of CNDs. The parameters are defined in Supplementary Figure 27.

| batch | *ß*'*_A_*_,_*_R_*_-CND_ (mL μg^–1^) | *ß*'*_A_*_,_*_S_*_-CND_ (mL μg^–1^) | *ß*'*_A_*_,N-CND_ (mL μg^–1^) | *ß*'*_I_*_,_*_R_*_-CND_ (mL μg^–1^) | *ß*'*_I_*_,_*_S_*_-CND_ (mL μg^–1^) | *ß*'*_I_*_,N-CND_ (mL μg^–1^) | Δ*ß*'*_A_*_,_*_R_*_-CND_ | Δ*ß*'*_A_*_,_*_S_*_-CND_ | Δ*ß*'*_I_*_,_*_R_*_-CND_ | Δ*ß*'*_I_*_,_*_S_*_-CND_ | Δ*ß*'*_R_*_-CND_ | Δ*ß*'*_S_*_-CND_ | Δ*C*'_CND_ *C*'_CND_^–1^ |
| --- | --- | --- | --- | --- | --- | --- | --- | --- | --- | --- | --- | --- | --- |
| 1 | 0.00178 | 0.00176 | 0.00187 | 543708 | 625975 | 712661 | 0.049 | 0.057 | 0.24 | 0.12 | 0.19 | 0.06 | 0.19 |
| 2 | 0.0014 | 0.0014 | 0.0014 | 252796 | 291874 | 338044 | 0.018 | 0.003 | 0.25 | 0.14 | 0.23 | 0.13 | 0.23 |
| 3 | 0.0014 | 0.0015 | 0.0015 | 261593 | 286144 | 323812 | 0.076 | -0.005 | 0.19 | 0.12 | 0.17 | 0.12 | 0.12 |
| 4 | 0.0015 | 0.0015 | 0.0015 | 271880 | 301066 | 323631 | 0.009 | -0.017 | 0.16 | 0.07 | 0.15 | 0.09 | 0.15 |
| 5 | 0.0015 | 0.0015 | 0.0016 | 190044 | 213179 | 308478 | 0.041 | 0.024 | 0.38 | 0.31 | 0.34 | 0.28 | 0.34 |
| mean |  |  |  |  |  |  |  |  |  |  |  |  | 0.20 |

**Supplementary Table 4.** **Correction factors *X* for CNDs concentration determination.** *X* values were calculated considering the different fluorescence intensity of the CND samples at the same adjusted concentrations.

|  | flow cytometry | | confocal microscopy | |
| --- | --- | --- | --- | --- |
| batch | *X_S_*_/_*_R_* | *X_S_*_/N_ | *X_S_*_/_*_R_* | *X_S_*_/N_ |
| 1 | 1.07 | 0.87 | 1.07 | 0.89 |
| 2 | 1.08 | 0.82 | 0.99 | 0.70 |
| 3 | 1.09 | 0.91 | 1.05 | 0.84 |
| 4 | 1.08 | 0.91 | 1.05 | 0.84 |
| 5 | 1.14 | 0.74 | 1.07 | 0.67 |

**Supplementary Table 5.** **Parameters describing the interaction of CNDs and HSA.** Apparent dissociation constant *K_D_*, maximum number *N*_max_ of HSA molecules adsorbed per CND, cooperativity parameter n, hydrodynamic radius *r*_h,0_ = of the CNDs without exposure to HSA, and maximum increase of hydrodynamic radius Δ*r*_h,max_ = *r*_h_(*c*_P_ >> *K_D_*) - *r*_h,0_ of the CNDs upon saturation with HSA. The error is the standard error as obtained by fitting of the data with the Hill-Model. In the last line the mean values ± the standard deviations (STD) of the hydrodynamic radii are listed. The data for batch #1 are also shown in Table 2 of the main manuscript. *r*_h_(0) is the experimentally determined hydrodynamic radius without added proteins, *r*_h,0_ is a fit parameter.

| batch | CND type | *K*_D_  (μM) | *N*_max_ | *n* | *r*_h_(0)  (nm) | *V*_CND_  (nm^3^) | *r*_h,0_  (nm) | Δ*r*_h,max_  (nm) | <*K*_D_>  (μM) | Δ*K*_D_ <*K*_D_>^–1^ |
| --- | --- | --- | --- | --- | --- | --- | --- | --- | --- | --- |
| 1 | R-CNDs | 22.7 ± 5.3 | 1.2 ± 0.3 | 1.2 ± 0.2 | 0.66±0.02 | 1.2 | 0.69 ±0.05 | 2.4 ± 0.1 | 32.3 | -0.30 |
| 1 | *S*-CNDs | 39.9 ± 9.5 | 1.2 ± 0.3 | 1.2 ± 0.2 | 0.7±0.04 | 1.5 | 0.73 ± 0.04 | 2.3 ± 0.1 |  | +0.24 |
| 1 | N-CNDs | 34.3 ± 10.4 | 2 ± 0.6 | 1.0 ± 0.1 | 0.79±0.05 | 2.1 | 0.73 ± 0.05 | 2.5 ± 0.1 |  | +0.06 |
| 2 | *R*-CNDs | 131 ± 35 | 1.9 ± 0.3 | 0.93 ± 0.06 | 0.83±0.17 | 2.4 | 0.75 ± 0.02 | 2.2 ± 0.02 |  |  |
| 2 | *S*-CNDs | 180 ± 65 | 1.6 ± 0.4 | 1.0 ± 0.1 | 0.73±0.04 | 1.6 | 0.78 ± 0.03 | 2.4 ± 0.03 |  |  |
| 2 | N-CNDs | ＞ 500 | 22 ± 53 | 0.97 ± 0.1 | 1.03±0.1 | 4.6 | 0.96 ± 0.04 | 2.8 ± 0.1 |  |  |
| 3 | *R*-CNDs | 181 ± 31 | 3.9 ± 0.4 | 1.0 ± 0.04 | 0.98±0.14 | 4 | 0.89 ± 0.01 | 2.8 ± 0.01 |  |  |
| 3 | *S*-CNDs | 273 ± 140 | 2 ± 0.6 | 1.0 ± 0.1 | 0.93±0.17 | 3.2 | 0.96 ± 0.03 | 2.3 ± 0.03 |  |  |
| 3 | N-CNDs | 620 ± 510 | 4.1 ± 2.0 | 0.98 ± 0.1 | 1.04±0.05 | 4.8 | 1.05 ± 0.04 | 2.5 ± 0.1 |  |  |
| mean ± STD |  |  |  |  |  |  | 0.84 ±0.03 | 2.5 ±0.1 |  |  |

**Supplementary Table 6.**  **Mean fluorescence intensity *I*’ (a.u.) per cell due to internalized *R-*, *S-* and N*-*CNDs as extracted from** **Supplementary Figure 22c.** *I*’ is calculated after background correction and adjustment for the different fluorescence intensities of the different types of CNDs as taken from Supplementary Figure 22c.

| serum supplemented condition | | | | | |
| --- | --- | --- | --- | --- | --- |
| *t*  (h) | 1 | 3 | 6 | 24 | 48 |
| *I’*(*R*-CNDs) (a.u.) | 313.8±27.3 | 466.1±37.7 | 570.8±57.1 | 724.7±48.8 | 797.7±60.5 |
| *I’*(*S*-CNDs)  (a.u.) | 325.7±49.7 | 458.7±37.9 | 607.7±55.9 | 827.7±68.4 | 907.3±82.4 |
| *I’*(N-CNDs)  (a.u.) | 95.1±5.5 | 158.4±14.5 | 225.9±24.6 | 297.9±17.2 | 380.7±29.9 |
| serum free condition | | | | | |
| *t*  (h) | 1 | 3 | 6 | 24 | 48 |
| *I’*(*R*-CNDs)  (a.u.) | 297.9±23.9 | 474.4±35.1 | 627.3±15.2 | 916.3±35.1 | 971.9±34.4 |
| *I’*(*S*-CNDs)  (a.u.) | 333.3±45.7 | 498.0±48.1 | 669.7±9.3 | 1055.0±43.4 | 1065.0±41.2 |
| *I’*(N-CNDs)  (a.u.) | 87.9±7.4 | 168.1±25.1 | 244.3±17.3 | 359.3±20.9 | 378.3±19.2 |

**Supplementary Table 7. Mean fluorescence intensity *I*’ (a.u.) per cell due to internalized *R-*, *S-* and N*-*CNDs as extracted from** **Supplementary Figure 24c**. *I*’ is calculated after background correction and adjustment for the different fluorescence intensities of the different types of CNDs as taken from Supplementary Figure 24c.

| serum supplemented condition | | | | | |
| --- | --- | --- | --- | --- | --- |
| *t*  (h) | 1 | 3 | 6 | 24 | 48 |
| *I’*(*R*-CNDs)  (a.u.) | 57.4±16.3 | 94.0±26.9 | 143.8±24.0 | 250.7±57.4 | 479.7±57.7 |
| *I’*(*S*-CNDs)  (a.u.) | 85.2±22.4 | 115.7±27.8 | 216.7±39.6 | 322.0±59.0 | 600.7±60.9 |
| *I’*(N-CNDs)  (a.u.) | 38.6±13.3 | 69.7±14.5 | 105.1±23.7 | 214.5±49.3 | 455.5±18.1 |
| serum free condition | | | | | |
| *t*  (h) | 1 | 3 | 6 | 24 | 48 |
| *I’*(*R*-CNDs)  (a.u.) | 59.5±11.9 | 99.4±29.7 | 155.7±11.4 | 304.9±51.5 | 492.3±12.0 |
| *I’*(*S*-CNDs)  (a.u.) | 110.2±16.8 | 169.5±37.3 | 229.8±22.6 | 430.5±46.9 | 653.7±61.8 |
| *I’*(N-CNDs)  (a.u.) | 39.7±11.4 | 87.4±35.9 | 97.5±19.5 | 215.8±28.5 | 149.0±19.2 |

**Supplementary Table 8. Mean fluorescence intensity *I*’ (a.u.) per cell due to internalized *R-*, *S-* and N*-*CNDs as extracted from** **Supplementary Figure 12.** *I*' is calculated after adjustment for the different fluorescence intensities of the different types of CNDs as taken from Supplementary Figure 12. Note that due to low background no background correction was carried out.

| serum supplemented condition | | | | | |
| --- | --- | --- | --- | --- | --- |
| *t*  (h) | 1 | 3 | 6 | 24 | 48 |
| *I’*(*R*-CNDs)  (a.u.) | 5.1±0.9 | 7.8±0.6 | 11.5±2.4 | 18.5±6.7 | 30.2±6.2 |
| *I’*(*S*-CNDs)  (a.u.) | 14.5±1.6 | 19.3±1.9 | 21.6±5.2 | 39.6±15.6 | 61.3±13.5 |
| *I’*(N-CNDs)  (a.u.) | 3.0±1.1 | 5.9±1.5 | 5.4±1.1 | 7.9±2.6 | 14.2±2.7 |
| serum free condition | | | | | |
| *t*  (h) | 1 | 3 | 6 | 24 | 48 |
| *I’*(*R*-CNDs)  (a.u.) | 8.6±1.8 | 12.4±2.8 | 18.6±8.4 | 19.8±5.5 | 46.4±3.0 |
| *I’*(*S*-CNDs)  (a.u.) | 21.2±4.6 | 23.6±6.5 | 34.9±8.3 | 63.5±20.6 | 112.5±10.3 |
| *I’*(N-CNDs)  (a.u.) | 3.3±1.3 | 5.7±2.3 | 7.3±5.2 | 14.3±4.4 | 21.5±2.4 |

**Supplementary Table 9. Mean fluorescence intensity *I*’ (a.u.) per cell due to internalized *R-*, *S-* and N*-*CNDs as extracted from** **Supplementary Figure 13c**. *I*’ is calculated after background correction and adjustment for the different fluorescence intensities of the different types of CNDs as taken from Supplementary Figure 13c.

| serum supplemented condition | | | | | |
| --- | --- | --- | --- | --- | --- |
| *C*'_CNDs_  (μg mL^–1^) | 50 | 100 | 200 | 300 | 400 |
| *I’*(*R-*CNDs)  (a.u.) | 18.4±2.1 | 28.8±9.4 | 60.2±3.5 | 103.1±0.6 | 151.9±14.8 |
| *I’*(*S*-CNDs)  (a.u.) | 19.5±6.5 | 30.5±2.5 | 54.5±0.5 | 117.5±3.5 | 236.0±42 |
| *I’*(N-CNDs)  (a.u.) | 11.7±0.2 | 21.5±7.6 | 40.3±4.3 | 80.3±9.8 | 113.6±20.1 |
| serum free condition | | | | | |
| *C*'_CNDs_  (μg mL^–1^) | 50 | 100 | 200 | 300 | 400 |
| *I’*(*R*-CNDs)  (a.u.) | 15.2±10.9 | 28.2±1.2 | 48.9±6.7 | 99.3±8.6 | 151.4±2.0 |
| *I’*(*S*-CNDs)  (a.u.) | 19.5±6.5 | 38.0±3.0 | 73.0±3.0 | 155.5±13.5 | 241.0±24.0 |
| *I’*(N-CNDs)  (a.u.) | 6.2±2.1 | 15.8±4.3 | 36.6±1.9 | 65.7±5.6 | 99.2±17.3 |

**Supplementary Table 10**. **Error analysis in concentration determination by absorption (A_405_(C_CNDs_)) and fluorescence intensity (I_max_(C_CNDs_)) measurements.** The parameters are defined in Supplementary Figure 6.

| batch | *ß_A_*_,_*_R_*_-CND_  (mL μg^–1^) | *ß_A_*_,_*_S_*_-CND_  (mL μg^–1^) | *ß_A_*_,N-CND_  (mL μg^–1^) | *ß_I_*_,_*_R-_*_CND_  (mL μg^–1^) | *ß_I_*_,_*_S_*_-CND_  (mL μg^–1^) | *ß_I_*_,N-CND_  (mL μg^–1^) | Δ*ß_A_*_,_*_R_*_-CND_ | Δ*ß_A,S_*_-CND_ | Δ*ß_I_*_,_*_R-_*_CND_ | Δ*ß_I_*_,_*_S_*_-CND_ | Δ*ß_R_*_-CND_ | Δ*ß_S_*_-CND_ | Δ*C*_CNDs_ *C*_CNDs_^–1^ |
| --- | --- | --- | --- | --- | --- | --- | --- | --- | --- | --- | --- | --- | --- |
| 1 | 0.00013 | 0.00018 | 0.00022 | 569422 | 842279 | 1187647 | 0.43 | 0.17 | 0.52 | 0.29 | 0.09 | 0.12 | 0.12 |

**Supplementary Table 11.** **Fits results for the autocorrelation functions G(*τ*) reported in Supplementary Figure 35.** Diffusion times *τ*_D_, the corresponding diffusion coefficients *D* and hydrodynamic radii *r*_h_ are reported for G(*τ*) recorded at different *c*_HSA_.

| *c*_HSA_  (μM) | *τ*_D_  (µs) | *D*  (μm^2^ s^–1^) | *r*_h_  (nm) |
| --- | --- | --- | --- |
| 0 | 25 ± 3 | 308 ± 18 | 0.7 ± 0.04 |
| 0.001 | 31 ± 2.3 | 289 ± 23 | 0.75 ± 0.06 |
| 0.01 | 35 ± 1 | 291 ± 11 | 0.75 ± 0.02 |
| 0.02 | 29 ± 1 | 304 ± 21 | 0.72 ± 0.05 |
| 0.05 | 28 ± 1 | 306 ± 6 | 0.71 ± 0.01 |
| 0.1 | 29 ± 2.5 | 285 ± 10 | 0.76 ± 0.03 |
| 0.5 | 36 ± 4 | 284 ± 21 | 0.77 ± 0.05 |
| 1 | 38 ± 4.3 | 224 ± 5 | 0.97 ± 0.02 |
| 10 | 57 ± 1 | 138 ± 11 | 1.5 ± 0.12 |
| 20 | 86 ± 1.2 | 98 ± 5 | 2.2 ± 0.1 |
| 50 | 120 ± 3 | 82 ± 3 | 2.67 ± 0.1 |
| 100 | 113 ± 4 | 79 ± 2 | 2.7 ± 0.07 |

**Supplementary Table 12.** **Mean fluorescence intensity *I*’ (a.u.) per cell due to internalized *R-*, *S-* and N*-*CNDs as extracted from Supplementary Figure 45c**. *I*’ is calculated after background correction and adjustment for the different fluorescence intensities of the different types of CNDs as taken from Supplementary Figure 45c.

| serum supplemented condition | | | | | |
| --- | --- | --- | --- | --- | --- |
| *C*'_CNDs_  (μg mL^–1^) | 50 | 100 | 200 | 300 | 400 |
| *I’*(*R*-CNDs)  (a.u.) | 8.7±5.8 | 19.9±4.9 | 29.9±7.9 | 44.1±3.8 | 61.0±7.5 |
| *I’*(*S*-CNDs)  (a.u.) | 22.3±3.8 | 27.0±1.4 | 47.0±9.9 | 74.7±7.5 | 102.7±7.5 |
| *I’*(N-CNDs)  (a.u.) | 6.9±6.8 | 14.4±2.1 | 18.8±3.4 | 27.6±1.8 | 43.3±7.3 |
| serum free condition | | | | | |
| *C*'_CNDs_  (μg mL^–1^) | 50 | 100 | 200 | 300 | 400 |
| *I’*(*R*-CNDs)  (a.u.) | 7.6±5.5 | 15.1±7.5 | 22.7±4.9 | 39.0±8.4 | 50.6±12.1 |
| *I’*(*S*-CNDs)  (a.u.) | 14.3±5.2 | 20.7±3.7 | 34.0±5.4 | 71.3±9.0 | 102.3±10.1 |
| *I’*(N-CNDs)  (a.u.) | 10.5±6.7 | 14.3±4.5 | 19.9±4.6 | 30.3±3.2 | 42.7±6.7 |

**Supplementary Table 13.** **Mean fluorescence intensity *I*’ (a.u.) per cell due to internalized *R-*, *S-* and N*-*CNDs as extracted from Supplementary Figure 47c**. *I*’ is calculated after background correction and adjustment for the different fluorescence intensities of the different types of CNDs as taken from Supplementary Figure 47c.

| serum supplemented condition | | | | | |
| --- | --- | --- | --- | --- | --- |
| *C*'_CNDs_  (μg mL^–1^) | 50 | 100 | 200 | 300 | 400 |
| *I’*(*R*-CNDs)  (a.u.) | 9.4±2.9 | 24.2±7.4 | 45.9±5.3 | 71.9±2.7 | 107.9±5.3 |
| *I’*(*S*-CNDs)  (a.u.) | 24.7±3.3 | 33.3±3.3 | 54.3±4.7 | 88.0±18.4 | 131.3±0.5 |
| *I’*(N-CNDs)  (a.u.) | 11.2±5.6 | 19.9±2.6 | 29.6±3.6 | 62.0±7.4 | 77.1±3.8 |
| serum free condition | | | | | |
| *C*'_CNDs_  (μg mL^–1^) | 50 | 100 | 200 | 300 | 400 |
| *I’*(*R*-CNDs)  (a.u.) | 7.9±5.1 | 20.9±10.3 | 39.8±7.8 | 76.6±8.3 | 93.9±12.4 |
| *I’*(*S*-CNDs)  (a.u.) | 14.7±9.5 | 25.3±4.1 | 57.3±8.2 | 99.7±7.0 | 169.7±1.5 |
| *I’*(N-CNDs)  (a.u.) | 8.8±7.7 | 16.9±4.6 | 28.3±3.6 | 58.9±8.2 | 118.2±22.8 |

**Supplementary Table 14. Mean fluorescence intensity *I*’ (a.u.) per cell due to internalized *R-*, *S-* and N*-*CNDs as extracted from Supplementary Figure 49c.** *I*’ is calculated after background correction and adjustment for the different fluorescence intensities of the different types of CNDs as taken from Supplementary Figure 49c.

| serum supplemented condition | | | | | |
| --- | --- | --- | --- | --- | --- |
| *C*'_CNDs_  (μg mL^–1^) | 50 | 100 | 200 | 300 | 400 |
| *I’*(*R-*CNDs)  (a.u.) | 27.1±12.1 | 59.6±14.5 | 120.3±9.1 | 204.1±18.2 | 226.1±11.0 |
| *I’*(*S-*CNDs)  (a.u.) | 24.7±15.1 | 59.7±13.7 | 131.7±9.4 | 239.7±12.8 | 324.3±17.9 |
| *I’*(N-CNDs)  (a.u.) | 20.7±11.0 | 42.6±5.5 | 84.1±11.3 | 145.9±30.1 | 170.4±29.8 |
| serum free condition | | | | | |
| *C*'_CNDs_  (μg mL^–1^) | 50 | 100 | 200 | 300 | 400 |
| *I’*(*R-*CNDs)  (a.u.) | 23.2±11.0 | 46.3±13.9 | 88.1±8.5 | 195.7±14.4 | 272.6±19.6 |
| *I’*(*S-*CNDs)  (a.u.) | 29.3±4.5 | 60.0±9.2 | 128.0±12.1 | 252.7±24.1 | 384.0±19.0 |
| *I’*(N-CNDs)  (a.u.) | 17.5±3.7 | 43.7±2.2 | 80.9±9.3 | 148.3±9.6 | 175.6±3.6 |

**Supplementary Table 15. Mean fluorescence intensity *I*’ (a.u.) per cell due to internalized *R-*, *S-* and N*-*CNDs as extracted from Supplementary Figure 51c**. *I*’ is calculated after background correction and adjustment for the different fluorescence intensities of the different types of CNDs as taken from Supplementary Figure 51c.

| serum supplemented condition | | | | | |
| --- | --- | --- | --- | --- | --- |
| *C*'_CNDs_  (μg mL^–1^) | 50 | 100 | 200 | 300 | 400 |
| *I’*(*R*-CNDs)  (a.u.) | 66.5±13.2 | 116.4±19.9 | 204.1±16.8 | 356.6±4.3 | 452.6±22.3 |
| *I’*(*S*-CNDs)  (a.u.) | 71.3±16.0 | 128.7±23.6 | 214.0±7.1 | 442.3±27.3 | 567.3±17.4 |
| *I’*(N-CNDs)  (a.u.) | 53.5±9.2 | 103.1±15.9 | 192.2±27.7 | 327.4±25.3 | 430.7±28.7 |
| serum free condition | | | | | |
| *C*'_CNDs_  (μg mL^–1^) | 50 | 100 | 200 | 300 | 400 |
| *I’*(*R*-CNDs)  (a.u.) | 34.4±16.5 | 78.8±20.3 | 182.0±10.6 | 343.5±35.7 | 436.3±7.6 |
| *I’*(*S-*CNDs)  (a.u.) | 55.7±15.9 | 110.3±18.2 | 226.3±29.9 | 421.0±31.6 | 598.3±10.0 |
| *I’*(N-CNDs)  (a.u.) | 37.8±11.5 | 69.6±24.2 | 159.2±31.9 | 274.2±22.3 | 367.1±29.1 |

**Supplementary Table 16.** **Uptake pathway inhibitors concentration *c* used for the tests of cellular viability.**

| Inhibitor | *c*_1_ | *c*_2_ | *c*_3_ | *c*_4_ | *c*_5_ | *c*_6_ |
| --- | --- | --- | --- | --- | --- | --- |
| Nocodazole | 1 nM | 10 nM | 25 nM | 50 nM | 100 nM | 1000 nM |
| Bafilomycin A1 | 10 nM | 25 nM | 50 nM | 100 nM |  |  |
| Amiloride | 0.5 μM | 1 μM | 2.5 μM | 5 μM |  |  |
| Chlopromazine | 1 μM | 2.5 μM | 5 μM | 10 μM |  |  |

**Supplementary Table 17.**  **Uptake pathway inhibitors concentration *c* used for the tests of cellular viability after CNDs incubation.**

| Inhibitor | c1 | c2 | c3 |
| --- | --- | --- | --- |
| Nocodazole | 50 nM | 100 nM |  |
| Bafilomycin A1 | 25 nM | 50 nM | 100 nM |
| Amiloride | 2.5 μM | 5 μM |  |
| Chlopromazine | 2.5 μM | 5 μM | 10 μM |

**Supplementary References**

1 Dordevic, L. *et al.* Design principles of chiral carbon nanodots help convey chirality from molecular to nanoscale level. *Nat. Commun.* **9**, 3442 (2018).
